# A universal synthetic strategy for noncentrosymmetric lead oxyhalide frameworks with strong second-harmonic generation

**DOI:** 10.1039/d6sc02530g

**Published:** 2026-08-25

**Authors:** Chen Sun, Xingxing Jiang, Feiyuan Gong, Xiaotian Zhang, Chao Wu, Zheshuai Lin, Chi Zhang, Honghan Fei

**Affiliations:** a Shanghai Key Laboratory of Chemical Assessment and Sustainability, School of Chemical Science and Engineering, Tongji University 1239 Siping Rd. Shanghai 200092 China wuc@tongji.edu.cn chizhang@tongji.edu.cn fei@tongji.edu.cn; b Technical Institute of Physics and Chemistry, Chinese Academy of Sciences Beijing 100190 China

## Abstract

Hybrid lead halides possess a unique combination of structural flexibility, compositional tunability and bandgap adjustability, making them promising candidates for nonlinear optical (NLO) applications. However, realizing strong second-harmonic generation (SHG) responses remains a significant challenge due to their inherent ionic architecture, which favors ideal PbX_6_ octahedral geometry and hinders oriented alignment of asymmetric primitives. Herein, we present an inorganic–organic dual-site synergistic strategy that combines π-conjugated aromatic units and stereoactive inorganic building blocks to induce noncentrosymmetry and substantial macroscopic polarization. The well-aligned coordination between Pb^2+^ centers and *meta*-dicarboxylates facilitates the construction of a new family of fifteen noncentrosymmetric lead oxyhalide frameworks. These frameworks featuring NLO-active [PbX_2_O_6_] (X = Cl^−^, Br^−^, I^−^) units allow for bandgap modulation dependent on the halide species, and the ordered arrangement of 5-functionalized isophthalate linkers directs the primitive superposition. Among them, the Br-substituted isophthalate-based lead iodide exhibits record-level performance for hybrid lead halides: a phase-matching SHG response of 11.0 × KH_2_PO_4_ at 1064 nm, a birefringence of 0.179 at 546 nm, and a corresponding UV cutoff edge. Moreover, this synthetic strategy establishes direct coordination linkage between organic phosphorescent centers and inorganic light-harvesting lead halide motifs, enabling tunable long-lived afterglow emission up to 7.36 ms.

## Abstract 2

An inorganic–organic dual-site strategy enables 15 isoreticular noncentrosymmetric lead halide frameworks. Oriented [PbX_2_O_6_]-isophthalate coupling achieves phase-matching SHG up to 11 × KDP at 1064 nm, the highest among hybrid lead halides.

## Graphical abstract

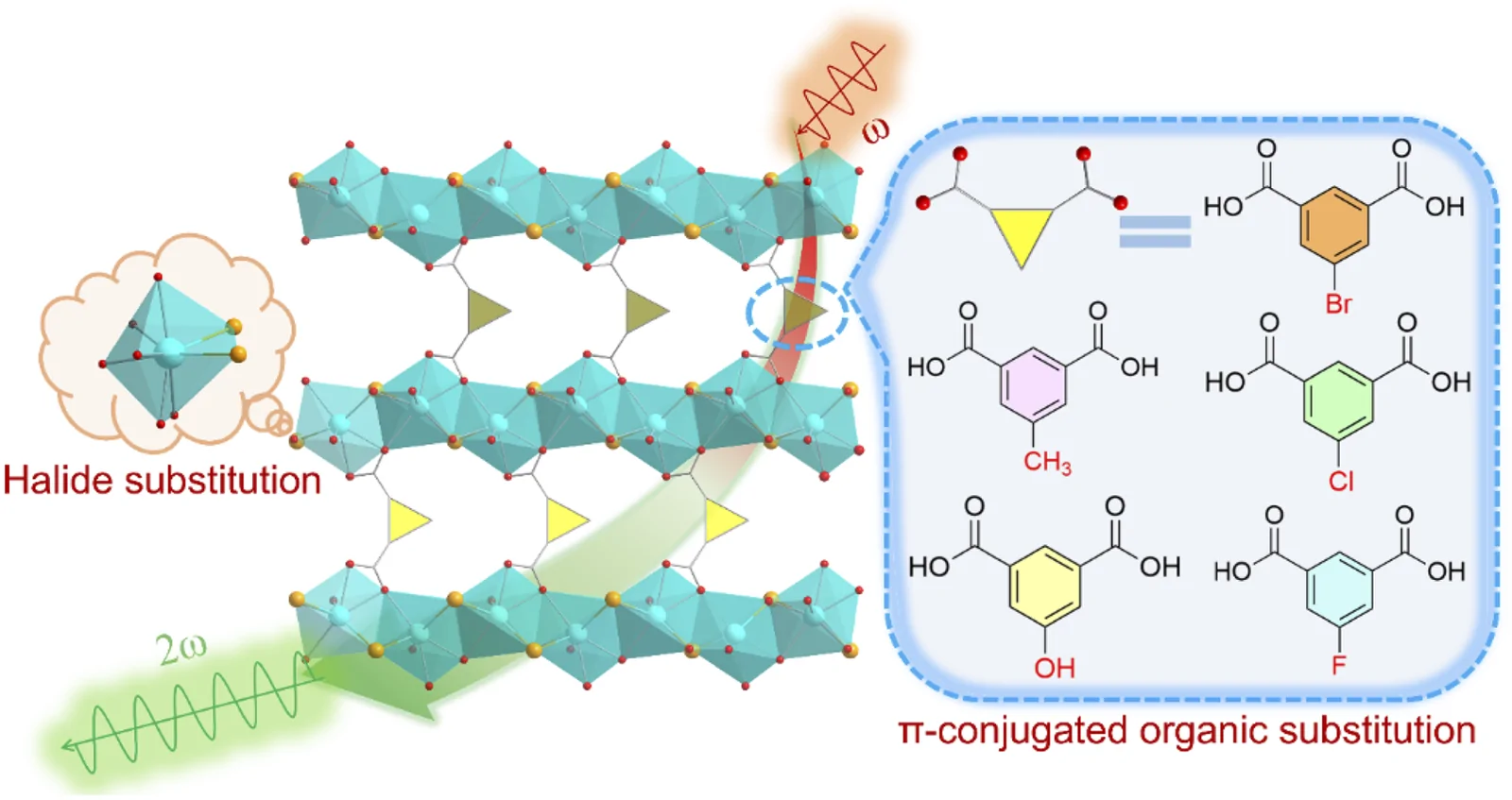

## Introduction

Nonlinear optical (NLO) materials have garnered considerable attention, owing to their capability to broaden the wavelength ranges of laser-generated output radiation.^1–3^ Among these, second harmonic generation (SHG) is one of the widely-applied second-order NLO processes, playing a critical role in a wide range of modern optical technologies, including optical communication, laser printing, biomedical imaging, ultrafast spectroscopy and sensor technology.^4,5^ Non-centrosymmetric (NCS) crystal structures are an essential prerequisite for SHG activity, but most thermodynamically stable structures tend to be centrosymmetric and inherently SHG-inactive, making the discovery of new NCS structures to be challenging.^6,7^ The high-performance NLO materials have stringent criteria to follow, including large first-order NLO hyperpolarizability, suitable birefringence (Δ*n*) for phase-matching, wide optical transmission windows, structural robustness and the feasibility of growing large crystals.^8,9^ Consequently, one of the key challenges in this field is to develop rational synthetic strategies that allow for the simultaneous and precise engineering of both the inorganic and organic components within a well-defined framework, which is essential to maximize SHG performance in hybrid materials.

Hybrid lead halides represent an emerging class of crystalline semiconductors with remarkable chemical and structural diversity, offering a promising platform for the development of NLO materials.^10–13^ Their key advantages include highly tunable architectures enabled by inorganic–organic design,^14–16^ broad optical transparency spanning the visible and near-infrared region,^17,18^ and cooperative interactions between polarizable lead-halide moieties and functional organic primitives.^11,19^ In this context, polar organic cations, even when achiral, can interact with metal-halide layers through hydrogen-bonding and/or ionic interactions to favor polar or NCS arrangements, as demonstrated in several 2D lead halide perovskites with SHG activity.^20–22^ Beyond such strategies, the incorporation of chiral ligands provides a more direct route to NCS crystallization by transferring molecular asymmetry into the inorganic lattice, which can further enhance NLO activity.^23–25^ On the inorganic side, stereochemically active *n*s^2^ lone pairs in metal cations provide an intrinsic driving force for structural distortion and non-centrosymmetric framework formation. The expression of *n*s^2^ lone pairs is closely related to the identity of the metal center, generally following the trend of Ge^2+^ > Sn^2+^ > Pb^2+^, while Pb^2+^ remains particularly attractive for NLO materials owing to its suitable ionic radius and flexible coordination chemistry.^26^ The degree of *n*s^2^ lone-pair expression depends sensitively on the metal center and local coordination environment. In conventional Pb-based halide perovskites, the stereochemical activity of Pb^2+^ lone pairs is generally enhanced with lighter halides (Cl > Br > I), whereas the coordination environment provides additional opportunities to tune structural distortion and polarization. Although Pb^2+^ lone-pair activity in lead-halide polyhedra can produce NLO-active [PbX_6_] units, simple Pb–X coordination alone rarely produces pronounced octahedral distortions required for strong second-order responses.^27–29^ Moreover, their soft and discrete ionic architectures often result in irregular alignment and loose structural packing of primitives, thereby suppressing the macroscopic NLO response.^30^

Introducing significant distortions into ideal PbX_6_ octahedra is a fundamental strategy for activating stereochemical activity and local polarity in hybrid lead halides.^31–34^ This can be achieved through elemental substitution, such as partial cross-substitution between oxygen (O^2−^) and halogen (X^−^) anions in structures of Cs_3_Pb_2_(CH_3_COO)_2_X_5_ and Pb_2_BO_3_X, which generates asymmetric, NLO-active lead oxyhalide building units.^35,36^ The substantial differences in electronegativity, ionic radius, coordination mode and polarizability between O^2−^ and X^−^ anions induce the formation of asymmetric, polar Pb^2+^-centered polyhedra that function as the essential asymmetric units.^37–39^ Specifically, the larger mismatch between O^2−^ and I^−^, can induce pronounced asymmetry in Pb-centered polyhedra and generate enhanced local polarization, leading to an increasing polarization trend of I > Br > Cl. The acentric and polar design can be further optimized by incorporating planar inorganic π-conjugated anions (*e.g.*, BO_3_^3−^, CO_3_^2−^, and NO_3_^−^), which possess large hyperpolarizability and polarizability anisotropy as effective building blocks in NLO-active hybrid lead halides.^40–42^ However, while aromatic π-conjugated anions (*e.g.*, cyanurate [C_3_N_3_O_3_]^3−^) offer distinct advantages (*e.g.*, stronger electron delocalization and greater structural flexibility) that address the inherent rigidity of inorganic anions in the tailored design of NLO materials,^43–45^ their weak and poorly directional O/N donor sites simultaneously hinder the ordered and oriented arrangements through coordination-driven assembly, especially within hybrid lead halides.^46,47^ Thus, it is crucial and challenging to develop a general strategy for constructing a new family of NLO-active hybrid lead halides based on well-aligned aromatic π-conjugated anions.

**Scheme 1:**
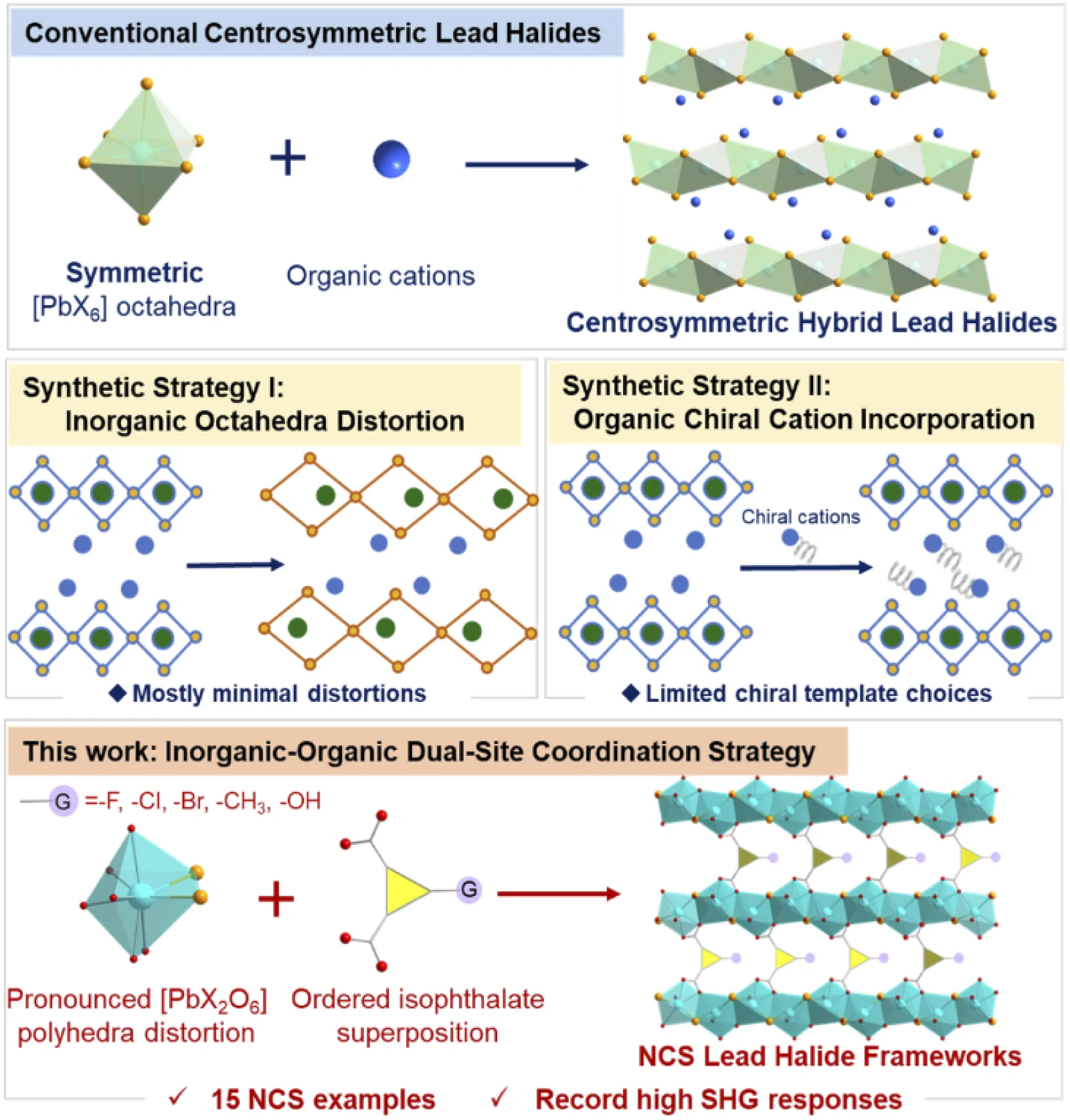
Schematic illustration of our general synthetic strategy for a new family of NCS lead oxyhalide frameworks. Previous work: conventional synthetic strategies to achieve NCS hybrid lead halides. This work: our inorganic–organic dual-site strategy for constructing highly stable NCS lead oxyhalide frameworks.

Based on the above considerations, this work identified five V-shaped isophthalates as a new, versatile class of NLO-active aromatic π-conjugated primitives for the design of high-performance lead oxyhalide frameworks. Isophthalates combine the strong electron delocalization typical of organic π-conjugated anions (*e.g.*, cyanurate) with the robust coordination ability characteristic of inorganic primitives (*e.g.*, CO_3_^2−^). Moreover, functionalization at the 5-*meta*-position enables precise control over molecular symmetry and hyperpolarizability. In contrast to the discrete ionic architectures commonly observed in conventional lead halide perovskites, coordination-driven assembly of these *meta*-benzene dicarboxylates with lead halide units promotes uniform alignment and dense packing of asymmetric oxyhalide primitives. Guided by this approach, we have developed an inorganic–organic dual-site synergistic strategy (Scheme 1), affording a new family of 15 highly stable, isoreticular non-centrosymmetric (NCS) lead oxyhalide frameworks. This strategy not only facilitates systematic inorganic modulation of stereoactive [PbX_2_O_6_] polyhedra across the halide series (X = Cl/Br/I), but also directs the ordered alignment of functionalized organic π-conjugated aromatic linkers with varying degrees of polarizability anisotropy and hyperpolarizability. Among them, 12 crystallize in the polar NCS space group *Ima*2 with *mm*2 symmetry and three hydroxyl-substituted derivatives adopt the space group *Cc* with *m* symmetry. These materials possess broad transparency windows, with UV cutoff edges of 298–309 nm for lead chloride and bromide hybrids and 323–337 nm for the iodide analogs. In particular, the lead-iodide framework based on Br-substituted isophthalate exhibits a strong phase-matching SHG response of 11 × KH_2_PO_4_ (KDP) @ 1064 nm and a birefringence of 0.179 @ 546 nm, with both values exceeding the highest performances reported to date for hybrid lead halides. This arises primarily from the synergistic interactions and ordered alignment of inorganic lone-pair and organic π-conjugated primitives. Moreover, the compounds display tunable photoluminescence and efficient long-lived afterglow emission, with the lead halide based on fluoride-substituted isophthalate achieving the highest absolute photoluminescence quantum yield (PLQY) of 93.05% and a phosphorescence lifetime up to 7.36 ms.

## Results and discussion

### Synthesis and structural analysis

**Fig. 1:**
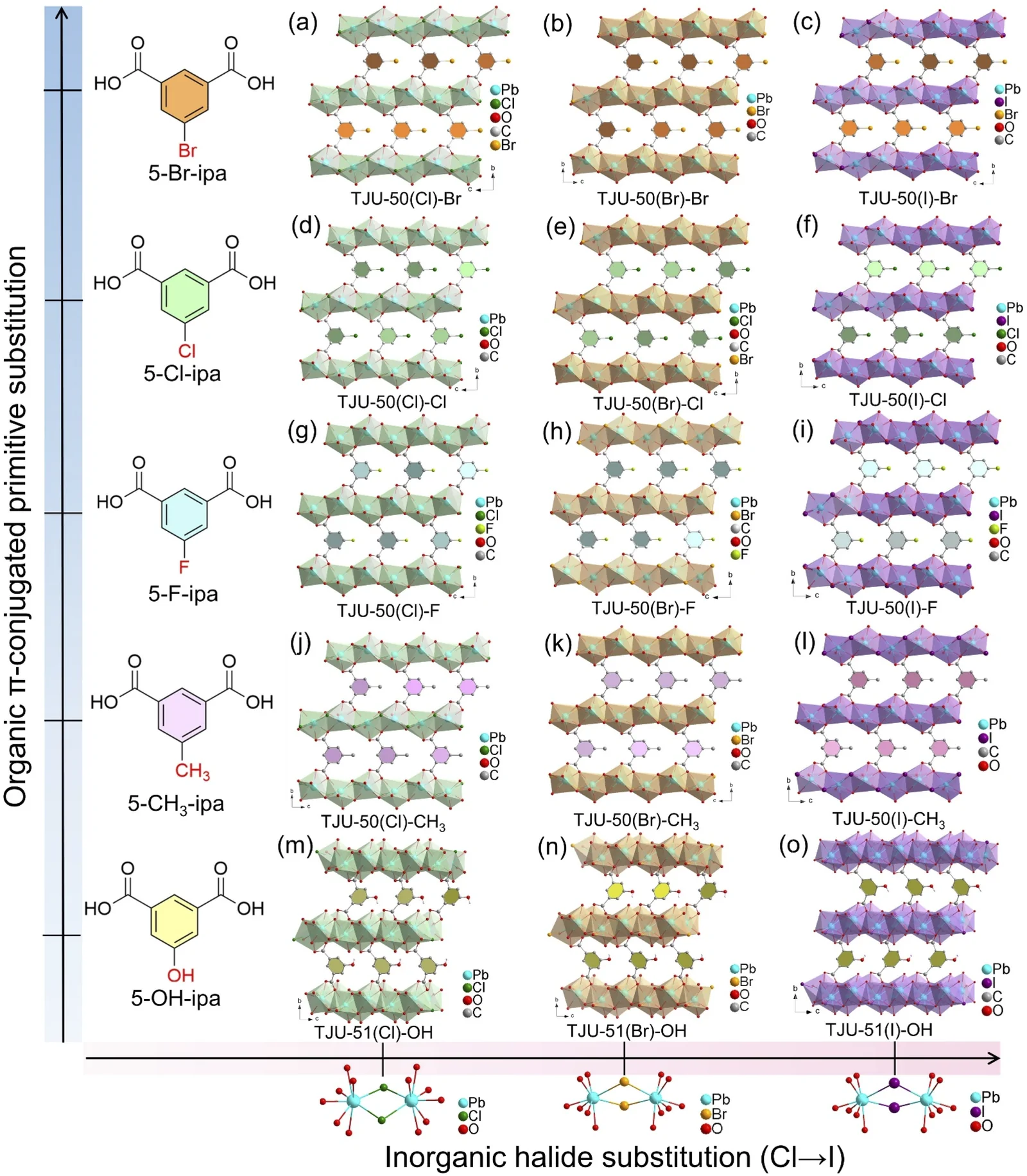
X-ray crystallographic views of the 15 new NCS lead oxyhalide frameworks along the *a*-axis: (a) TJU-50(Cl)–Br, (b) TJU-50(Br)–Br, (c) TJU-50(I)–Br, (d) TJU-50(Cl)–Cl, (e) TJU-50(Br)–Cl, (f) TJU-50(I)–Cl, (g) TJU-50(Cl)–F, (h) TJU-50(Br)–F, (i) TJU-50(I)–F, (j) TJU-50(Cl)–CH_3_, (k) TJU-50(Br)–CH_3_, (l) TJU-50(I)–CH_3_, (m) TJU-51(Cl)–OH, (n) TJU-51(Br)–OH, and (o) TJU-51(I)–OH; the guest cations are omitted for clarity. Pb: sky blue, F: yellowish green, Cl: dark green, Br: orange, I: purple, O: red, C: grey. H atoms are omitted for clarity.

In this work, 15 new isoreticular NCS lead oxyhalide frameworks were synthesized using multiple synthetic approaches, including halide substitution, 5-position substitution of isophthalates, and controlled primitive arrangement (Fig. 1). First, halide substitution was achieved by employing PbCl_2_, PbBr_2_ or PbI_2_ as the inorganic precursors and 5-bromo isophthalate (5-Br-ipa) as the organic component, affording three halide-substituted isostructural [(CH_3_)_2_NH_2_][Pb_2_X(5-Br-ipa)_2_] (denoted as TJU-50(X)–Br, X = Cl^−^/Br^−^/I^−^) (Fig. 1a–c). Taking the iodide structure (TJU-50(I)–Br, Pb_2_I(5-Br-ipa)_2_) as an example, X-ray crystallography at room temperature revealed that it crystallizes in an orthorhombic lattice with the NCS space group *Ima*2 (no. 46), belonging to the *mm*2 point group (Table S1). The atomic coordinates, bond lengths and bond angles are listed in Tables S2–S7. The structure of TJU-50–Br consists of rod-shaped zigzag [Pb_2_X]^3+^ chains propagating along the *c*-axis, which are interconnected by 5-Br-ipa ligands to form the 3D framework (Fig. S1a). The extra framework (CH_3_)_2_NH_2_^+^ cations are anchored within the 1D void spaces *via* N–H⋯O hydrogen bonds to the carboxylate oxygen atoms of the ipa ligands, which accounts for their ordered arrangement (Fig. S1b). The asymmetric inorganic unit of TJU-50 comprises one crystallographically independent Pb^2+^ center coordinated by six oxygen atoms and two iodine atoms (Fig. S1c). The lone-pair electrons of stereoactive Pb^2+^ centers are directed toward the Pb-(*µ*_2_)I–Pb connectivity, which affords highly distorted [PbX_2_O_6_] units. The magnitude of the polyhedral distortions (Δ*d*) in [PbX_2_O_6_] was quantified by deviations in Pb–O and Pb–I bond lengths, calculated using eqn (1):1
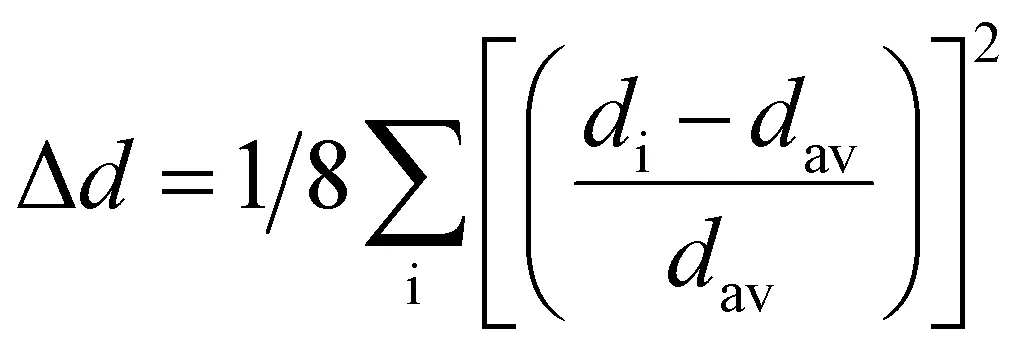
where *d*_i_ represents individual Pb–O/Pb–I bond lengths and *d*_av_ is their average (Table S4). For TJU-50(I)–Br, the calculated Δ*d* is 2.57 × 10^−3^, which is two orders of magnitude higher than that of conventional lead halides with idealized [PbX_6_] octahedra,^48^ typically in the range of 10^−5^.

On the organic side, the modulation of π-conjugated organic primitives was achieved using five different *meta*-substituted ipa components: 5-fluoro, 5-chloro, 5-bromo, 5-methyl, and 5-hydroxyl-isophthalates (5-F-ipa, 5-Cl-ipa, 5-Br-ipa, 5-CH_3_-ipa, and 5-OH-ipa). Notably, this study represents the first systematic investigation into the diverse functional substituents of aromatic dicarboxylate primitives, despite prior investigations on halogen substitution.^45^ In addition to the three TJU-50(X)–Br frameworks, halide-substituted ipa derivatives (5-F-ipa and 5-Cl-ipa) were used for the synthesis of six other isostructural frameworks, including three [(CH_3_)_2_NH_2_][Pb_2_X(5-F-ipa)_2_] (TJU-50(X)–F, X = Cl^−^/Br^−^/I^−^) (Fig. 1g–i and Tables S15–S21) and three [(CH_3_)_2_NH_2_][Pb_2_X(5-Cl-ipa)_2_] (TJU-50(X)–Cl, X = Cl^−^/Br^−^/I^−^) (Fig. 1d–f and Tables S8–S14). Taking TJU-50(X)–Br as the example, macroscopic symmetry analysis reveals that the Br substituents in the organic ligands occupy two distinct chemical environments. Specifically, Br(1) is positioned on the mirror plane (1/2 − *x*, *y*, *z*), while Br(2) resides at a site governed by multiple symmetry elements, including a two-fold screw axis (1/2 − *x*, 1/2 − *y*, 1/2 + *z*) and the interface of two glide planes (Fig. S2a). This suggests that the 5-substituted halogen atoms (–F, –Cl, and –Br) in the ipa primitives play a crucial role in modulating dipole moments and molecular symmetry. Moreover, replacing *meta*-halogen substituents with methyl groups, which possess three-fold rotation symmetry, resulted in three isostructural [(CH_3_)_2_NH_2_][Pb_2_X(5-CH_3_-ipa)_2_] (TJU-50(X)–CH_3_, X = Cl^−^/Br^−^/I^−^) in the same NCS space group, *Ima*2 (Fig. 1j–l and Tables S22–S28). In addition, the hydrogen atoms of the methyl group are highly disordered in the single-crystal structure, which corresponds to their location on symmetry elements (Fig. S2b). Furthermore, in these structures, the coordinating carboxylate groups of the ipa derivatives primarily contribute to the local dipole moments of the organic primitives, as they are uniformly aligned with both carboxylate ends oriented towards the *c*-axis (Fig. S3a). This structural alignment directly correlates with the anisotropic lattice expansion observed upon 5-functionalization: while the *a* and *b* axes expand to a limited extent with increasing substituent size, the *c*-axis undergoes a pronounced expansion (Fig. S3b, Tables S1, S7, S13 and S19). Due to the opposite positioning of the *meta*-substituted functional groups relative to the carboxylates, the presence of strong electron-withdrawing groups at the *meta*-position leads to partial cancellation of the dipole moment of the ipa-based primitives. As electron affinity of the *meta*-substituted functional groups decreases in the order of –CH_3_ > –F > –Cl > –Br, the overall polarization of the organic primitives correspondingly increases. Moreover, first-principles calculations reveal that the four organic π-conjugated primitives in TJU-50 efficiently regulate the polarizability anisotropy and hyperpolarizability increasing in the order 5-F-ipa < 5-CH_3_-ipa < 5-Cl-ipa < 5-Br-ipa, enabling the precise tuning of the SHG response and birefringence (Fig. S4).

Beyond tuning the halide species and incorporating π-conjugated organic primitives, we further investigate the structural primitive arrangement to extend the structural types for this family of lead oxyhalide frameworks. Notably, the introduction of hydroxyl substituents (–OH) in 5-OH-ipa resulted in the formation of three new frameworks, [(CH_3_)_2_NH_2_][Pb_2_X(5-OH-ipa)_2_] (TJU-51(X)–OH, X = Cl^−^/Br^−^/I^−^) (Fig. 1m–o), which crystallize in a monoclinic lattice with a distinct NCS space group *Cc* (no. 9) and point group *m* (Tables S29–S35). Given the structural similarity between TJU-50 (with –F/Cl/Br/CH_3_ groups) and TJU-51–OH (with the –OH group) despite their crystallization in different space groups, a detailed structural comparison was performed to investigate their primitive arrangements. First, all twelve TJU-50 structures, crystallizing in the *Ima*2 space group, exhibit more symmetry elements, including a 2-fold rotation axis (−*x*, −*y*, +*z*), a mirror plane (1/2 − *x*, *y*, *z*), a 2-fold screw axis (1/2 − *x*, 1/2 −*y*, 1/2 + *z*) and three glide planes along the screw axis (1/2 + *x*, −*y*, *z*), (−*x*, 1/2 + *y*, 1/2 + *z*), and (*x*, 1/2 − *y*, 1/2 + *z*) (Fig. S5a). These symmetry elements promote the uniform alignment of structural primitives, thereby enhancing the partial dipole moment under similar polar NCS structural conditions. For example, in TJU-50(I)–Br, the ordered superposition of structural units results in a crystallographically independent Pb^2+^ center and a single 5-Br-ipa molecule, which are oriented along the (010) plane and the (100) plane. These lattice planes are perpendicular to the mirror plane and the glide plane, respectively, with all *meta*-position functional groups (F, Cl, Br, and CH_3_) aligned along the *c*-axis. In contrast, TJU-51–OH only contains two glide planes, (1/2 + *x*, 1/2 − *y*, 1/2 + *z*) and (*x*, −*y*, 1/2 + *z*), resulting in a lower symmetry (Fig. S5b). This configuration introduces two crystallographically independent Pb^2+^ centers and two 5-OH-ipa ligands, which lie along the (100) plane and are perpendicular to the glide plane. Second, the arrangement of π-conjugated organic primitives in TJU-50 exhibits parallel stacking, which facilitates π-conjugation and maximizes the dipole moments. The minimal dihedral angle of 11.38° observed between neighboring phenyl rings in ipa derivatives along the (010) plane suggests efficient π-conjugation (Fig. S6a). In contrast, the 5-OH-ipa primitives in TJU-51(I)–OH adopt a staggered fashion, with a larger dihedral angle of 16.19°, which suppresses face-to-face π-conjugation (Fig. S6b). Third, the dipole moments of the [PbI_2_O_6_] units within the unit cells of TJU-50(I)–Br and TJU-51(I)–OH were calculated using the bond-valence method (Tables S36 and S37). While the individual dipole moments of [PbI_2_O_6_] remain comparable among these compounds (ranging from 0.71 to 4.61 D), in TJU-50(I)–Br, the calculated *x*- and *y*- components of polarization in the [PbI_2_O_6_] units cancel each other, while the *z*-components accumulate to afford a large net dipole moment of 5.04 D along the *c*-axis. The uniformly aligned, stereoactive [PbI_2_O_6_] polyhedra along the *c*-axis establish a polar direction, resulting in an additive arrangement of 1D inorganic chains that enhances the overall polarization. In contrast, in TJU-51(I)–OH, the net dipole moment of the individual [PbI_2_O_6_] units is reduced to 3.05 D along the *c*-axis, reflecting the irregular orientation of the [PbI_2_O_6_] units that results in partial cancellation of the dipole moments. Therefore, although TJU-51(I)–OH adopts a lower-symmetry *Cc* space group, the non-uniform arrangement of the inorganic and organic primitives prevents the oriented superposition of the dipoles, resulting in partial cancellation of the dipole moments.

All fifteen members of TJU-50 and TJU-51 were synthesized with high yields and demonstrate excellent phase purity, as confirmed by the good agreement between experimental PXRD patterns and the simulated patterns from single-crystal data (Fig. S7). It is worth noting that the as-synthesized TJU-50 and TJU-51 materials exhibit high structural stability, even after exposure to various polar organic solvents, including MeOH, EtOH, dichloromethane, acetonitrile, acetone and DMF, at room temperature. All twelve TJU-50 materials maintained high crystallinity and showed no obvious weight loss after 24 hours of incubation under aqueous conditions across a wide pH range (5–12) (Fig. S8–S11). However, it was observed that the three TJU-51 materials were degraded upon incubation under aqueous conditions, probably owing to hydrogen bonding interactions between the framework hydroxyl groups and H_2_O solvent. The materials also exhibited remarkable photostability, withstanding continuous light irradiation (300 W Xe lamp, 96 W cm^−2^) for 24 hours at 90% relative humidity (RH) and room temperature (Fig. S12). Thermogravimetric analysis (TGA) and *in situ* thermal PXRD diffraction further demonstrated their high thermal stability, with decomposition temperatures around 510 K for both TJU-50 and TJU-51 (Fig. S13 and S14). The excellent stability is attributed to the formation of coordination networks, which offer greater structural integrity compared to the ionic bonding found in conventional hybrid lead halides.^49^ Across this family of lead oxyhalide frameworks featuring organic ligands with different substituents, the CBMs remain largely governed by C 2p and O 2p orbitals of the isophthalate ligands, with a smaller contribution from Pb 6p orbitals. The VBMs are mainly composed of Pb 6s and I 5p orbitals, accompanied by additional halogen p-orbital contributions from F 2p in TJU-50(I)–F, Cl 3p in TJU-50(I)–Cl, and Br 4p in TJU-50(I)–Br, respectively (Fig. S15). The experimental UV cutoff edges were observed at 328 nm for TJU-50(I)–F, 327 nm for TJU-50(I)–Cl, 323 nm for TJU-50(I)–CH_3_, and 330 nm for TJU-51(I)–OH, corresponding to the optical bandgaps of 3.78 eV, 3.79 eV, 3.84 eV and 3.76 eV, respectively (Fig. S16c, S17c, S18c and S19c). However, a comparison of the bandgap variations reveals that a more pronounced redshift from 3.70 eV to 4.05 eV occurs with heavier halogens in the [PbX_2_O_6_] units based on the identical 5-Br-ipa ligand, whereas varying the substituents (–F, –Cl, –Br, –CH_3_, and –OH) on the organic ligands results in only minor bandgap changes from 3.76 eV to 3.84 eV, indicating the significant effect of halogens in inorganic units on the bandgaps of the materials.

### Optical properties

#### Bandgaps

**Fig. 2:**
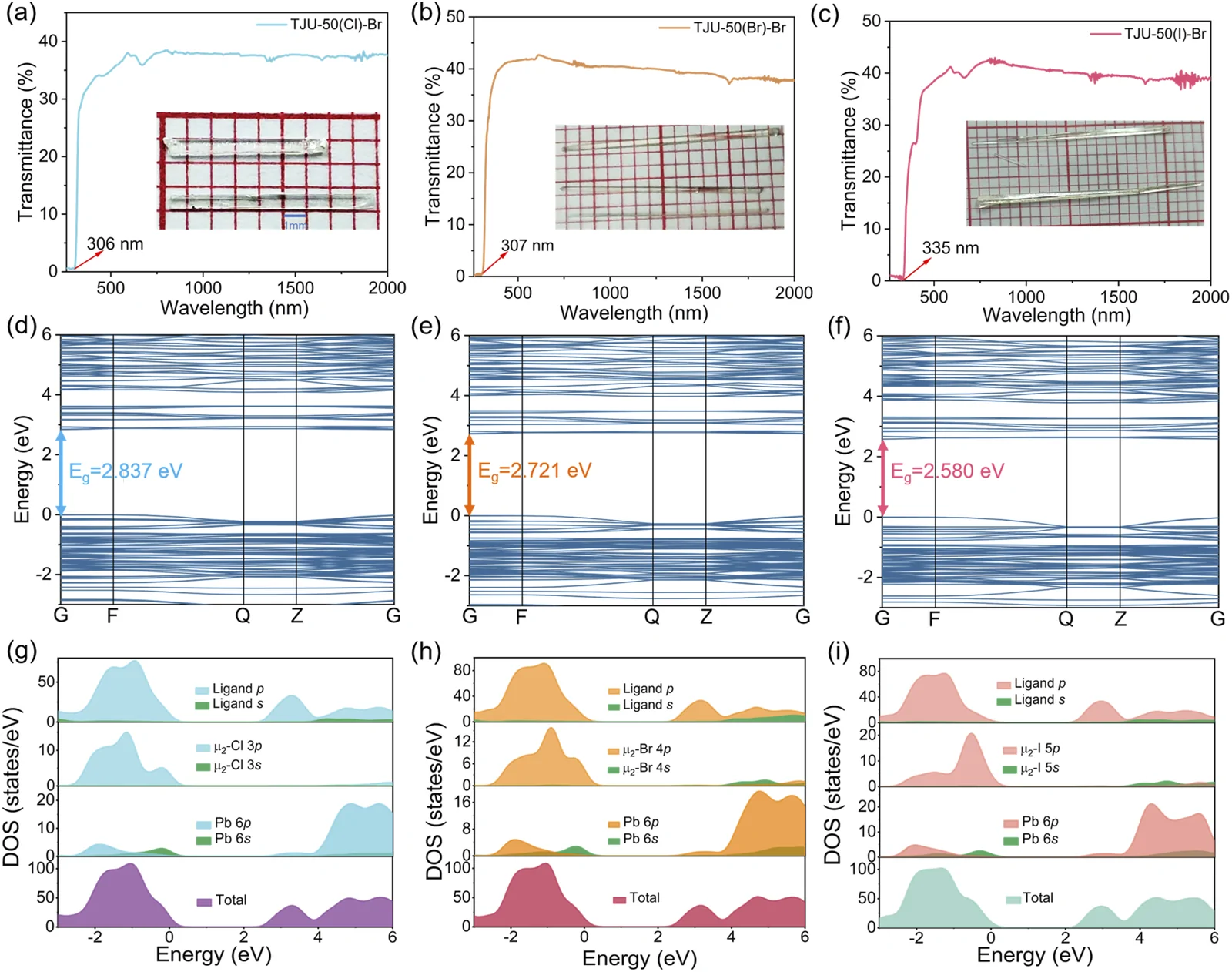
(a–c) UV-vis-NIR transmittance spectra of TJU-50(Cl)–Br (a), TJU-50(Br)–Br (b), and TJU-50(I)–Br (c). (d–f) Calculated band structures of TJU-50(Cl)–Br (d), TJU-50(Br)–Br (e), and TJU-50(I)–Br (f). (g–i) The projected density of states of TJU-50(Cl)–Br (g), TJU-50(Br)–Br (h), and TJU-50(I)–Br (i).

In addition to the distortion in the [PbX_2_O_6_] units, halide substitution serves as an effective approach for bandgap engineering, which is a well-established approach for tuning the magnitude of NLO responses.^50,51^ UV-vis-NIR transmission spectra of the three TJU-50–Br materials were measured using as-grown, prism-shaped crystals with maximum dimensions of 15 × 0.2 × 0.05 mm^3^ (Fig. 2a–c). The UV cutoff edges were observed at 304 nm for TJU-50(Cl)–Br, 305 nm for TJU-50(Br)–Br, and 335 nm for TJU-50(I)–Br, corresponding to the optical bandgaps of 4.08 eV, 4.07 eV, and 3.70 eV, respectively (Fig. 2a–c). The redshift in the bandgap with heavier halogens clearly demonstrates that the valence bands in this family of NCS lead oxyhalide frameworks are predominantly derived from the p-orbitals of the halogen atoms, consistent with trends observed in conventional lead halide perovskites.^52,53^ Notably, these bandgaps are significantly larger than those reported for other lead-based NLO compounds, such as Pb_3_SeO_5_ (3.30 eV),^54^ CdPb_8_(SeO_3_)_4_Br_10_ (3.32 eV),^55^ Pb_2_Bi(SeO_3_)_2_Cl_3_ (3.45 eV)^56^ and Pb_3_(SeO_3_)Br_4_ (3.35 eV).^55^ This enhancement is primarily attributed to the presence of π-conjugated aromatic carboxylate primitives in our lead oxyhalide frameworks.^17^ The estimated transmission ranges for TJU-50(Cl)–Br and TJU-50(Br)–Br span from 300 nm to 2000 nm, covering the near-ultraviolet, visible, and infrared regions, highlighting their strong potential as promising optical functional materials. We have also performed band structure and the densities of states (DOS) calculations on the three TJU-50–Br frameworks. The calculated band structures based on the Perdew–Burke–Ernzerhof functional show that these lead oxyhalide frameworks have direct bandgaps with both the valence band (VB) maximum and the conduction band (CB) minimum located at the G1 point. The calculated bandgaps are 2.837 eV, 2.721 eV, and 2.580 eV for TJU-50(Cl)–Br, TJU-50(Br)–Br, and TJU-50(I)–Br, respectively (Fig. 2d–f). Due to the discontinuity of the exchange–correlation energy, the DFT-calculated bandgaps are consistently smaller than the experimentally observed values.^57^ The density of states (DOS) and partial density of states (PDOS) diagrams of these compounds are shown in Fig. 2g–i. The VBMs have predominant contributions from C 2p, O 2p, and halogen *n*p (Cl 3p, Br 4p, or I 5p) orbitals, with a minor contribution from Pb 6s orbitals, while the CBMs are mainly determined by C 2p and O 2p orbitals from 5-Br-ipa. Notably, the halogen variation within the [PbX_2_O_6_] building units significantly influences the bandgap, corresponding to redshift of measured bandgaps. The relatively higher energy of the I 5p orbitals results in an upward shift of the top of the VB and a decreased calculated bandgap for TJU-50(I)–Br compared to TJU-50(Cl)–Br and TJU-50(Br)–Br.

#### SHG responses

**Fig. 3:**
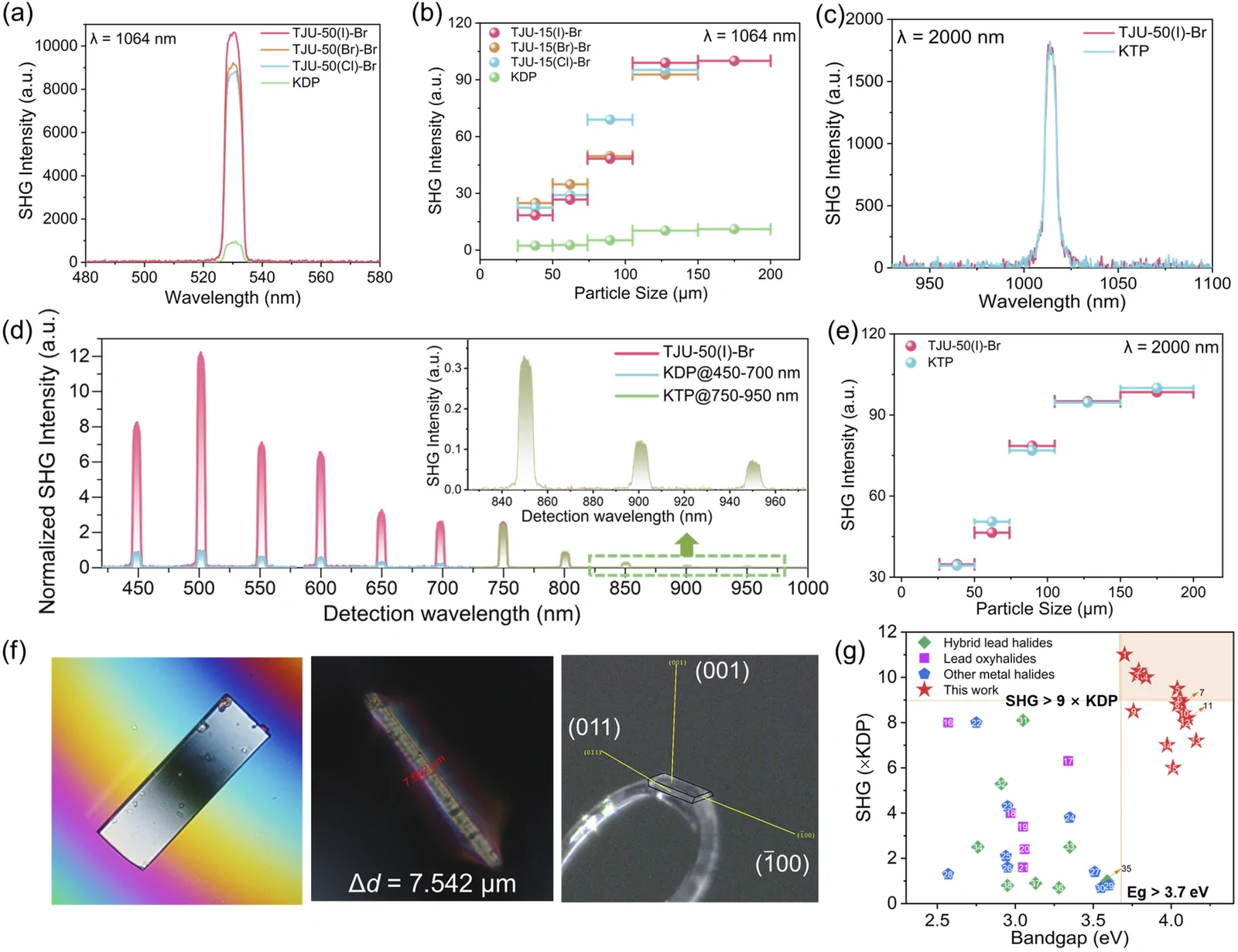
Optical measurements of TJU-50–Br lead halide frameworks. (a) Oscilloscope traces of the SHG signals for powders of TJU-50(Cl)–Br, TJU-50(Br)–Br and TJU-50(I)–Br (150–200 µm) with 1064 nm laser radiation. KDP was used as a reference for the SHG measurements at 1064 nm. (b) Phase-matching curves of TJU-50(Cl)–Br, TJU-50(Br)–Br and TJU-50(I)–Br and KDP with 1064 nm laser radiation. (c) Oscilloscope traces of the SHG signals for powders of TJU-50(I)–Br with 2000 nm laser radiation. KTP was used as a reference for the SHG measurements at 2000 nm. (d) Normalized SHG signals of TJU-50(I)–Br and benchmark KDP and KTP for particle size 150–200 µm. KDP and KTP samples serve as the references for the SHG measurements in the ranges of 900–1400 nm and 1500–1900 nm. (e) Phase-matching curves of TJU-50(I)–Br with 2000 nm laser irradiation. (f) (001) crystal plate of TJU-50(I)–Br achieving extinction, and corresponding thickness of the (001) crystal plate for birefringence measurements. Crystal orientation of the TJU-50(I)–Br crystal as indexed by single-crystal X-ray diffraction. (g) Bandgap and SHG response distribution for selected halide materials: Pb(ii)-based oxyhalides, Pb(ii)-based hybrid halides, and Ge(ii)/Sb(iii)-based hybrid halides (see Table S38 for details).

Powder SHG measurements were performed to investigate the effects of isoreticular functionalization-dependent structural polarity on the NLO properties. Crystals of TJU-50(Cl/Br/I)–Br with particle sizes ranging from 150 to 200 µm exhibited characteristic SHG responses at 1064 nm, with values of 9.0, 9.5, and 11.0 × KDP, respectively (Fig. 3a). In addition, the SHG responses of TJU-50(Cl/Br/I)–F, TJU-50(Cl/Br/I)–Cl, TJU-50(Cl/Br/I)–CH_3_ and TJU-51(Cl/Br/I)–OH were measured as follows: 8.2, 8.8 and 10.1 × KDP for TJU-50(Cl/Br/I)–F, 8.4, 8.9, and 10.3 × KDP for TJU-50(Cl/Br/I)–Cl, 7.2, 8.0, and 10.0 × KDP for TJU-50(Cl/Br/I)–CH_3,_ and 6.0, 7.0 and 8.5 × KDP for TJU-51(Cl/Br/I)–OH (Fig. S20–S23). It is well-known that the SHG contributions of halide anions typically follow the trend I > Br > Cl, correlating with the changes in the bandgaps of hybrid lead halides.^52,53^ For all TJU-50 and TJU-51 compounds, the SHG intensities increased with the particle size until reaching a maximum, independent of the particle size at 1064 nm and consistent with phase-matchable behavior (Fig. 3b and S20–S23). Notably, TJU-50(I)–Br displays a strong SHG response, measured to be 1.0 × KTiOPO_4_ (KTP) under 2000 nm laser irradiation in the particle size range of 150–200 µm (Fig. 3c). The SHG signal was confirmed to be phase-matchable with the 2000 nm fundamental radiation (Fig. 3e). It is worth noting that the SHG response of TJU-50(I)–Br is the largest value reported to date among all of the lead halide-based compounds (Table S38). The powder SHG intensity of TJU-50(I)–Br was further studied using incident laser wavelengths ranging from 900 to 1900 nm (Fig. 3d). Within the same particle size range, TJU-50(I)–Br exhibited strong SHG intensities of 8.4–14.5 × KDP in the 900–1400 nm range, and 0.9–1.1 × KTP in the 1500–1900 nm. These variations in SHG intensities are attributed to the intrinsic wavelength dependence of the frequency multiplication efficiency.^58^ In addition, the SHG performance of TJU-50(I)–Br surpasses that of many high-performance broadband Pb^2+^-based NLO materials, including Pb^2+^-based oxyhalides, as well as other typical metal-based (Ge^2+^/Sb^3+^) halides (Fig. 3g and Table S38).

#### Birefringence properties

The optical anisotropy of TJU-50 and TJU-51 was analyzed using polarizing microscopy and first-principles calculations. Birefringence measurements were conducted at a wavelength of 546 nm using a ZEISS AXIO Scope.A1 polarizing microscope. As shown in Fig. 3f, S24a and b, the crystals of TJU-50(Cl)–Br, TJU-50(Br)–Br and TJU-50(I)–Br exhibit complete extinction by the positive and negative rotation of the compensator. The measured optical path differences for the three samples were 0.90 µm for TJU-50(Cl)–Br, 0.94 µm for TJU-50(Br)–Br, and 1.35 µm for TJU-50(I)–Br, corresponding to crystal thicknesses of 6.841, 6.445, and 7.542 µm, respectively. Single-crystal XRD analyses identified the measured crystals as the (001̄), (001), and (001) crystal planes for TJU-50(Cl)–Br, TJU-50(Br)–Br, and TJU-50(I)–Br, respectively. Based on the formula *R* = |*n*_e_ − *n*_o_| × *d* = Δ*n* × *d*, the refractive index differences were calculated to be Δ*n*(001) = 0.132 for TJU-50(Cl)–Br, Δ*n*(001) = 0.145 for TJU-50(Br)–Br, and Δ*n*(001) = 0.179 for TJU-50(I)–Br at 546 nm. The refractive index difference on a specific crystal plane of other crystals of the series was also determined at 546 nm: 0.089, 0.096 and 0.118 for TJU-50(Cl/Br/I)–F; 0.115, 0.126 and 0.137 for TJU-50(Cl/Br/I)–Cl; 0.076, 0.097 and 0.116 for TJU-50(Cl/Br/I)–CH_3_; 0.056, 0.107 and 0.112 for TJU-51(Cl/Br/I)–OH (Fig. S25). These birefringence values are sufficient to enable phase matching at the standard laser wavelength of 1064 nm. Several observations can be made from the measured birefringence: (i) for frameworks incorporating the same ipa ligands, birefringence increases with the heavier halogens; and (ii) for structures with identical lead halide motifs but different ipa ligands, birefringence variations positively correlate with the SHG response. The refractive index curves were calculated in Fig. S26 and S27; the derived calculated birefringence at 546 nm is in the range of 0.330–0.359 for TJU-50(Cl/Br/I)–Br and 0.215–0.327 for TJU-50(I)–Br, TJU-50(I)–CH_3_, TJU-50(I)–F, TJU-50(I)–C and TJU-51(I)–OH, respectively. The calculated values reveal that the halogen species in the inorganic [PbX_2_O_6_] polyhedra have negligible influence on the birefringence across the TJU-50–Br series. In contrast, the *meta*-functional groups on the ipa ligands significantly modulate the birefringence of the TJU-50(I)–CH_3_, TJU-50(I)–F, TJU-50(I)–Cl, TJU-50(I)–Br and TJU-51(I)–OH, particularly in structures constructed from the 5-Br/Cl-ipa ligands. The polarization anisotropy of the functionalized linkers increases in the order 5-F-ipa < 5-OH-ipa < 5-CH_3_-ipa < 5-Cl-ipa < 5-Br-ipa, identifying 5-Br-ipa as the optimal ligand for maximizing birefringence (Fig. S4). A summary of the SHG response, birefringence, UV cutoff edges and optical bandgaps for all 15 isoreticular NCS lead oxyhalide frameworks is provided in Table 1.

**Table 1:** The summary of SHG responses, birefringence, UV cutoff edges and optical bandgaps of the 15 new isoreticular NCS lead oxyhalide frameworks

Compounds	SHG (×KDP)	Δ*n* @ 546 nm	UV cut-off edges (nm)	Bandgaps (eV)
TJU-50(Cl)–Br	9.0	0.132	306	4.052
TJU-50(Br)–Br	9.5	0.145	307	4.039
TJU-50(I)–Br	11.0	0.179	335	3.701
TJU-50(Cl)–Cl	8.4	0.115	304	4.079
TJU-50(Br)–Cl	8.9	0.126	305	4.066
TJU-50(I)–Cl	10.3	0.137	327	3.792
TJU-50(Cl)–F	8.2	0.089	302	4.106
TJU-50(Br)–F	8.8	0.096	307	4.039
TJU-50(I)–F	10.1	0.118	328	3.781
TJU-50(Cl)–CH_3_	7.2	0.076	298	4.160
TJU-50(Br)–CH_3_	8.0	0.097	303	4.090
TJU-50(I)–CH_3_	10.0	0.116	323	3.839
TJU-51(Cl)–OH	6.0	0.056	309	4.012
TJU-51(Br)–OH	7.0	0.107	312	3.974
TJU-51(I)–OH	8.5	0.112	330	3.758

#### Theoretical calculations

**Fig. 4:**
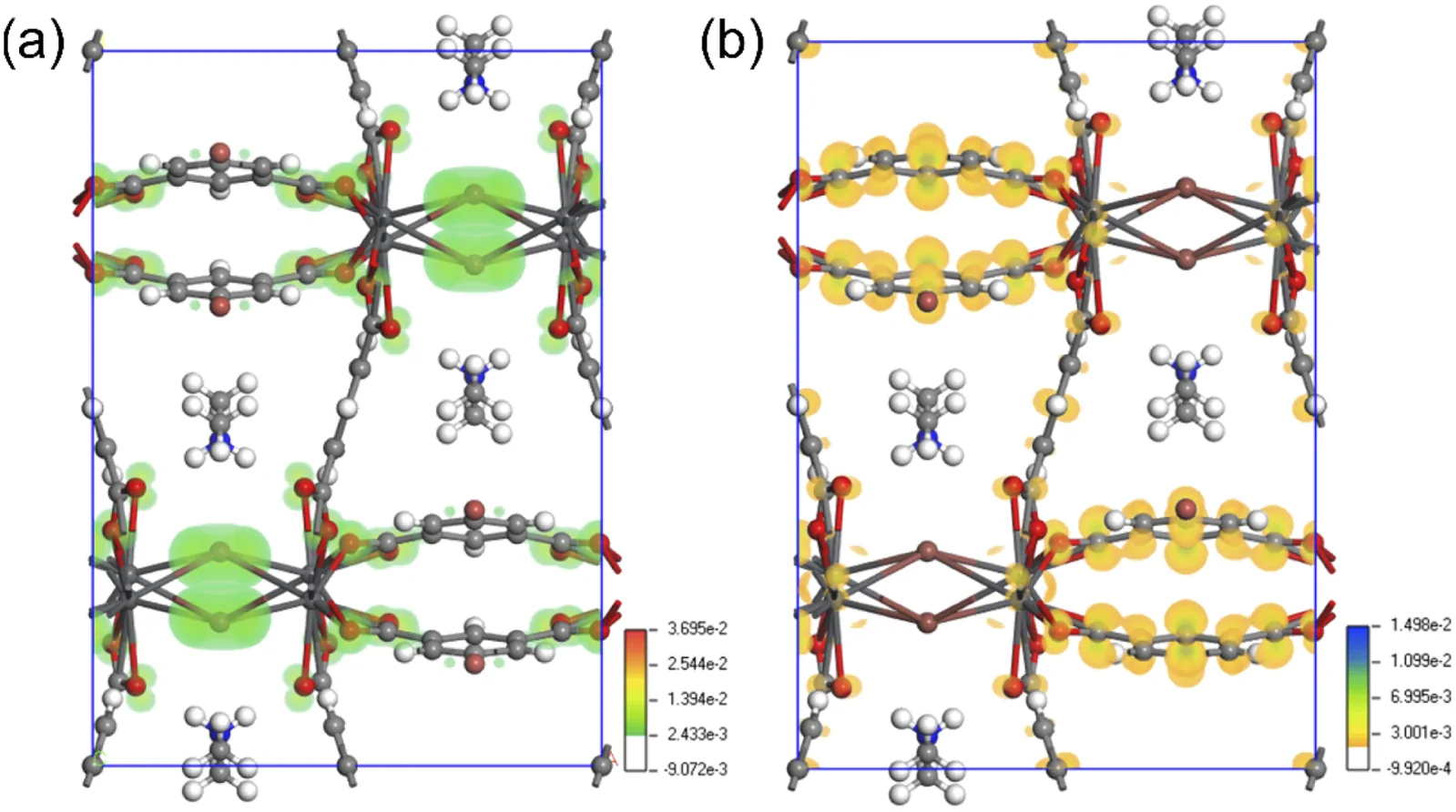
SHG-weighted density for (a) occupied and (b) unoccupied states in the virtual electron process for TJU-50(I)–Br.

The relationship between the lead oxyhalide frameworks derived from our coordination-driven assembly approach and the linear and nonlinear optical properties can be more fully understood based on theoretical studies. SHG-weighted electron density analyses were performed to elucidate the contributions of the individual components of lead oxyhalide frameworks to the SHG effect in real space (Fig. 4). The occupied and unoccupied states in the virtual electron (VE) process make the predominant contribution to the overall SHG effect, so particular attention was paid to these states in the SHG-weighted electron density analyses of lead oxyhalide frameworks, with the results showing that the occupied states are mainly contributed by the inorganic [PbX_2_O_6_] building units, while the organic ligands make the major contributions to the unoccupied states (Fig. 4a and b). To clarify the widely divergent SHG responses for these lead oxyhalide frameworks that result from the inorganic–organic dual-site strategy, theoretical studies on the SHG coefficients were performed. Under the restriction of Kleinman symmetry, TJU-50 have three nonzero independent SHG coefficients (*d*_15_, *d*_24_, and *d*_31_) while TJU-51 have six nonzero SHG coefficients (*d*_11_, *d*_15_, *d*_12_, *d*_13_, *d*_24_, and *d*_33_). The calculated *d*_eff_ values of powder crystals of TJU-50(Cl)–Br, TJU-50(Br)–Br, TJU-50(I)–Br, TJU-50(I)–F, TJU-50(I)–Cl, TJU-50(I)–CH_3_, and TJU-51(I)–OH are 1.29, 0.96, 0.92, 1.46, 1.36, 1.43, and 1.80 pm V^−1^ under 1064 nm irradiation, respectively, based on the formula^59,60^*d*_eff_ = *d*_eff_(KDP) × (*I*_2*ω*_/*I*_2*ω*_(KDP))^1/2^. Real-space atom-cutting analyses further quantified contributions to the *d*_15_ SHG coefficient of TJU-50, as exemplified by TJU-50(I)–Br: the [PbI_2_O_6_] units account for 33%, while the 5-Br-ipa contributes 58% (Table S39); in contrast, the contributions of the guest cations [(CH_3_)_2_NH_2_]^+^ are rather minimal (∼9%). The largest SHG coefficient *d*_15_ for TJU-51(I)–OH derives predominantly from the 5-OH-ipa units, which is confirmed by the calculated percentages of SHG contributions from [PbI_2_O_6_] (28%), 5-OH-ipa (59%) and [(CH_3_)_2_NH_2_]^+^ (13%) (Table S40). Further analysis of first-order hyperpolarizabilities revealed that 5-Br-ipa possesses the highest value among *meta*-substituted ipa ligands (Fig. S4). This implies that under the perturbation of ambient optoelectronic fields, the SHG contribution of the organic ligands is larger than that of the inorganic [PbX_2_O_6_] building units, with the largest SHG response being seen with TJU-50(I)–Br, attributed to its significantly large first-order hyperpolarizability.

#### Room-temperature phosphorescence

The ipa and its derivative molecules are known for their distinct configuration, with a well-defined 120° angle between two carboxylates, and their versatile coordination modes. These structural characteristics often lead to diverse RTP properties arising from the n → π* transition of the carboxylic group. However, the relationship between the RTP properties of this class of electron acceptors and their various functionalizations, as well as their synergistic effects with light-harvesting lead halide units, remains largely unexplored. The presence of electron-withdrawing groups at the *meta*-position in ipa derivatives may introduce strong halogen bonding, tune their electron density, and enhance RTP performance. Our coordination-driven assembly of isoreticular lead oxyhalide frameworks incorporates light-absorbing lead halide units and different functionalized ipa ligands, providing an ideal platform to modulate RTP behaviors. Moreover, the high crystallinity, coordination network and strong intermolecular interactions of ipa (*e.g.* π–π stacking, hydrogen bonding and halogen bonding) in TJU-50 and TJU-51 provide a rigid environment that reduces nonradiative decay of triplet excitons.

**Fig. 5:**
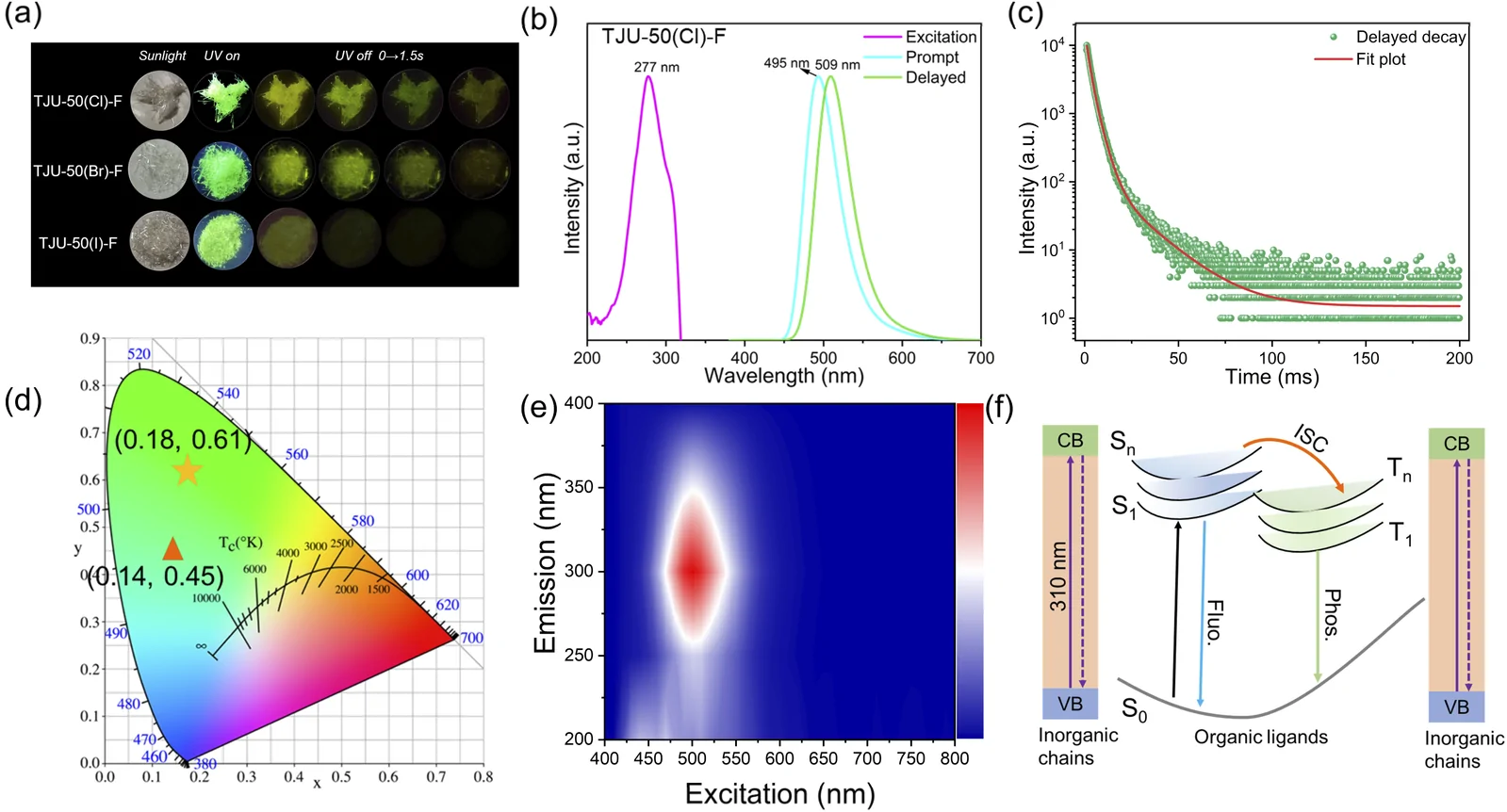
Photoluminescence (PL) characterization of TJU-50(Cl)–F. (a) Photographs of the luminescent crystals before and after cessation of the 310 nm irradiation. (b) Excited, prompt and delayed PL spectra. (c) Time-resolved decay curve of the delayed PL at 300 K. (d) CIE coordinates of the prompt PL emission (orange triangle) and green afterglow (yellow star). (e) Contour plot of the 3D PL map in prompt-mode. (f) Schematic illustration of the proposed photophysical processes.

The photophysical properties of TJU-50–F/Cl/Br/CH_3_ and TJU-51–OH crystals were first studied using steady-state and transient-state PL spectroscopy under ambient conditions. When irradiated with 310 nm UV light, the colorless as-synthesized crystals of TJU-50(X)–F emit green light visible to the naked eye (Fig. 5a). An immediate disappearance of green emission upon removal of the UV excitation is observed, but a concomitant presence of a pale yellow-green afterglow remains visible for ∼1.5 s. As shown in Fig. 5b and S28, the steady-state prompt PL spectra of TJU-50(Cl/Br/I)–F exhibit green emission centered at ∼495 nm under the maximum excitation at 277 nm. The prompt three-dimensional (3D) PL excitation–emission correlation maps confirm the strongest emission band at ∼499 nm, showing a single green emission center in the 470–525 nm range (Fig. 5e and S29). Based on the emission spectra, the CIE chromaticity coordinates are calculated to be (0.16, 0.44) for TJU-50(Cl)–F, (0.16, 0.41) for TJU-50(Br)–F and (0.17, 0.44) for TJU-50(I)–F, respectively (Fig. 5d and S30). In the delay-mode PL spectra under 310 nm excitation (with a delay of 0.1 s), the emission profiles are normally distributed and exhibit the prominent long-lived green afterglow with the maximum emission at 509 nm for TJU-50(Cl)–F, 506 nm for TJU-50(Br)–F, and 508 nm for TJU-50(I)–F, respectively (Fig. 5b and S28).^61^ The calculated CIE coordinates for delayed-mode emission are (0.17, 0.45) for TJU-50(Cl)–F, (0.15, 0.40) for TJU-50(Br)–F and (0.16, 0.42) for TJU-50(I)–F, respectively (Fig. 5d and S30). Importantly, the PLQYs of strong green prompt PL reach 67.26% for TJU-50(Cl)–F, 93.05% for TJU-50(Br)–F and 20.19% for TJU-50(I)–F, surpassing those of most reported hybrid lead halides (Fig. S31).^62–64^ Moreover, the afterglow quantum yields are measured to be 14.05% for TJU-50(Cl)–F, 33.37% for TJU-50(Br)–F and 11.34% for TJU-50(I)–F. Meanwhile, the Cl/Br/CH_3_-functionalized TJU-50 shows relatively low PLQYs and no RTP emission across all UV-excitation wavelengths. This is ascribed to the lower intrinsic PL efficiency of the 5-Cl/Br/CH_3_-ipa ligands, which limits the population of initial S_1_ excitons to drive the ISC process, as will be discussed in detail later. In addition, the PLQYs of all three TJU-51–OH frameworks are below 1%, probably owing to the absence of π–π stacking induced by the staggered arrangement of 5-OH-ipa molecules.

To investigate the origin of the green afterglow in TJU-50(Cl/Br/I)–F, we first synthesized Na_2_(5-F-ipa), which is a typical π-type halogen-bonded linker obtained by ipa functionalization. Na_2_(5-F-ipa) shows a prompt blue emission with the maxima at 451 nm and a green afterglow at 495 nm, closely matching the emission characteristics of the as-synthesized TJU-50–F materials (Fig. S32). This suggests that the prompt PL and phosphorescence of TJU-50–F primarily originate from the 5-F-ipa component. Based on the lifetime decay profiles, the emission at 451 nm in Na_2_(5-F-ipa) is identified as prompt PL with a short lifetime of 2.97 ns, while the green emission at 499 nm corresponds to long-lived afterglow with a lifetime of 10.63 ms (Fig. S33). However, Na_2_(5-F-ipa) exhibits a low PLQY of 5.5% for the prompt emission (Fig. S34). Time-resolved PL decay measurements for TJU-50(Cl)–F reveal average decay lifetimes for the short-lived and long-lived excited states of 2.18 ns and 7.36 ms, respectively (Fig. 5c and S35). For the bromide and iodide analogues, the corresponding lifetimes are 2.09 ns and 6.22 ms for TJU-50(Br)–F, and 2.03 ns and 4.93 ms for TJU-50(I)–F (Fig. S36 and S37). Therefore, it is observed that our lead halide framework (TJU-50(Cl)–F) exhibits a faster radiative decay rate (4.27 × 10^7^ s^−1^) and slower nonradiative decay rate (3.19 × 10^6^ s^−1^) compared to Na_2_(5-F-ipa), attributed to the formation of a rigid framework-type structure. Overall, these results indicate that the high RTP efficiency in TJU-50(Cl)–F is due to suppressed nonradiative recombination and efficient formation of triplet excited states.

To further clarify the differences in PLQYs and RTP behaviors among the isoreticular TJU-50 compounds, we have selected TJU-50(Cl)–Cl as a comparative example for detailed photophysical studies, focusing on the detailed electron transfer involved in intersystem crossing (ISC). Unlike TJU-50(F)–Cl, the prompt PL spectra of TJU-50(Cl)–Cl exhibit a maximum at 475 nm accompanied by a weak shoulder at ∼491 nm, while the delayed PL spectra show a very weak afterglow centered at 492 nm (Fig. S38). The average decay lifetimes of the short-lived and long-lived excited states were determined to be 2.89 ns and 1.04 ms, respectively, which are notably shorter than those of TJU-50(F)–Cl (Fig. S39). Since both frameworks consist of identical [Pb_2_Cl]^3+^ chains, the distinct emission behaviors are ascribed to the *meta*-substituted halogen groups (–F and –Cl) on the organic linkers. PL measurements of the corresponding sodium salt, Na_2_(5-Cl-ipa), revealed a bimodal prompt emission profile and a single greenish delayed band, analogous to the behavior of TJU-50(Cl)–Cl (Fig. S40 and S41). In addition, both the lifetime and PLQY of Na_2_(5-Cl-ipa) are lower than those of Na_2_(5-F-ipa) (Fig. S42 and S43). In TJU-50(X)–F (X = Cl^−^/Br^−^/I^−^), excitation of the 5-F-ipa enables efficient ISC from the singlet (S_1_) to the triplet (T_1_) state, promoted by the heavy-atom effect.^65,66^ For TJU-50(Cl)–Cl, however, the substantially lower intrinsic PL efficiency of 5-Cl-ipa results in a reduced population of initial S_1_ excitons, thereby suppressing the exciton transfer from the prompt S_1_ states to the delayed T_1_ state and leading to a negligible afterglow.^67,68^ To get deeper insight into the photophysical mechanism behind the dual emissions, we have performed molecular dynamics simulation and DFT calculations. Based on the optimized excited-state structure of TJU-50(Cl)–F, the energy gap between S_1_ and T_1_ (0.176 eV) is obviously smaller than that in TJU-50(Cl)–Cl (0.289 eV), affording a higher ISC rate in TJU-50(Cl)–F (Fig. S44). Overall, these findings indicate that the fluorine substituent on the organic ipa ligand (5-F-ipa) plays a decisive role in facilitating efficient ISC and in tailoring the RTP characteristics of the TJU-50 frameworks.

## Conclusions

In summary, this work establishes a new family of fifteen isoreticular NCS lead oxyhalide frameworks *via* a general inorganic–organic dual-site strategy, combining inorganic [PbX_2_O_6_] building blocks and V-shaped *meta*-dicarboxylate linkers. This approach provides an effective pathway for constructing lead oxyhalide-based NLO-active units by breaking the symmetry of the [PbX_6_] octahedra and subsequently controlling their ordered superposition *via* the oriented coordination with *meta*-dicarboxylates. The resultant compounds predominantly exhibit phase-matching SHG responses (6.0–11.0 × KDP @ 1064 nm) and birefringence values of 0.056–0.179 @ 546 nm. By combining the tunability of halide substitution in the [PbX_2_O_6_] units and *meta*-substituents in isophthalate ligands, we have successfully modulated both bandgaps and the primitive alignments to deliver the highest SHG performance in the series: 11.0 × KDP @ 1064 nm and 1.0 × KTP @ 2000 nm for TJU-50(I)–Br and a UV cutoff edge at 335 nm for TJU-50(I)–Br. Structural analysis and first-principles calculations reveal that the enhanced optical nonlinearity mainly originates from coordination-driven superposition of polar [PbX_2_O_6_] and ipa-based primitives. Moreover, the synergistic interaction between phosphorescent ipa-based ligands and photoactive lead halide chains leads to the highest PLQY up to 93.05% and long-lived afterglow emission lifetimes up to 7.36 ms. Overall, this work establishes a new and versatile inorganic–organic dual-site strategy for the rational design of high-performance NLO-active hybrid lead halides, offering a new perspective for developing ultrastable, multifunctional metal halide materials for advanced optical technologies.

## Author contributions

Chen Sun: conceptualization, writing – original draft, data curation, formal analysis, software, investigation, validation, methodology, visualization. Xingxing Jiang: methodology, software, validation, visualization. Feiyuan Gong, Xiaotian Zhang, and Zheshuai Lin: software, visualization. Chao Wu: writing – review & editing, resources, formal analysis, methodology, investigation, visualization. Chi Zhang: conceptualization, writing – review & editing, resources, formal analysis, methodology, visualization, supervision, investigation. Honghan Fei: conceptualization, methodology, writing – review & editing, writing – original draft, funding acquisition, project administration, supervision, resources, validation, visualization, investigation, formal analysis.

## Conflicts of interest

There are no conflicts to declare.

## Supplementary Material

SC-OLF-D6SC02530G-s001

SC-OLF-D6SC02530G-s002

## Data Availability

The data supporting this article have been included as part of the supplementary information (SI). Supplementary information: experimental details, additional characterization, SI data, and SI tables. See DOI: https://doi.org/10.1039/d6sc02530g. CCDC 2392252–2392266 contain the supplementary crystallographic data for this paper.^69*a*–*o*^
