# Supplementary material for: A universal synthetic strategy for noncentrosymmetric lead oxyhalide frameworks with strong second-harmonic generation
Source: Chem Sci. 2026 Aug 25;17(40):19563–75. doi: 10.1039/d6sc02530g (PMC13537042; doi:10.1039/d6sc02530g)
Supplement: Supplementary file 1 [file SC-017-D6SC02530G-s001.pdf]

Supplementary Information (SI) for Chemical Science.
This journal is © The Royal Society of Chemistry 2026

Supporting Information

A Universal Synthetic Strategy for Noncentrosymmetric Lead Oxyhalide Frameworks with Strong Second-Harmonic Generation

Chen Sun,[†a] *Xingxing Jiang,*[†b] *Feiyuan Gong,*[a] *Xiaotian Zhang,*[a] *Chao Wu,*[a] *Zheshuai Lin,*[b] *Chi Zhang,*[*a] *and Honghan Fei*[*a]

[a] Shanghai Key Laboratory of Chemical Assessment and Sustainability, School of Chemical Science and Engineering, Tongji University, Shanghai 200092 China

[b] Technical Institute of Physics and Chemistry, Chinese Academy of Sciences Beijing 100190, (China).

E-mail: wuc@tongji.edu.cn; chizhang@tongji.edu.cn; fei@tongji.edu.cn

[†]These authors contribute equally to this work.

Materials, Methods, and Results

Materials. All starting reagents, materials, and solvents were available from commercial supplier and used without further purification. Lead chloride ($PbCl_2$, 99.0%, Aladdin), lead bromide ($PbBr_2$, 99.9%, Aladdin), lead iodide (PbI_2, 99.9%, Aladdin), isophthalic acid (ipa, $C_8H_6O_4$, 99%, Adamas), 5-bromoisophthalic acid (5-Br-ipa, $C_8H_5O_4Br$, 98%, Adamas), 5-chloroisophthalic acid (5-Cl-ipa, $C_8H_5O_4Cl$, 98%, Adamas), 5-fluoroisophthalic acid (5-F-ipa, $C_8H_5O_4F$, 98%, Adamas), 5-methylisophthalic acid (5-CH_3-ipa, $C_9H_8O_4$, 98%, Adamas) and 5-hydroxyisophthalic acid (5-OH-ipa, $C_8H_6O_5$, 99%, Adamas), *N*,*N*'-dimethylacetamide (DMA, >99.5%, Greagent), acetonitrile (CH_3CN, >99%, Greagent), ethanol (EtOH, >99%, Greagent), perchloric acid (70.0% in H_2O, SCR), and deionized water was obtained from a Barnstead Pacific RO water purification system.

Methods. Synthesis of TJU-50(Cl)-Br/Cl/F/CH$_3$ ([Pb$_2$Cl]][5-Br/Cl/F/CH$_3$-ipa]$_2$•G). The mixture of 0.2224 g $PbCl_2$ (0.8 mmol), 5-Br-ipa (0.8 mmol, 0.1441 g)/5-Cl-ipa (0.8 mmol, 0.1605 g)/5-F-ipa (0.8 mmol, 0.1960 g) and 5-CH_3-ipa (0.8 mmol, 0.1441 g) was adequately dispersed in mixed solution of DMA (4 mL), EtOH (2 mL) and hydrochloric acid (0.2 mL). The suspension was stirred for 30 min and followed by transferring in a 25 mL Teflon-lined autoclave. The autoclave was heated at 140 °C for 72 h and then slowly cooled to room temperature at a rate of 5 $°C \cdot h^{-1}$. The colorless prism-shaped crystals were isolated by vacuum filtration, rinsed with DMA and EtOH for three times, and then dried under vacuum at 60 °C. The yields of as-synthesized TJU-50(Cl) are 74-78 % based on total Pb content. Anal. cal. for TJU-50(Cl)-Br: calculated: C, 22.02%, H, 1.44%, N, 1.43%, found: C, 21.99%, H, 1.49%, N, 1.42%; TJU-50(Cl)-Cl: calculated: C, 24.21%, H, 1.58%, N, 1.57%, found: C, 24.22%, H, 1.56%, N, 1.54%; TJU-50(Cl)-F: calculated: C, 25.13%, H, 1.64%, N, 1.63%, found: C, 25.12%, H, 1.63%, N, 1.65%; TJU-50(Cl)-CH_3: calculated: C, 28.19%, H, 2.37%, N, 1.64%, found: C, 28.20%, H, 2.35%, N, 1.62%.

Synthesis of TJU-50(Br)-Br/Cl/F/CH$_3$ ([Pb$_2$Br]][5-Br/Cl/F/CH$_3$-ipa]$_2$•G). Crystals of TJU-50(Br) can be synthesized in the same manner as for TJU-50(Cl) but with $PbBr_2$ in place of $PbCl_2$ at the same molar ratio. Yields: 66-72 % based on total Pb content.

Synthesis of TJU-50(I)-Br/Cl/F/CH$_3$ ([Pb$_2$I]][5-Br/Cl/F/CH$_3$-ipa]$_2$•G). Crystals of TJU-50(I) can be synthesized in the same manner as for TJU-50(Cl) but with PbI_2 in place of $PbCl_2$ at the same molar ratio. Yields: 69-73 % based on total Pb content.

Synthesis of TJU-51(Cl)-OH ([Pb_2Cl][5-OH-ipa]$_2$•G). The mixture of 0.1669 g $PbCl_2$ (0.6 mmol), 0.1457 g 5-OH-ipa (0.8 mmol) and hydrochloric acid (0.2 mL) was adequately dispersed in mixed solution of DMA (4 mL), CH_3CN (2 mL). The suspension was stirred for 30 min and then the reactor was sealed into a stainless steeled container and transferred into an oven kept at 140 °C for 3 days and then slowly cooled to room temperature. The colorless needle-like crystals were isolated by vacuum filtration, rinsed with DMA and EtOH, and then dried under vacuum at 60 °C. Anal. cal. for TJU-51(Cl)-OH: calculated: C, 25.28%, H, 1.77%, N, 1.64%; observed: C, 25.25%, H, 1.78%, N, 1.62%.

Synthesis of TJU-51(Br)-OH ([Pb_2Br][5-OH-ipa]$_2$•G). Crystals of TJU-51(Br)-OH can be synthesized in the same manner as for TJU-51(Cl)-OH but with $PbBr_2$ in place of $PbCl_2$ at the same molar ratio.

Synthesis of TJU-51(I)-OH ([Pb_2I][5-OH-ipa]$_2$•G). Crystals of TJU-51(I)-OH can be synthesized in the same manner as for TJU-51(Cl)-OH but with PbI_2 in place of $PbCl_2$ at the same molar ratio.

Single-Crystal Crystallography Studies. Single crystals with suitable size were chosen under an optical microscope (NIKON ECLIPSE LV100N POL) and carefully mounted onto a glass fiber. The crystal structure data were analyzed using graphite-monochromated Mo-Kα radiation (λ = 0.71073 Å), which was operated at 50 kV and 30 mA on a Bruker SMART APEX II CCD area detector X-ray diffractometer. Diffraction data were processed with the APEX3 software package, integrated using SAINT, and further corrected for absorption effects using SADABS. The space group was determined by systematic absences, E-statistics, agreement factors for equivalent reflections, and successful refinement of the structure. The crystal structure was solved by direct methods and expanded routinely and refined by full matrix least-squares analysis of F^2 against all reflections.[1] All nonhydrogen atoms were refined with anisotropic thermal displacement parameters.[2] Thermal parameters for hydrogen atoms were tied to the isotropic thermal parameter of the atom to which they are bonded. Further details of crystallographic data and structural refinement are summarized in Table S1-S35. Simulated powder patterns were calculated by Mercury software applying the crystallographic information from the single-crystal X-ray diffraction data to obtain the powder XRD diffraction. The single crystal X-ray structure data files of TJU-50(X)-F/Cl/Br/CH_3 (X=Cl, Br, I) and TJU-50(X)-OH at 298 K have been deposited in the Cambridge Crystallographic Data Centre (CCDC: 2392252 for TJU-50(Cl)-F, 2392253 for TJU-50(Br)-F and 2392254 for TJU-50(I)-F;

2392255 for TJU-50(Cl)-Cl, 2392257 for TJU-50(Br)-Cl and 2392256 for TJU-50(I)-Cl; 2392265 for TJU-50(Cl)-Br, 2392258 for TJU-50(Br)-Br and 2392259 for TJU-50(I)-Br; 2392260 for TJU-50(Cl)-CH_3, 2392266 for TJU-50(Br)-CH_3 and 2392261 for TJU-50(I)-CH_3; 2392262 for TJU-51(Cl)-OH, 2392263 for TJU-51(Br)-OH and 2392264 for TJU-51(I)-OH respectively). These data can be obtained free of charge from https://www.ccdc.cam.ac.uk/structures/.

General characterization. The powder X-ray diffraction (PXRD) analysis was performed on a Bruker D2 Phaser X-ray diffractometer equipped with Cu-Kα radiation (λ= 1.5418 Å) at a voltage of 30 kV and a current of 10 mA. The diffraction patterns were scanned at ambient temperature, with a scan speed of 1 sec/step, a step size of 0.02° in 2θ, and a 2θ range of 5~40°. C/H/N elemental analysis was obtained on a Varian EL III element analyser. The UV-Vis-NIR transmittance spectra were measured at room temperature with crystal samples. Data were collected on a Cary 5000 UV-Vis-NIR spectrophotometer scanning in the range 200-2000 nm.

Chemical stability. ~100 mg of as-synthesized TJU-50(X)-F/Cl/Br/CH_3 and TJU-50(X)-OH crystals were incubated in the boiling water or diluted HCl aqueous solution (pH = 5) or NaOH aqueous solution (pH = 12) for 24 hours at room temperature. The crystals after chemical treatments were isolated by filtration and dried in the 60 °C oven before PXRD and mass balance treatments.

Thermogravimetric analysis. Thermogravimetric analysis (TGA) was carried out using a TGA Q500 differential thermal analyzer. The samples were heated from room temperature to 800 °C with a heating rate of 10 °C/min under N_2 stream (60 mL/min).

Powder Second-Harmonic Generation Measurements. The measurements of the powder frequency-doubling effects were performed at room temperature on sieved samples using the modified method of Kurtz and Perry with laser radiation of λ = 1064 and 2000 nm generated by a Radiant Tunable Laser System (Amplitude, Horizon II Mid-band Optical Parametric Oscillator; 5Hz).[3] The powder SHG efficiency is related to the particle size, so the crystal samples were ground and sieved into six distinct particle size ranges (26-50, 50-74, 74-105, 105-150 and 150-200 μm), which were pressed into disks with a diameter of 6 mm that were placed between glass microscope slides and secured with tape in a 1 mm thick aluminum holder. The commercial KH_2PO_4 (KDP) and KTP samples were ground and sieved into the same particle size ranges and used as the reference with 1064 and 2000 nm radiation. The intensities

of the frequency-doubled output emitted from the crystalline samples were measured using an Ocean Optics Maya2000 Pro spectrometer.

Birefringence Measurements. The birefringences of all crystalline samples were assessed with a polarizing microscope (ZEISS Axio scope A1) equipped with a Berek compensator. The wavelength of the light source was 546 nm. The birefringences were calculated according to the following equation:

$$\Delta R \text{ (retardation)} = |n_e - n_o| \times T = \Delta n \times T \qquad (1)$$

where ΔR denotes the optical path difference, Δn represents the birefringence, and T is the thickness of the crystal. The positive and negative rotation of compensation affords the relative retardation. Transparent regular TJU-50 and TJU-51 crystals were chosen for the measurements to improve the accuracy of the birefringence measurements.

Photoluminescence characterization. The PL spectra were performed on an Edinbergh FLS1000 fluorescence spectrometer. The photoluminescence quantum yield (PLQY) was achieved by incorporating an integrating sphere into the FL1000 spectrofluorometer. The PLQY was calculated based on the equation: $\eta_{QE} = I_S/(E_R\text{-}E_S)$, where I_S represents the luminescence emission spectrum of the sample, E_R is the spectrum of the excitation light from the empty integrated sphere (without the sample), and E_S is the excitation spectrum for exciting the sample. Delayed PL spectra were obtained from the FLS1000 PL spectrometer by using a flash lamp as the excitation source equipped with a time-gate module. The average lifetimes were obtained by exponential fitting. The dynamic delayed PL spectra were collected on Ocean Optics spectrophotometer (QE65 Pro). The corresponding Commission Internationale Eclairage (CIE) chromaticity coordinates were calculated based on PL emission spectrum.

Theoretical calculations. The density functional theory (DFT) calculations of geometry optimization and energy simulation were performed by using Cambridge Serial Total Energy Package (CASTEP) module of the Materials Studio (MS) software package, based on the plane-wave basis set and pseudo-potentials by first principles calculations.[4-6] The functional developed by Perdew, Burke and Ernzerhof (PBE) in the generalized gradient approximation (GGA) form was adopted to describe the exchange-correlation energy.[7] Optimized norm-conserving pseudopotentials were adopted to model the effective interaction between the valence electrons and atom cores, which allows the choice of a relatively small plane-wave basis set without compromising the computational accuracy. A kinetic energy cutoff of 900 eV and dense Monkhorst-Pack k-point mesh spanning less than 0.03 Å^{-1} in the Brillouin zone were

chosen.[8] The band gap calculated by standard DFT is smaller than the experimental data due to the discontinuity in the exchange-correlation functional, so a scissor operator was used to shift the conduction band upward to agree with the measured data. Based on the scissor-operator-corrected electronic band structure, the imaginary part of the dielectric function from the electronic transition between the valence band (VB) and the conduction band (CB) can be calculated. The real part of the dielectric function, i.e., the refractive indices, can be determined by a Kramers-Kronig transform.[9, 10]

For better understanding orbital donations to second-harmonic generation (SHG) response SHG efficiency, SHG-weighted electron cloud calculations were calculated. In the SHG-weighted electron cloud calculations, the probability densities of all occupied (valence) or unoccupied (conduction) states projected onto real space are multiplied by a weighting factor that is related to the contribution to SHG efficiency by the virtual electron (VE) and virtual hole (VH) processes.[11] This ensures that the quantum states irrelevant to SHG are not shown in the occupied or unoccupied "SHG-densities", while the orbitals vital to SHG are intuitively highlighted in real space.

Gaussian Calculations. Density functional theory (DFT) calculations for structural building units were performed at the B3LYP/def2-TZVP level of theory with the Gaussian 09 program. Geometries of the molecular units were kept the same as their geometries in the crystal structure, and the total magnitude of β was calculated from the tensor components by using the following equations[12]:

$$\beta_i = \sum_j \beta_{ijj} + \beta_{jij} + \beta_{jji} \quad (2)$$

$$\beta_{total} = \sqrt{{\beta_x}^2 + {\beta_y}^2 + {\beta_z}^2} \quad (3)$$

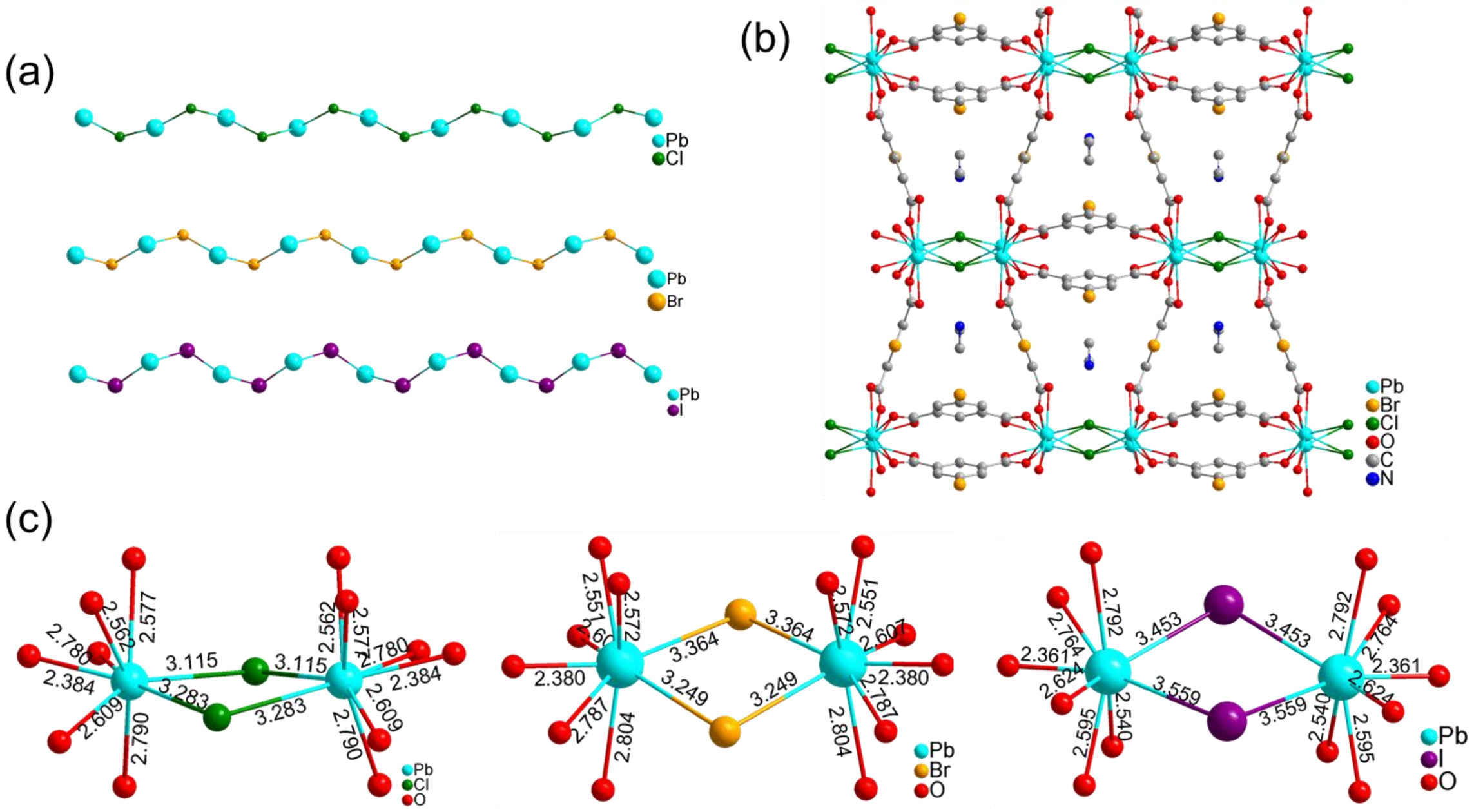

Fig. S1 (a) Crystallographic view of a single $[Pb_2X]^{3+}$ (X = Cl, Br, I) chain of TJU-50(X)-Br along *a*-axis. (b) Crystallographic view of TJU-50(X)-Br along the *c*-axis, the guest $(CH_3)_2NH_2^+$ cations are orderly distributed in the porosity. (c) The distorted $[PbX_2O_6]$ SBUs and bond lengths.

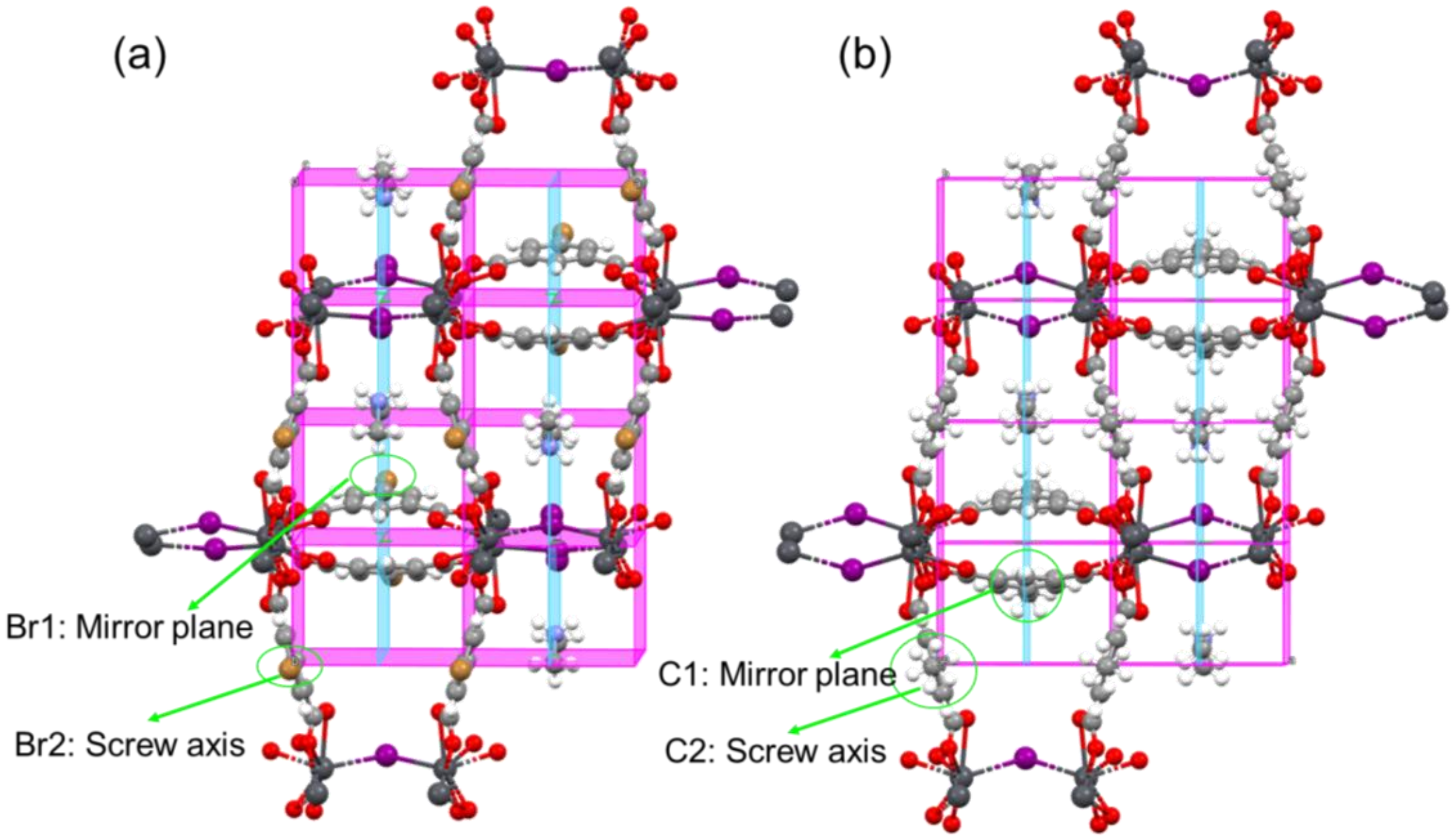

Fig. S2 (a) Crystallographic view of TJU-50(I)-Br along *c*-axis, and Br atoms residing in two different chemical environments of the ligand are highlighted. (b) Crystallographic view of TJU-50(I)-CH_3 along *c*-axis. The CH_3 groups residing in two different chemical environments of the ligand are highlighted and both are disordered.

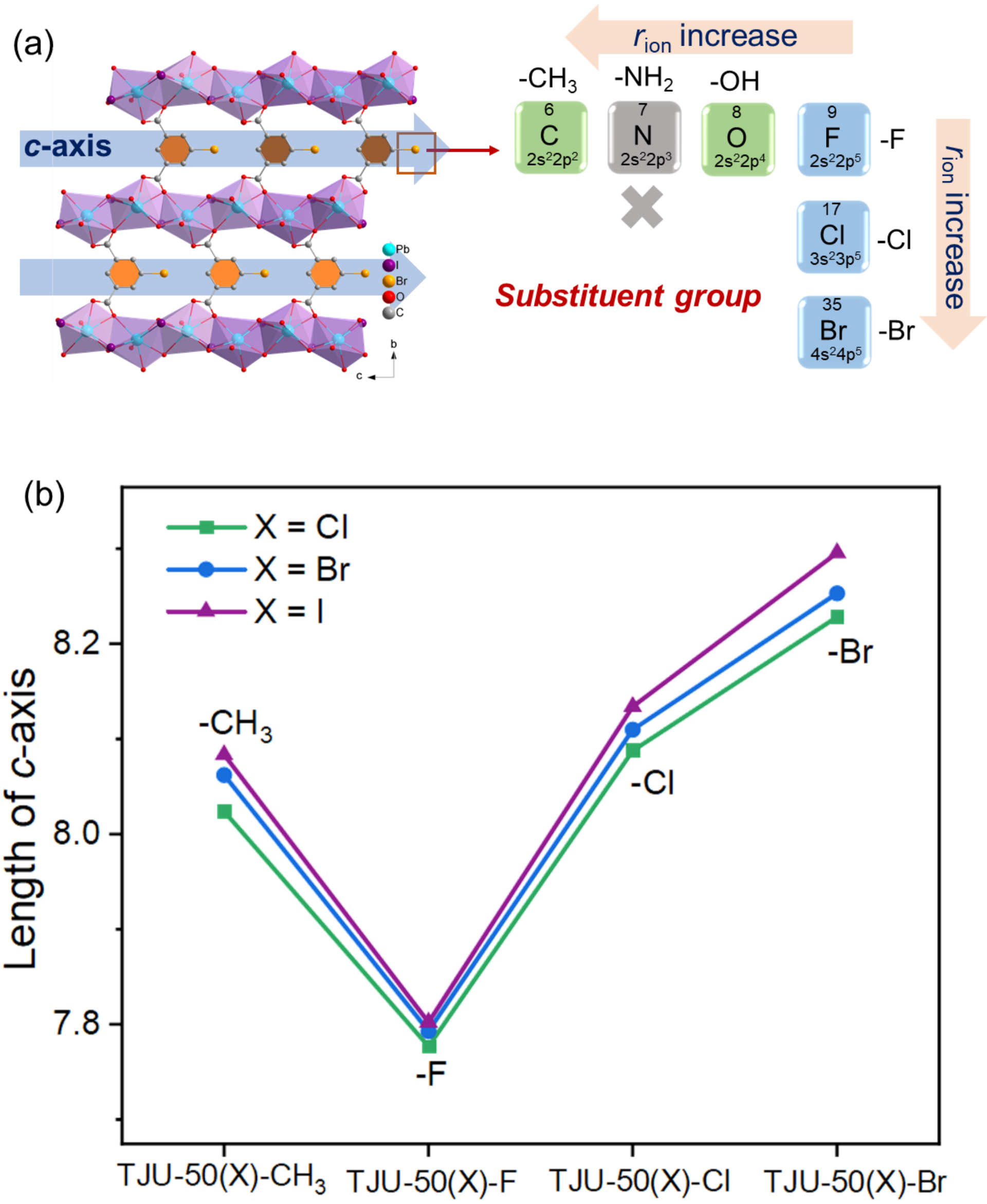

Fig. S3 (a) X-ray crystallographic views along the *a*-axis and the selected substituent groups of 5-meta-ipa derivatives. (b) Lattice parameter *c* of different TJU-50 structures.

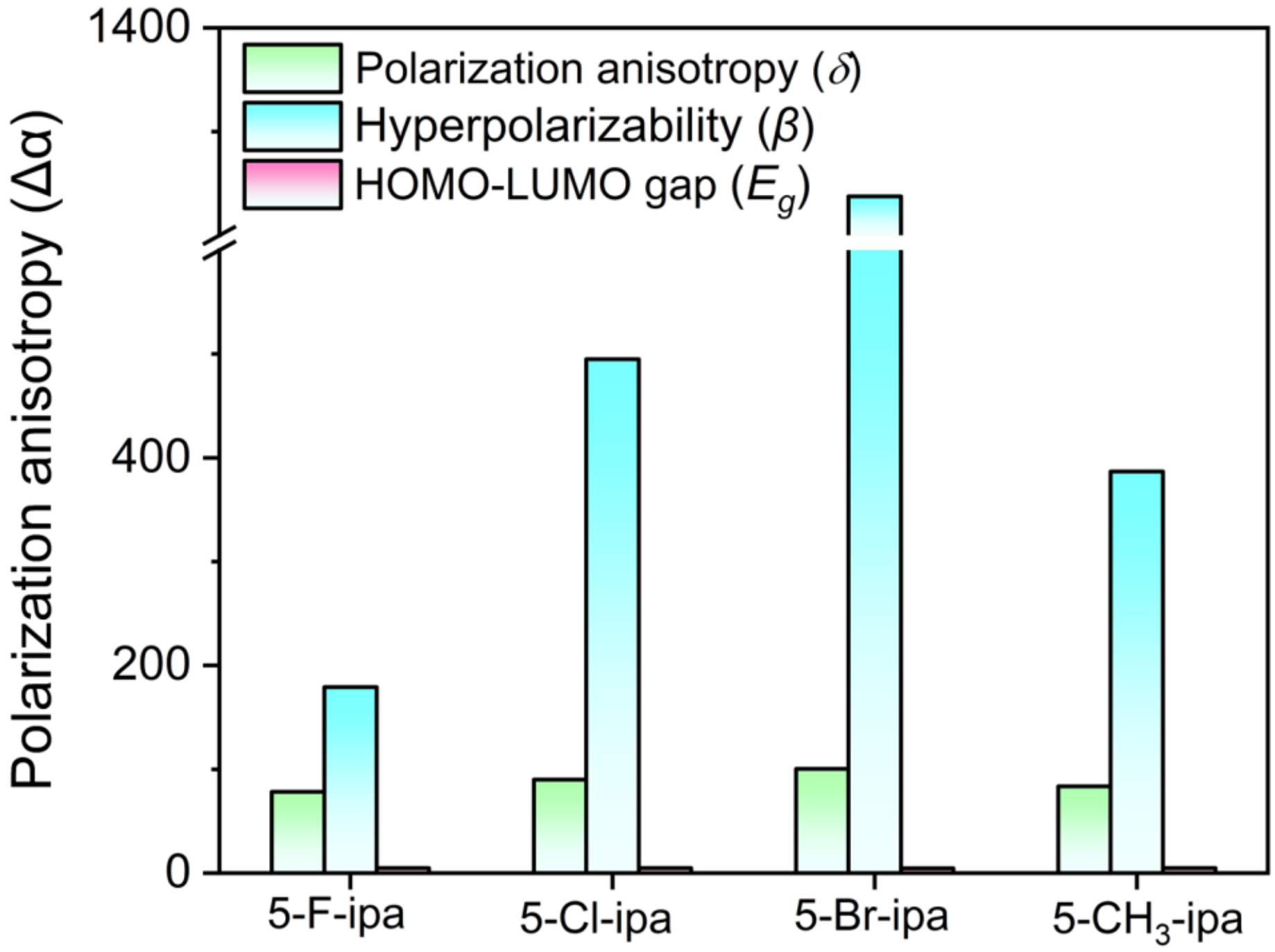

Fig. S4 Key NLO parameters of 5-F-ipa, 5-CH_3-ipa, 5-Cl-ipa and 5-Br-ipa, including polarizability anisotropy (δ), hyperpolarizability (β) and HOMO-LUMO gap (E_g).

(a)

(b)

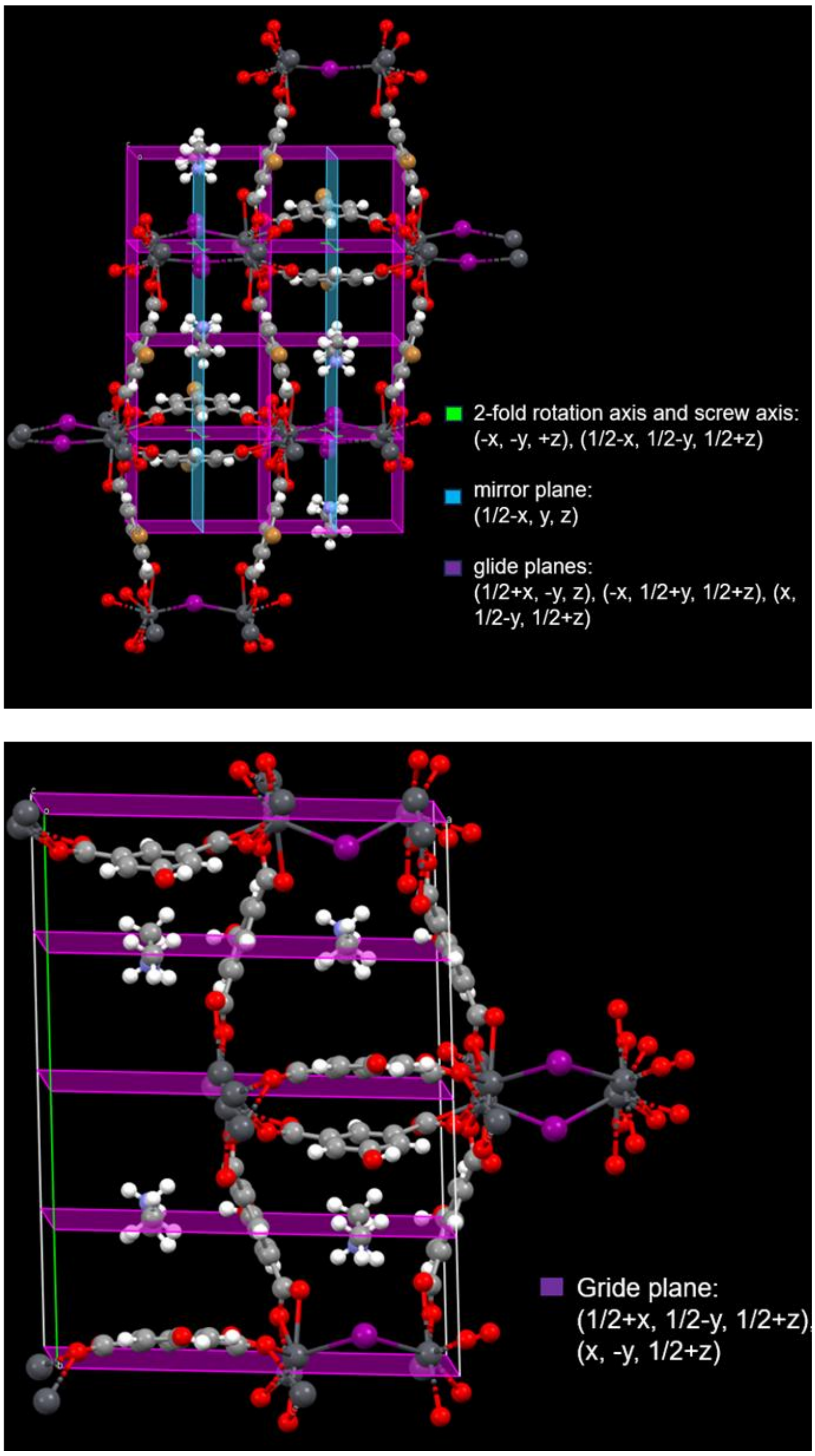

Fig. S5 Symmetry elements of TJU-50(X)-Br and TJU-51(X)-OH.

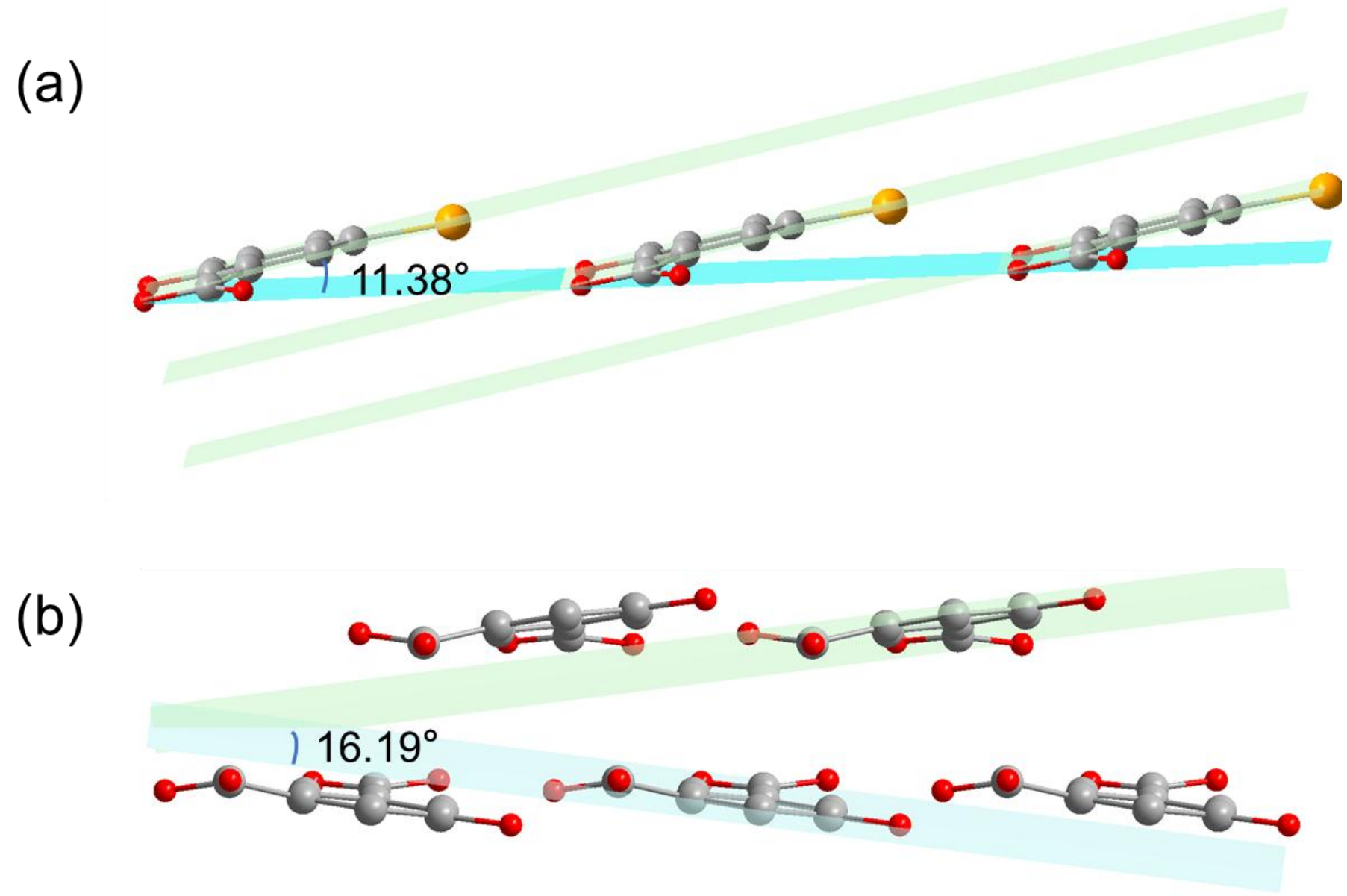

Fig. S6 (a) Dihedral angle of 5-Br-ipa linkers with the *010* plane in TJU-50(X)-Br. (b) Dihedral angle between two 5-OH-ipa linkers in TJU-51(X)-OH.

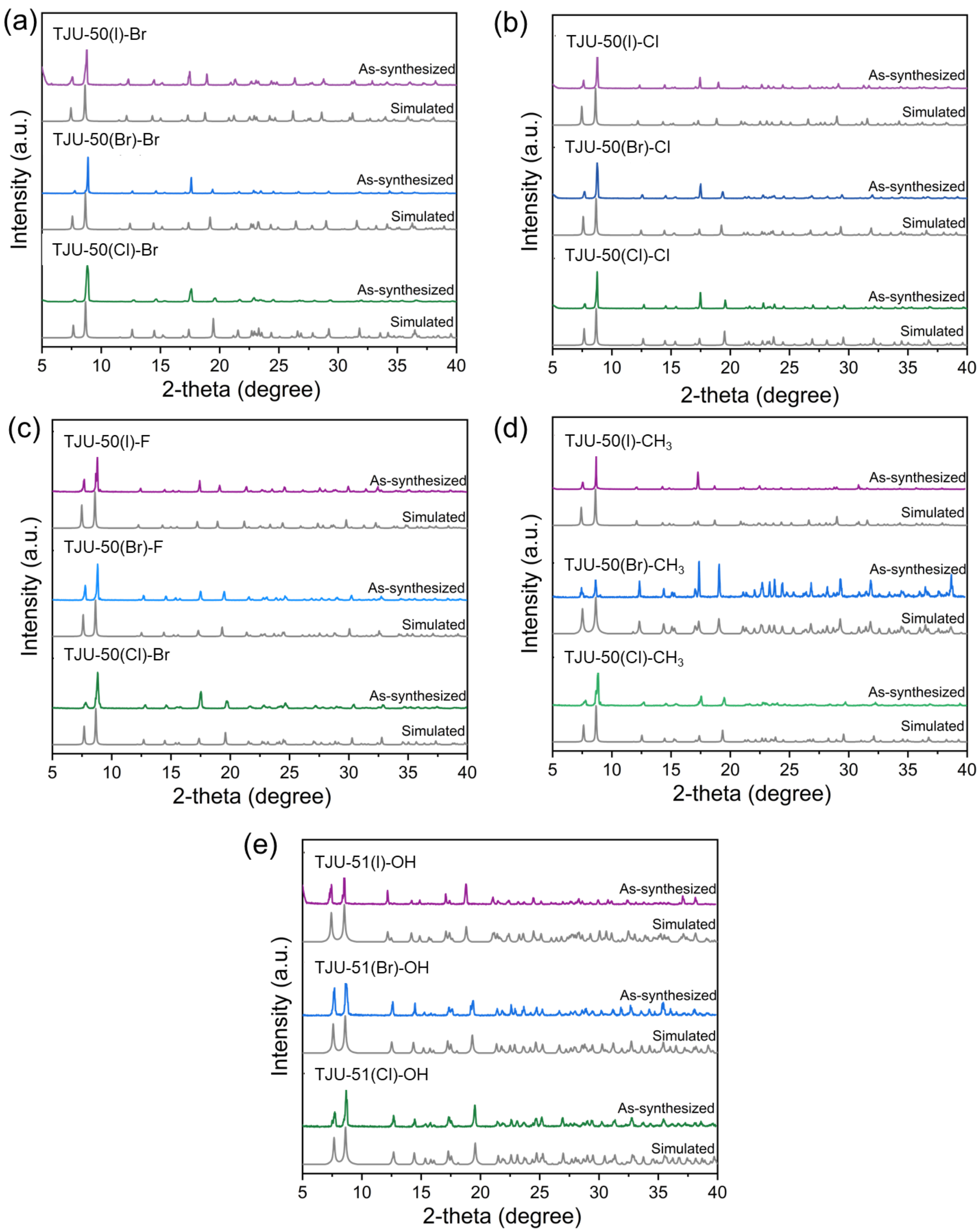

Fig. S7 The simulated and experimental PXRD patterns of as-synthesized TJU-50 and TJU-51.

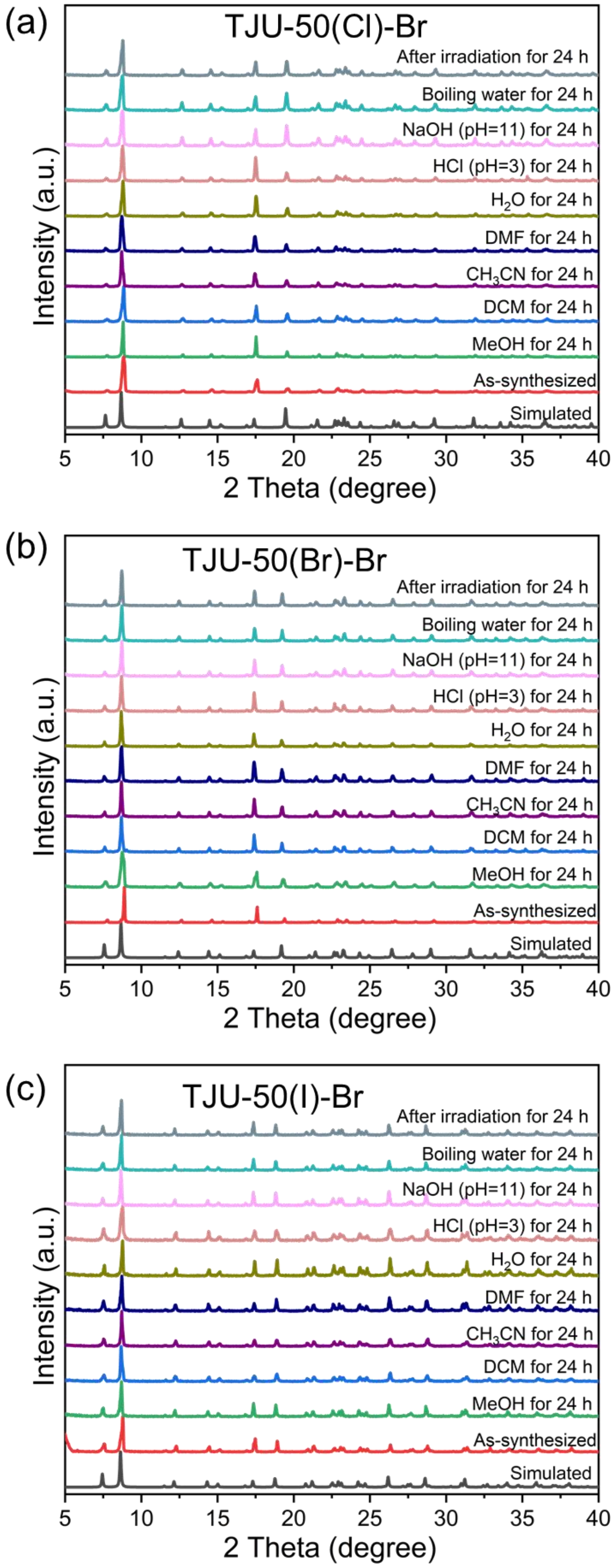

Fig. S8 PXRD of TJU-50(Cl)-Br (a), TJU-50(Br)-Br (b) and TJU-50(I)-Br (c) before and after chemical treatments and continuous irradiation (300 W Xenon lamp, 24 mW/cm^2).

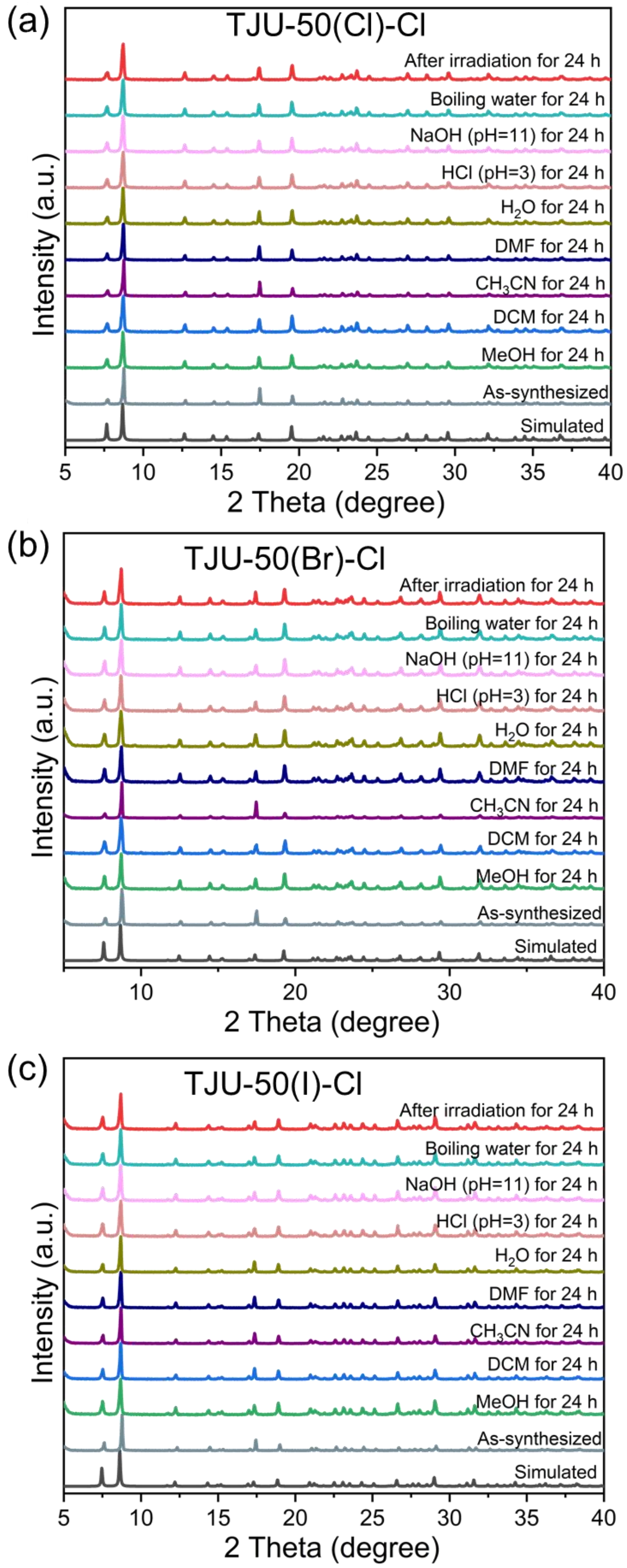

Fig. S9 PXRD of TJU-50(Cl)-Cl (a), TJU-50(Br)-Cl (b) and TJU-50(I)-Cl (c) before and after chemical treatments and continuous irradiation (300 W Xenon lamp, 24 mW/cm^2).

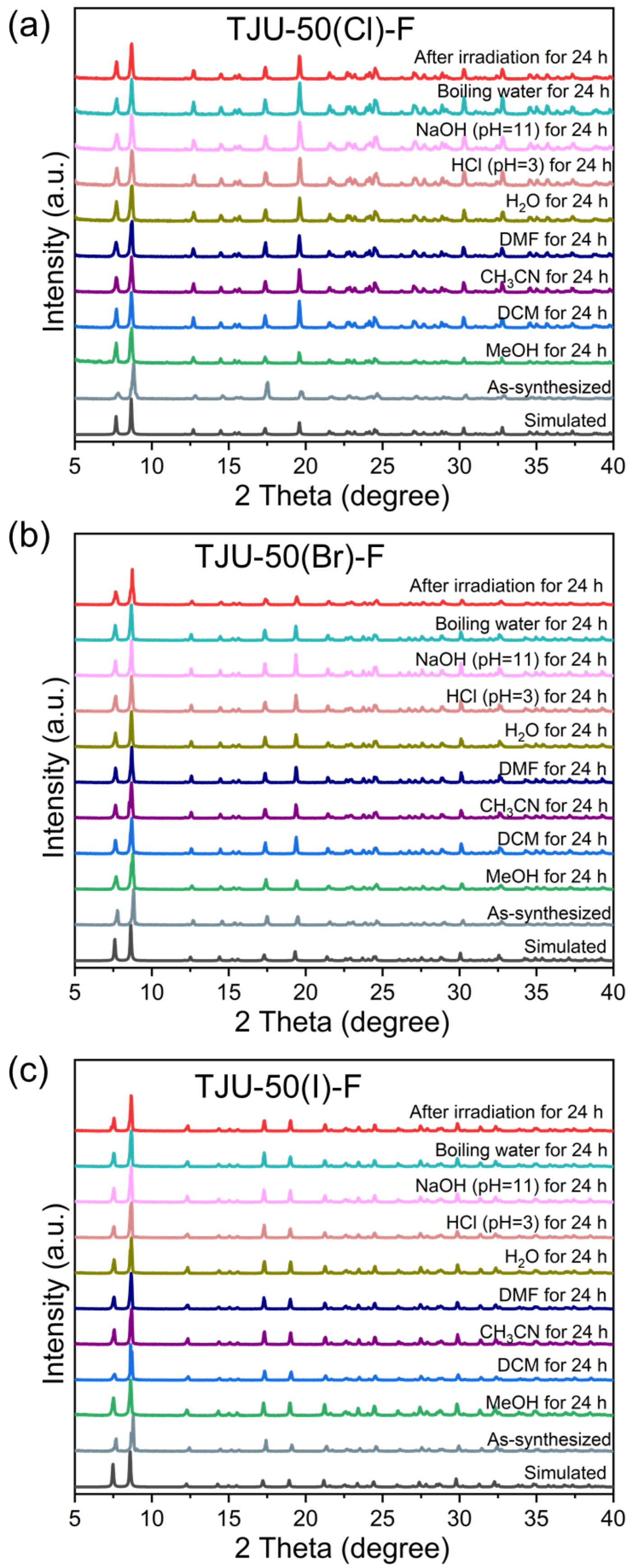

Fig. S10 PXRD of TJU-50(Cl)-F (a), TJU-50(Br)-F (b) and TJU-50(I)-F (c) before and after chemical treatments and continuous irradiation (300 W Xenon lamp, 24 mW/cm^2).

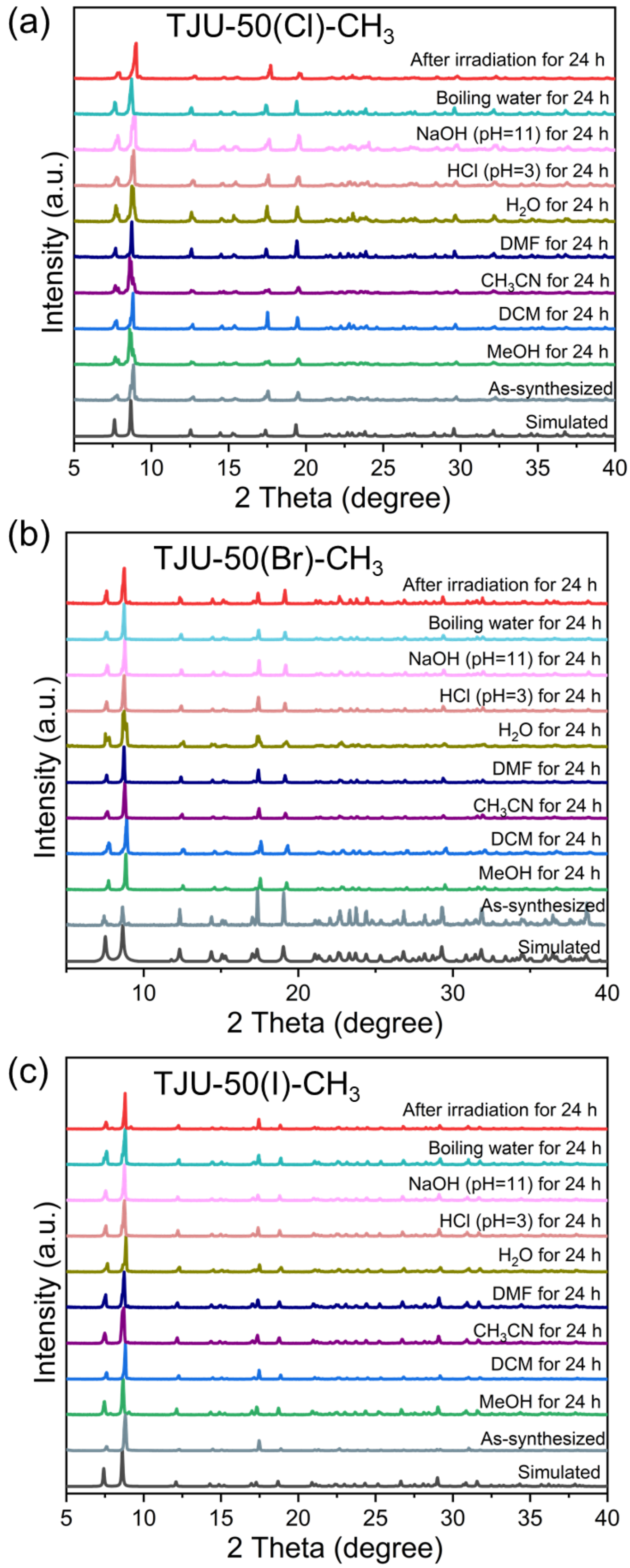

Fig. S11 PXRD of TJU-50(Cl)-CH_3 (a), TJU-50(Br)-CH_3 (b) and TJU-50(I)-CH_3 (c) before and after chemical treatments and continuous irradiation (300 W Xenon lamp, 24 mW/cm^2).

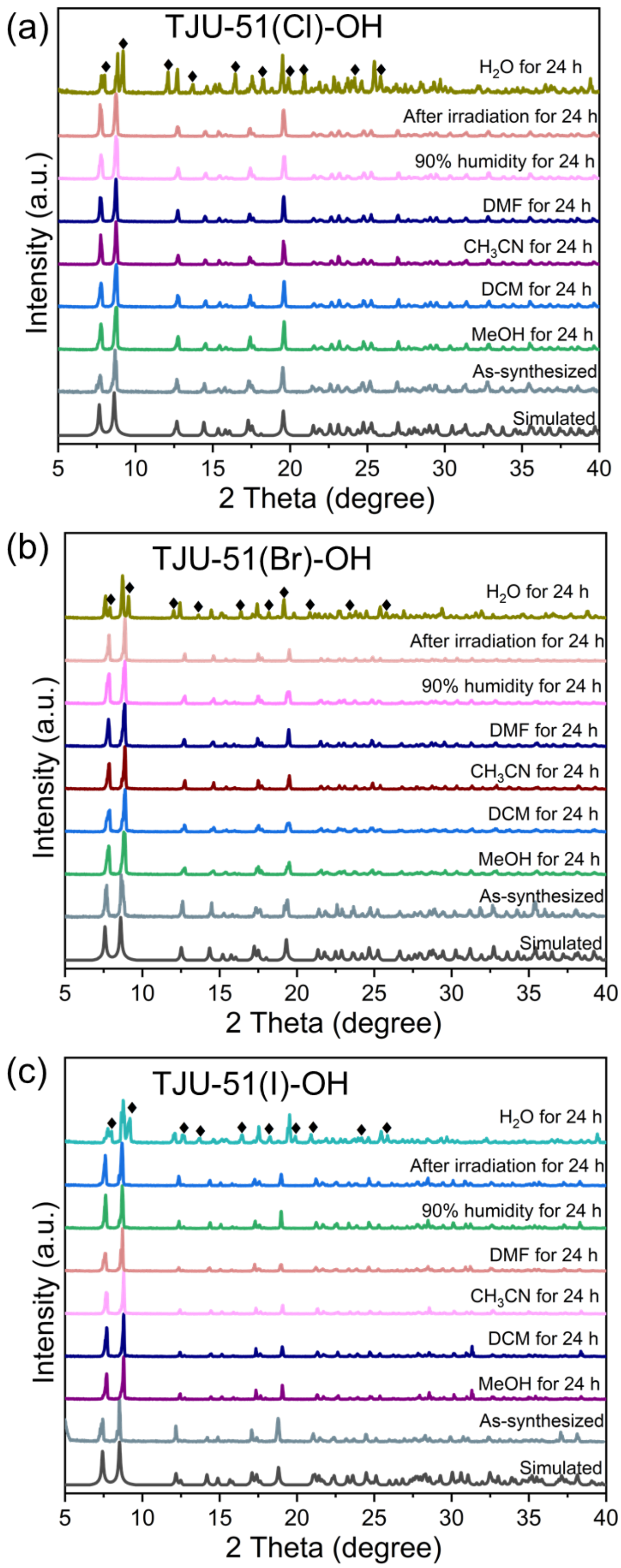

Fig. S12 PXRD of TJU-51(Cl)-OH (a), TJU-51(Br)-OH (b) and TJU-51(I)-OH (c) before and after chemical treatments and continuous irradiation (300 W Xenon lamp, 24 mW/cm^2).

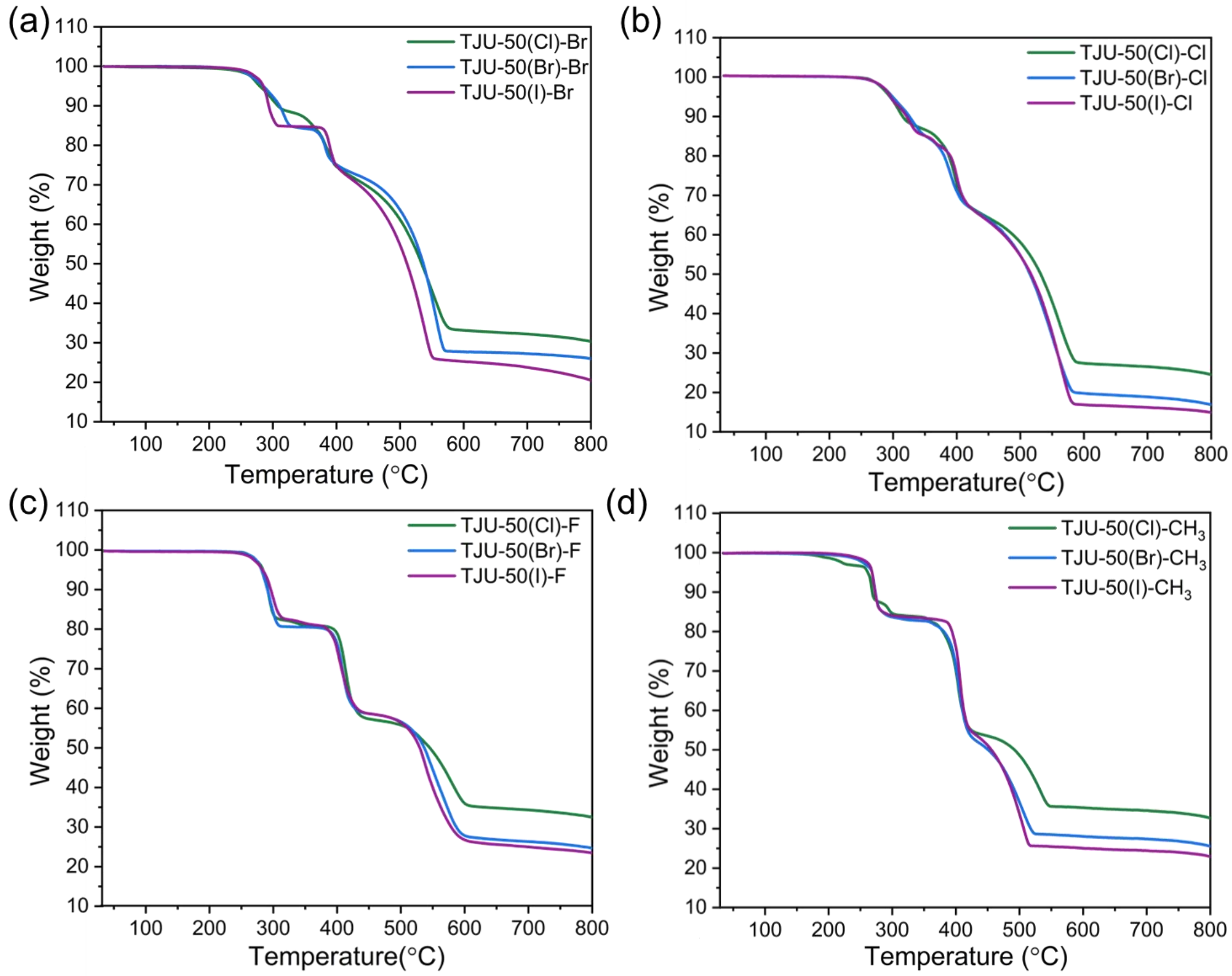

Fig. S13 Thermogravimetric analysis of TJU-50(X)-Br (a), TJU-50(X)-Cl (b), TJU-50(X)-F (c) and TJU-50(X)-CH_3 (d).

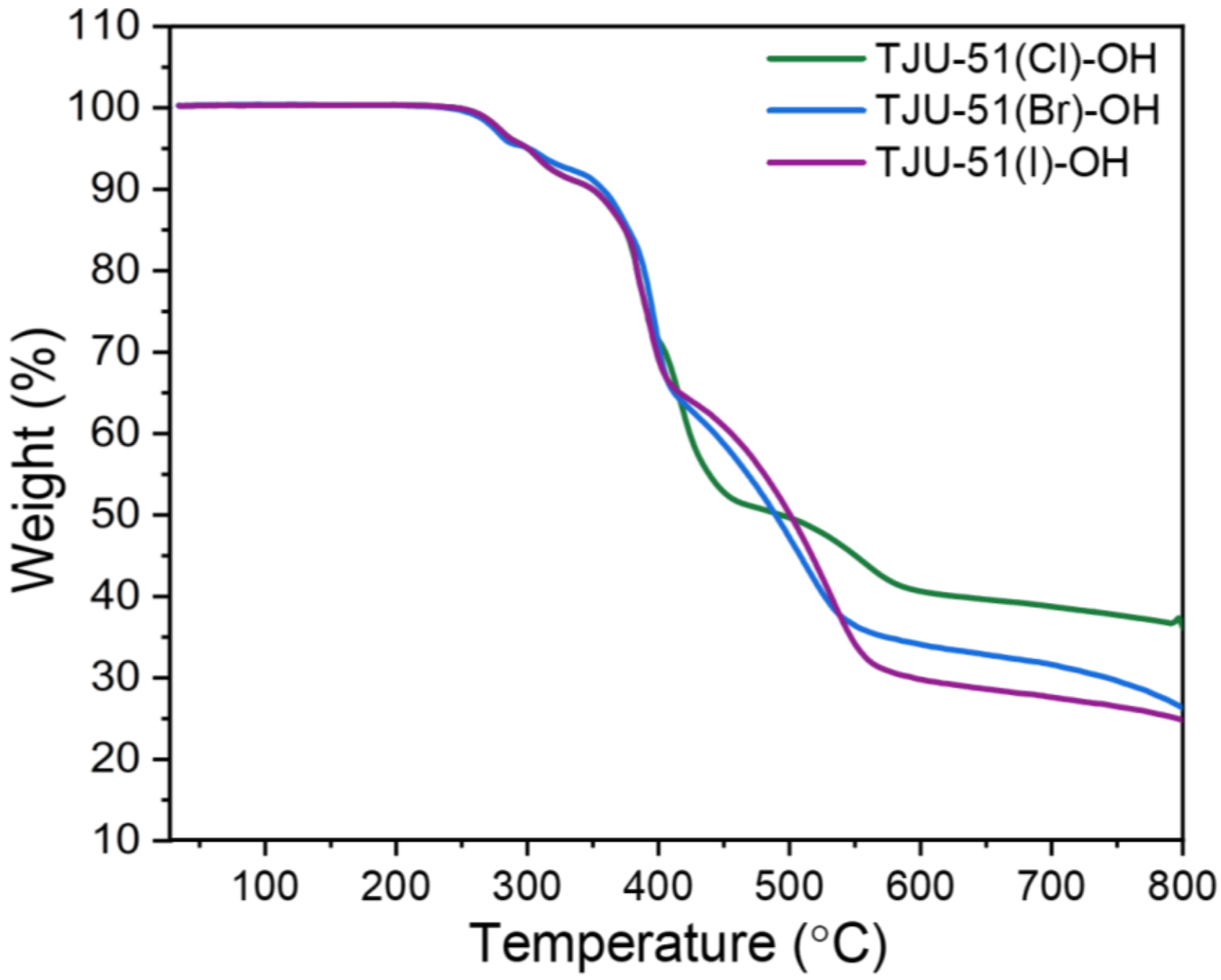

Fig. S14 Thermogravimetric analysis of TJU-51(X)-OH.

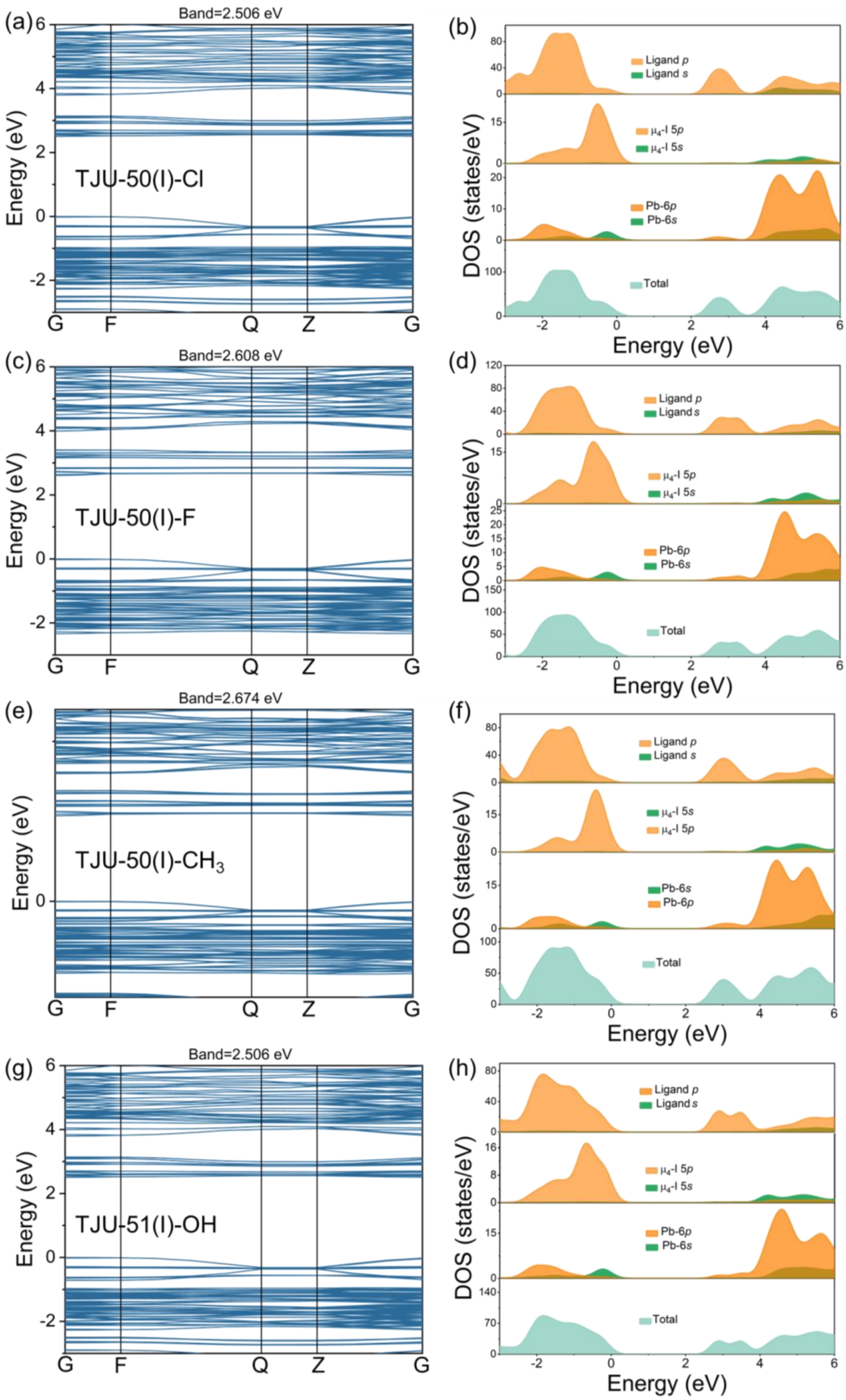

Fig. S15 The calculated band structures and projected density of states of TJU-50(I)-Cl (a-b), TJU-50(I)-F (c-d), TJU-50(I)-CH_3 (e-f), TJU-51(I)-OH (g-h).

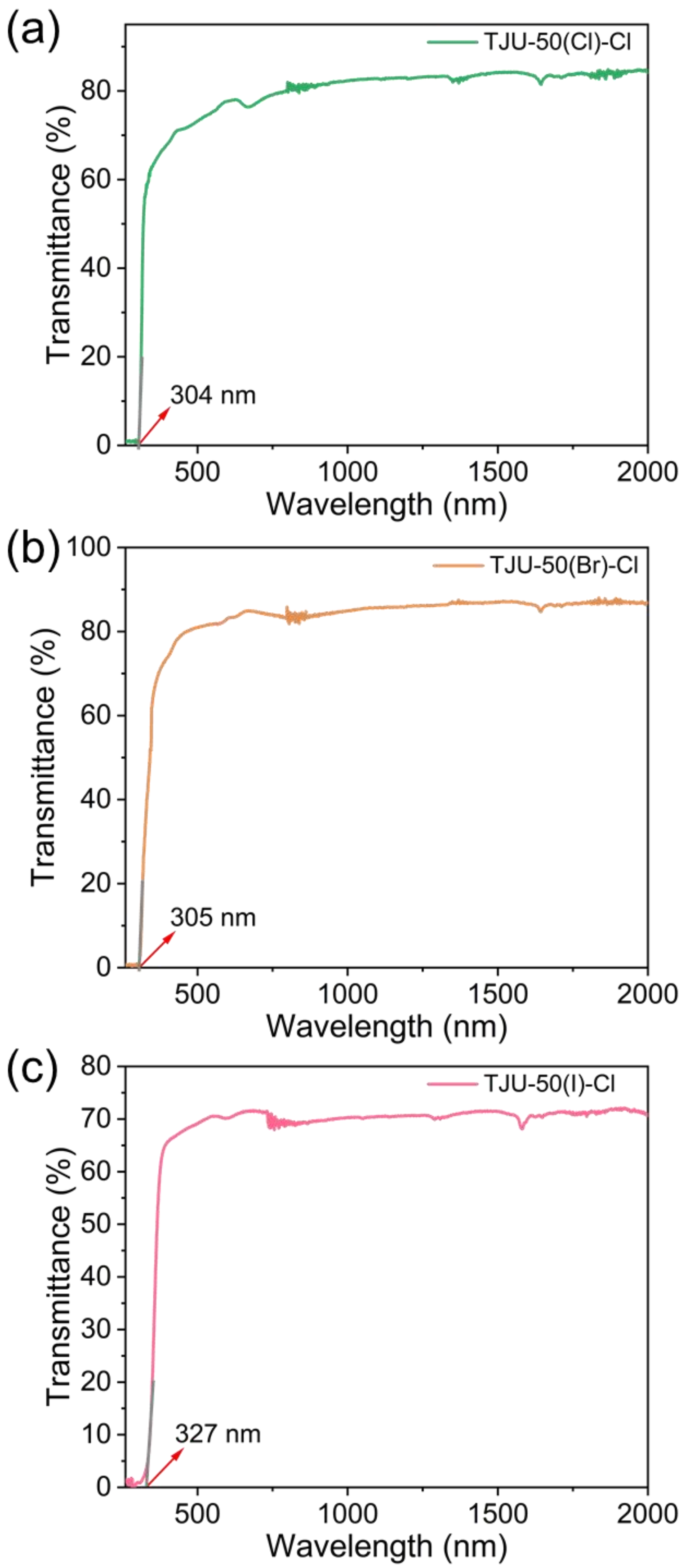

Fig. S16 UV-vis-NIR transmittance spectra of TJU-50(Cl)-Cl (a), TJU-50(Br)-Cl, (b) TJU-50(I)-Cl (c).

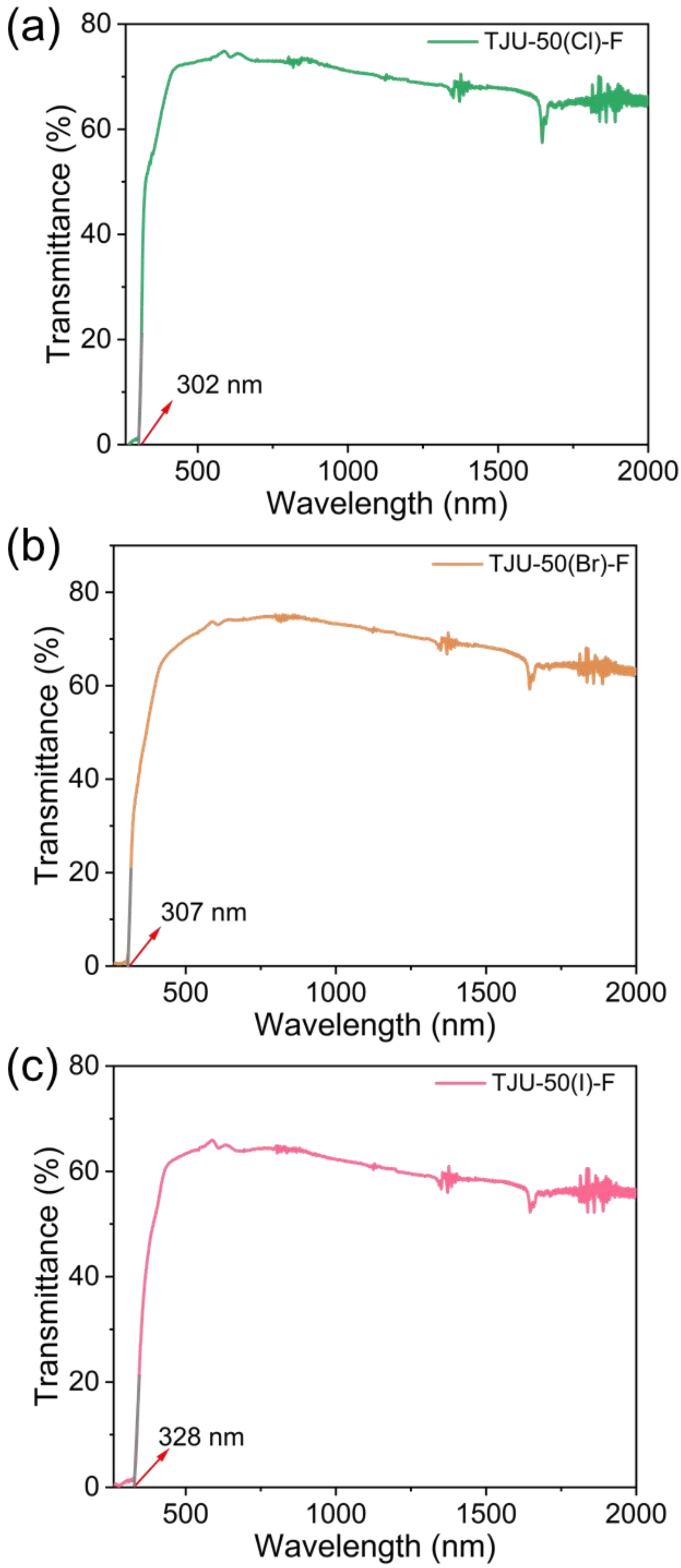

Fig. S17 UV-vis-NIR transmittance spectra of TJU-50(Cl)-F (a), TJU-50(Br)-F, (b) TJU-50(I)-F (c).

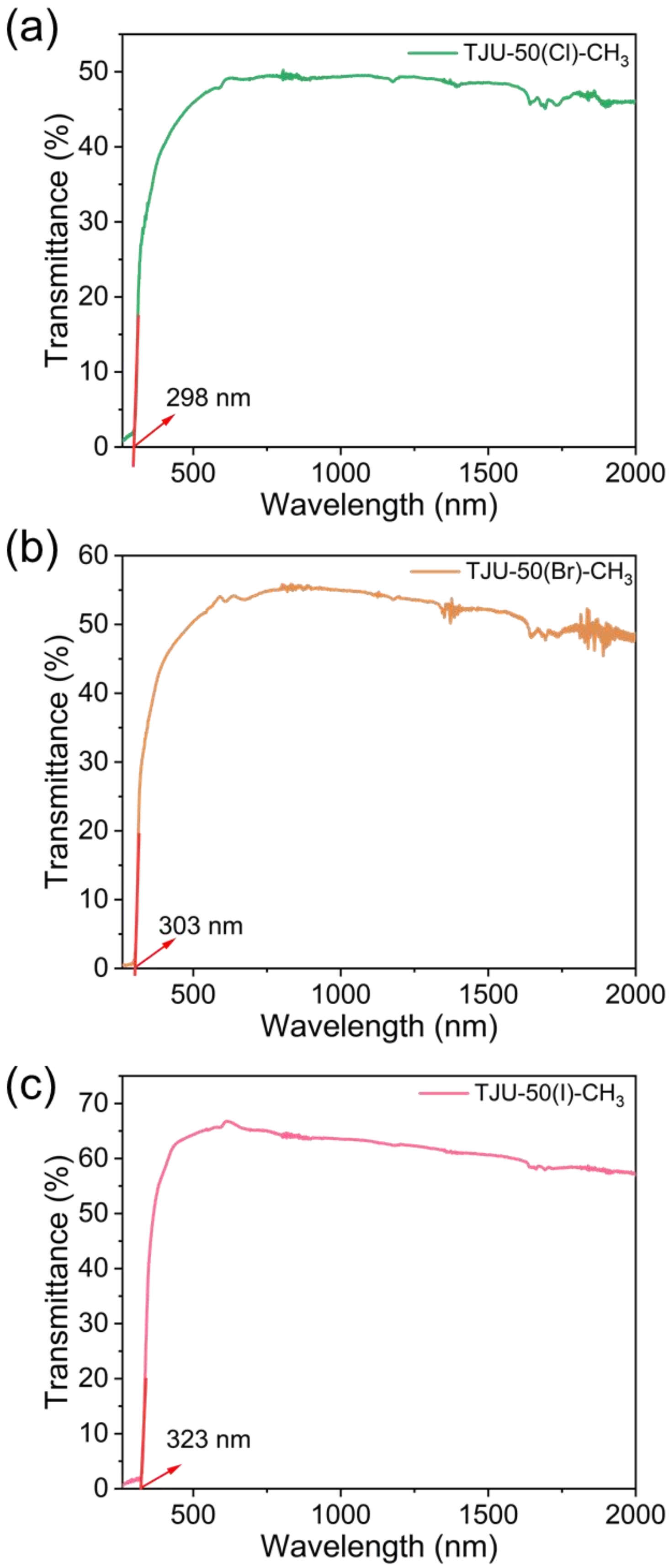

Fig. S18 UV-vis-NIR transmittance spectra of TJU-50(Cl)-CH_3 (a), TJU-50(Br)-CH_3 (b), TJU-50(I)-CH_3 (c).

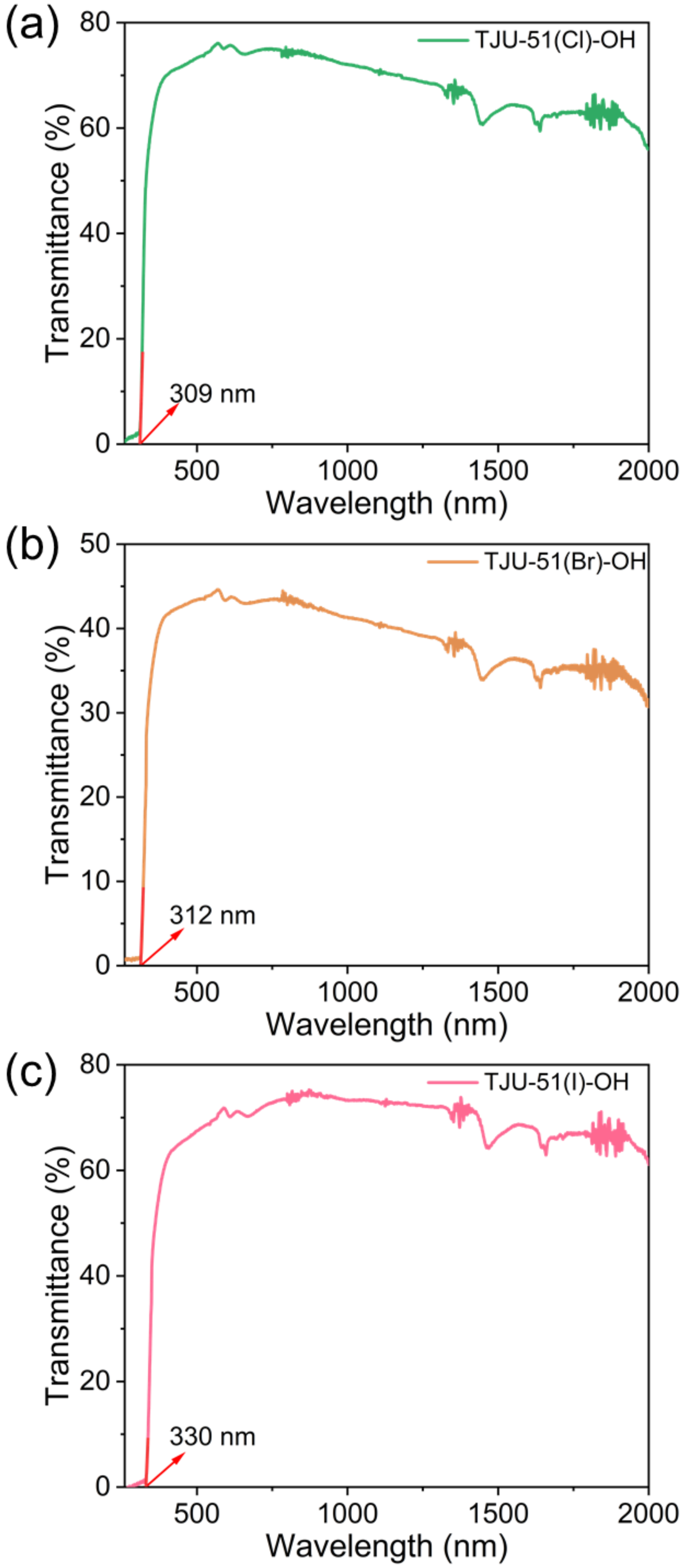

Fig. S19 UV-vis-NIR transmittance spectra of TJU-51(Cl)-OH(a), TJU-51(Br)-OH, (b) TJU-51(I)-OH (c).

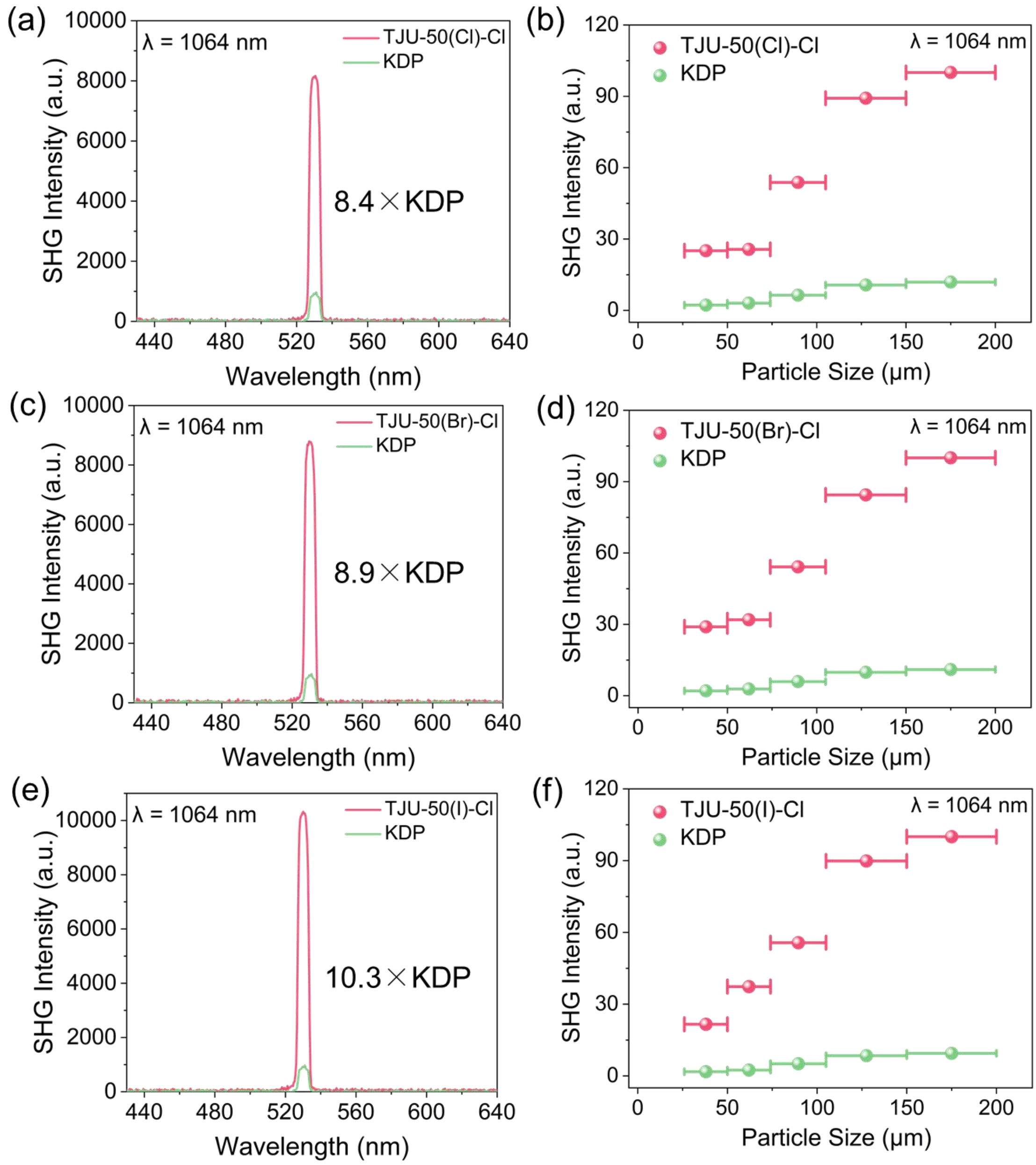

Fig. S20 (a, c, e) Oscilloscope traces of the SHG signals for powders of TJU-50(Cl)-Cl (a), TJU-50(Br)-Cl (c) and TJU-50(I)-Cl (e) (150-200 μm) with 1064 nm laser radiation. KDP was used as the reference for the SHG measurements at 1064 nm. (b, d, f) Phase-matching curves of TJU-50(Cl)-Cl (b), TJU-50(Br)-Cl (d), TJU-50(I)-Cl (f) and KDP with 1064 nm laser radiation.

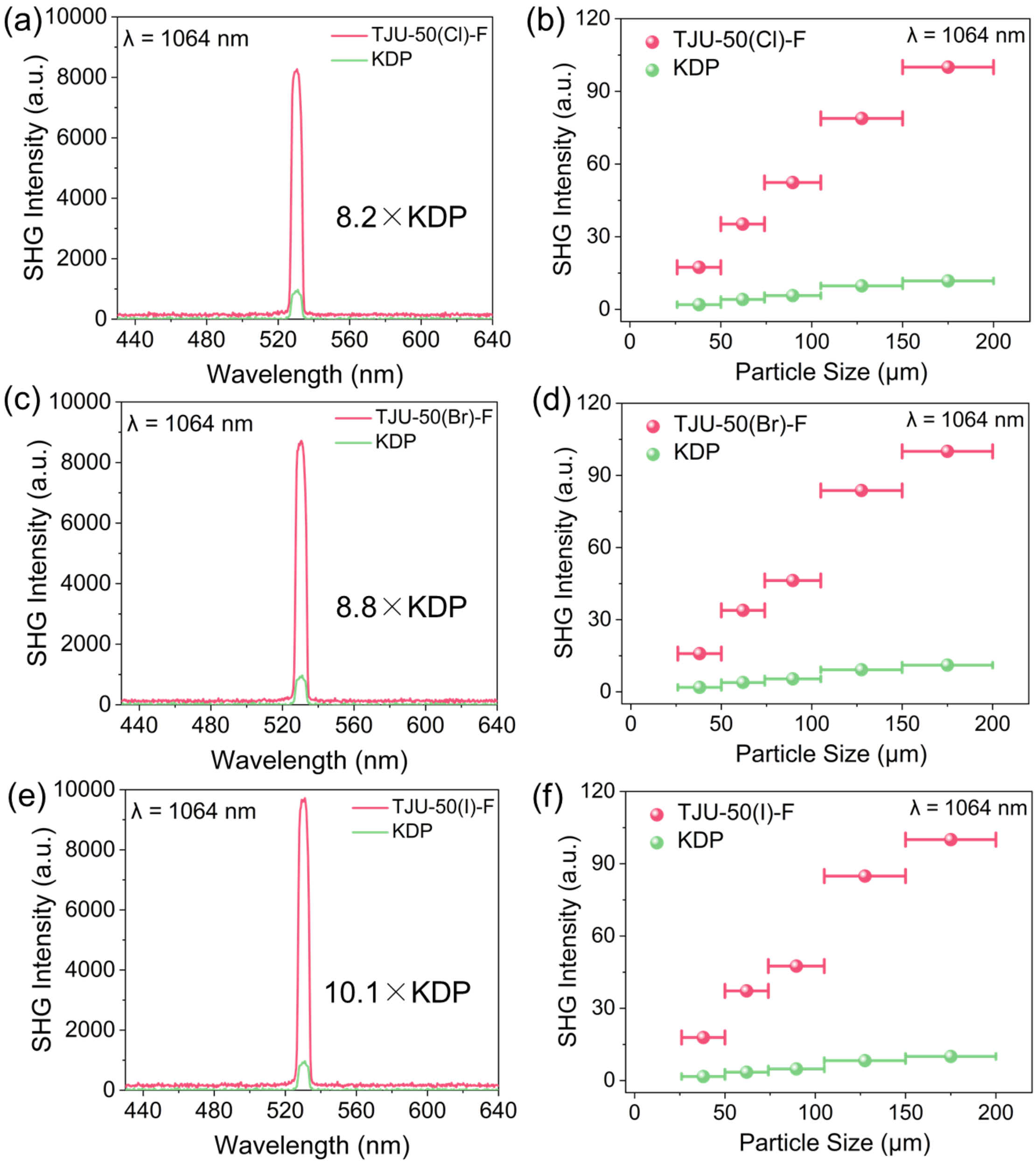

Fig. S21 (a, c, e) Oscilloscope traces of the SHG signals for powders of TJU-50(Cl)-F (a), TJU-50(Br)-F (c) and TJU-50(I)-F (e) (150-200 μm) with 1064 nm laser radiation. KDP was used as the reference for the SHG measurements at 1064 nm. (b, d, f) Phase-matching curves of TJU-50(Cl)-F (b), TJU-50(Br)-F (d), TJU-50(I)-F (f) and KDP with 1064 nm laser radiation.

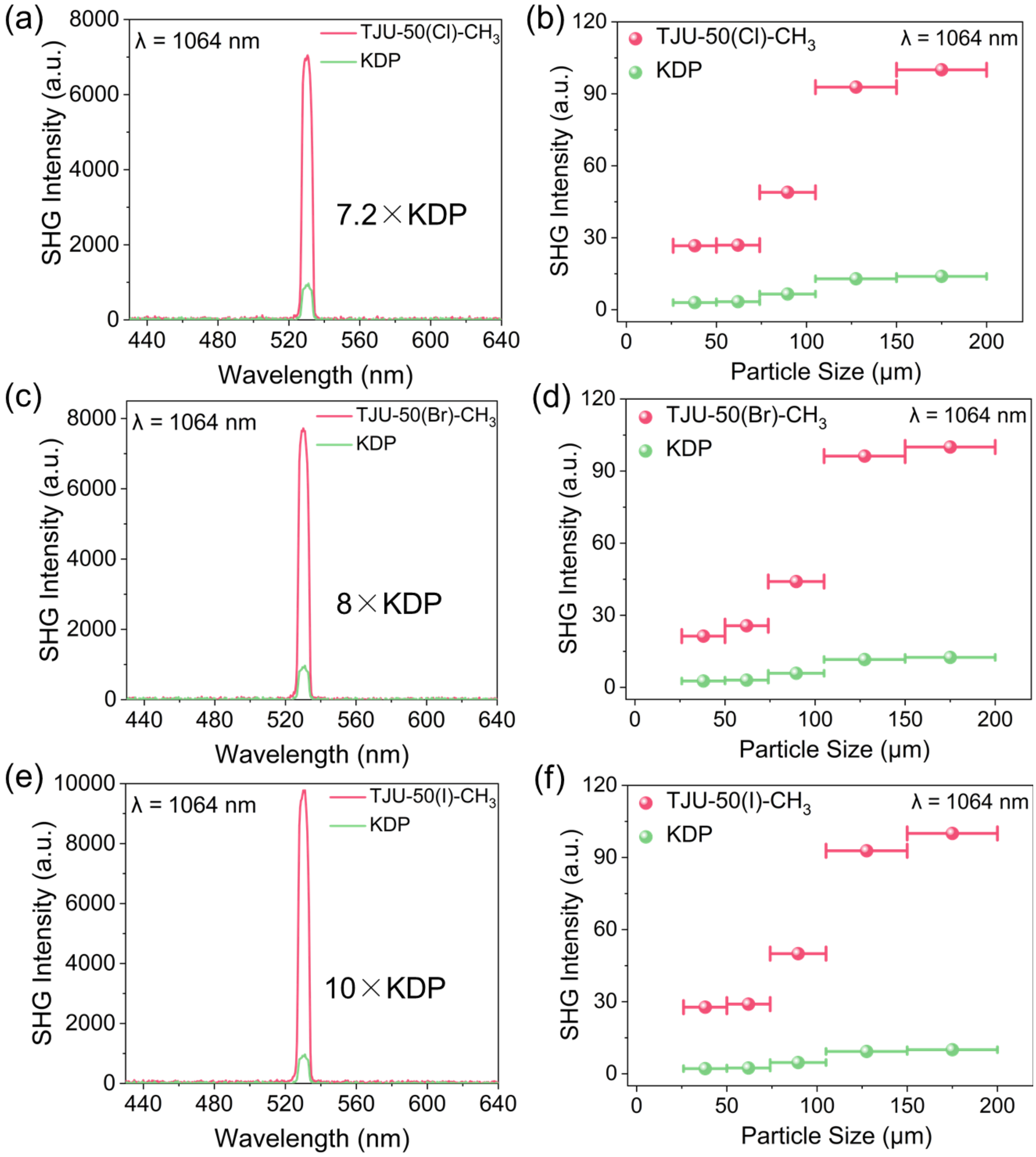

Fig. S22 (a, c, e) Oscilloscope traces of the SHG signals for powders of TJU-50(Cl)-CH_3 (a), TJU-50(Br)-CH_3 (c) and TJU-50(I)-CH_3 (e) (150-200 μm) with 1064 nm laser radiation. KDP was used as the reference for the SHG measurements at 1064 nm. (b, d, f) Phase-matching curves of TJU-50(Cl)-CH_3 (b), TJU-50(Br)-CH_3 (d), TJU-50(I)-CH_3 (f) and KDP with 1064 nm laser radiation.

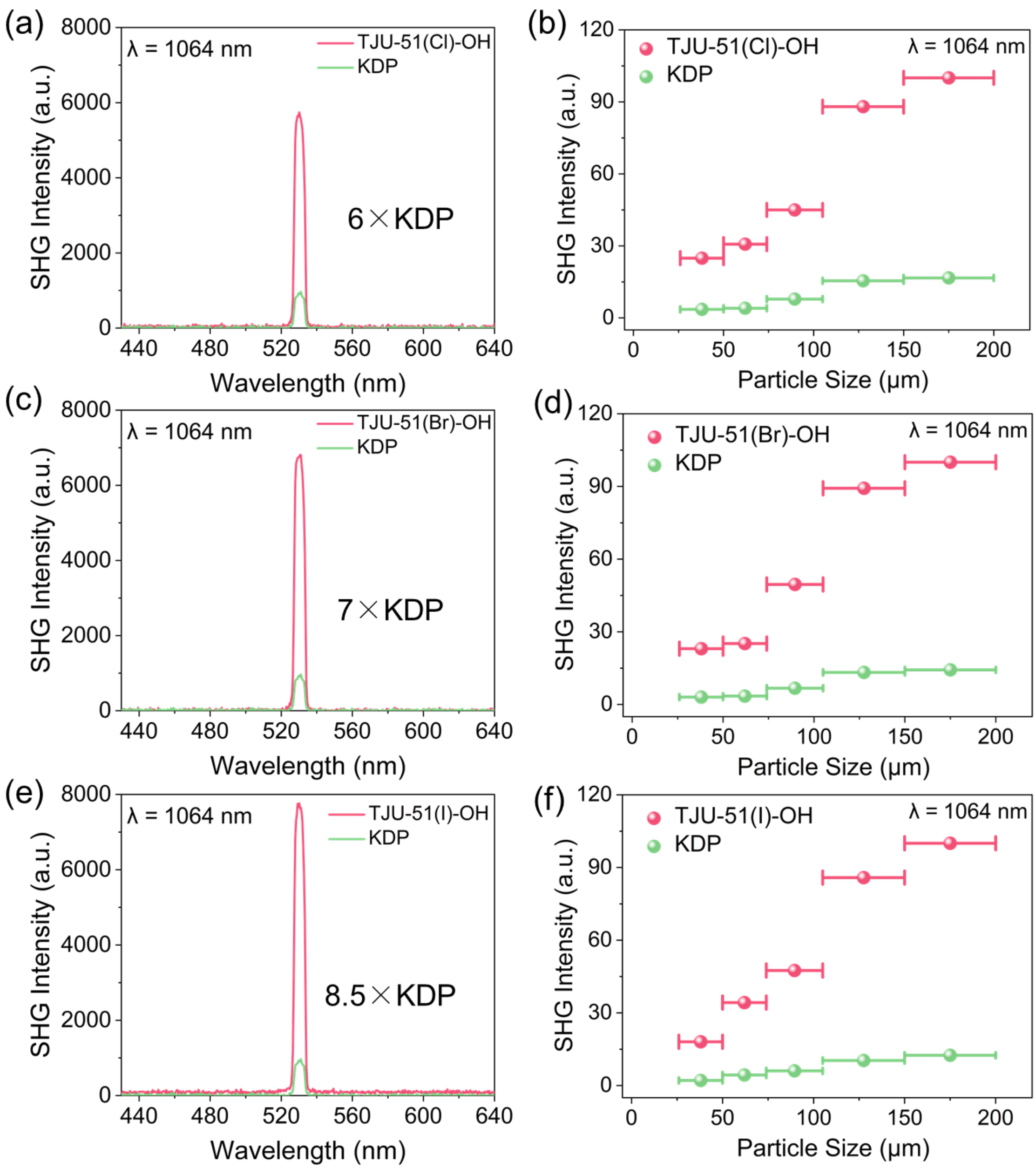

Fig. S23 (a, c, e) Oscilloscope traces of the SHG signals for powders of TJU-51(Cl)-OH (a), TJU-51(Br)-OH (c) and TJU-51(I)-OH (e) (150-200 μm) with 1064 nm laser radiation. KDP was used as the reference for the SHG measurements at 1064 nm. (b, d, f) Phase-matching curves of TJU-51(Cl)-OH (b), TJU-51(Br)-OH (d), TJU-51(I)-OH (f) and KDP with 1064 nm laser radiation.

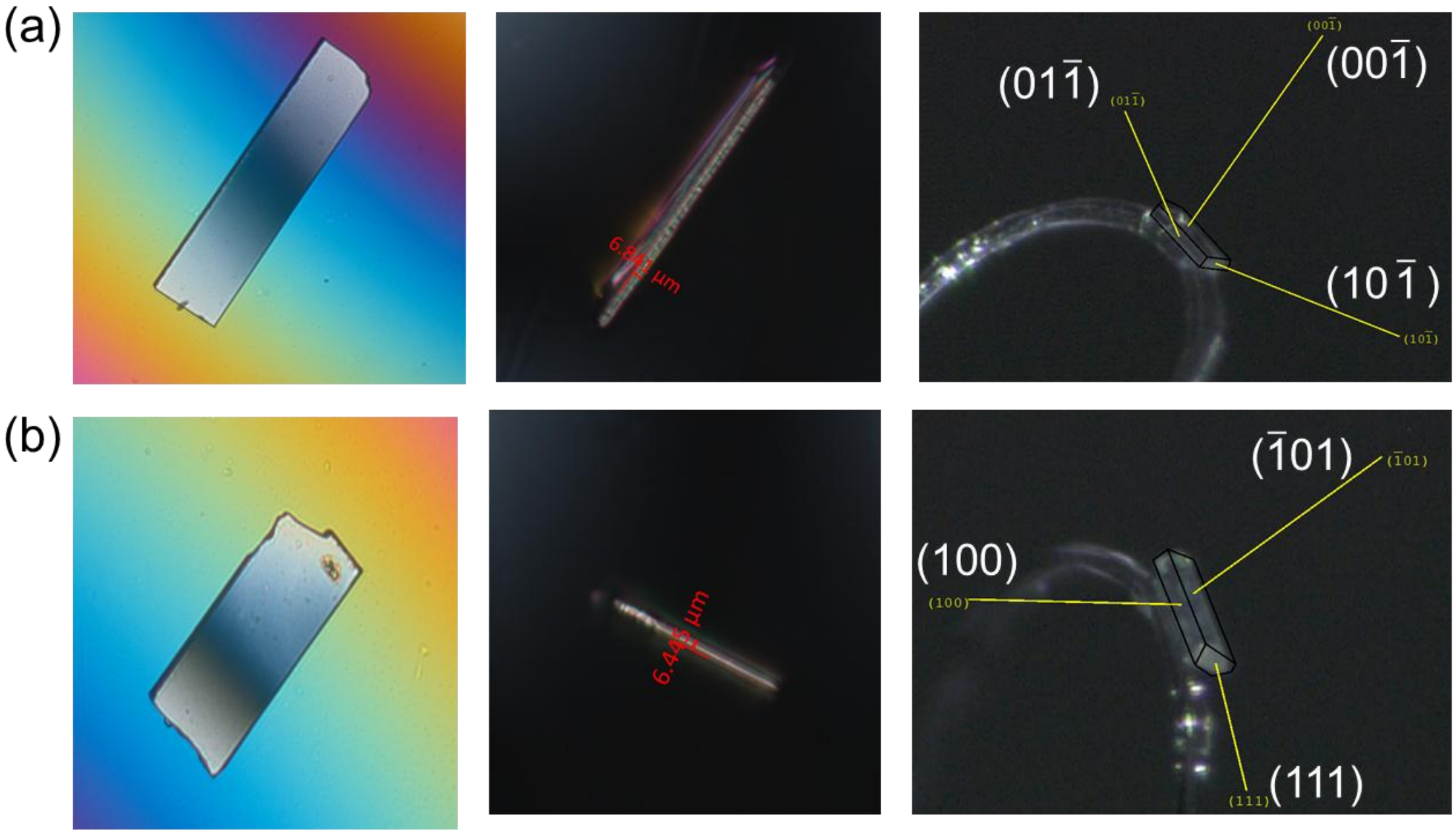

Fig. S24 (00$\bar{1}$) and ($\bar{1}$01) crystal plates of TJU-50(Cl)-Br (a) and TJU-50(Br)-Br (b) achieving extinction, and corresponding thickness of (01$\bar{1}$) and (100) for birefringence measurements. Determination of the crystal orientation and Miller indices.

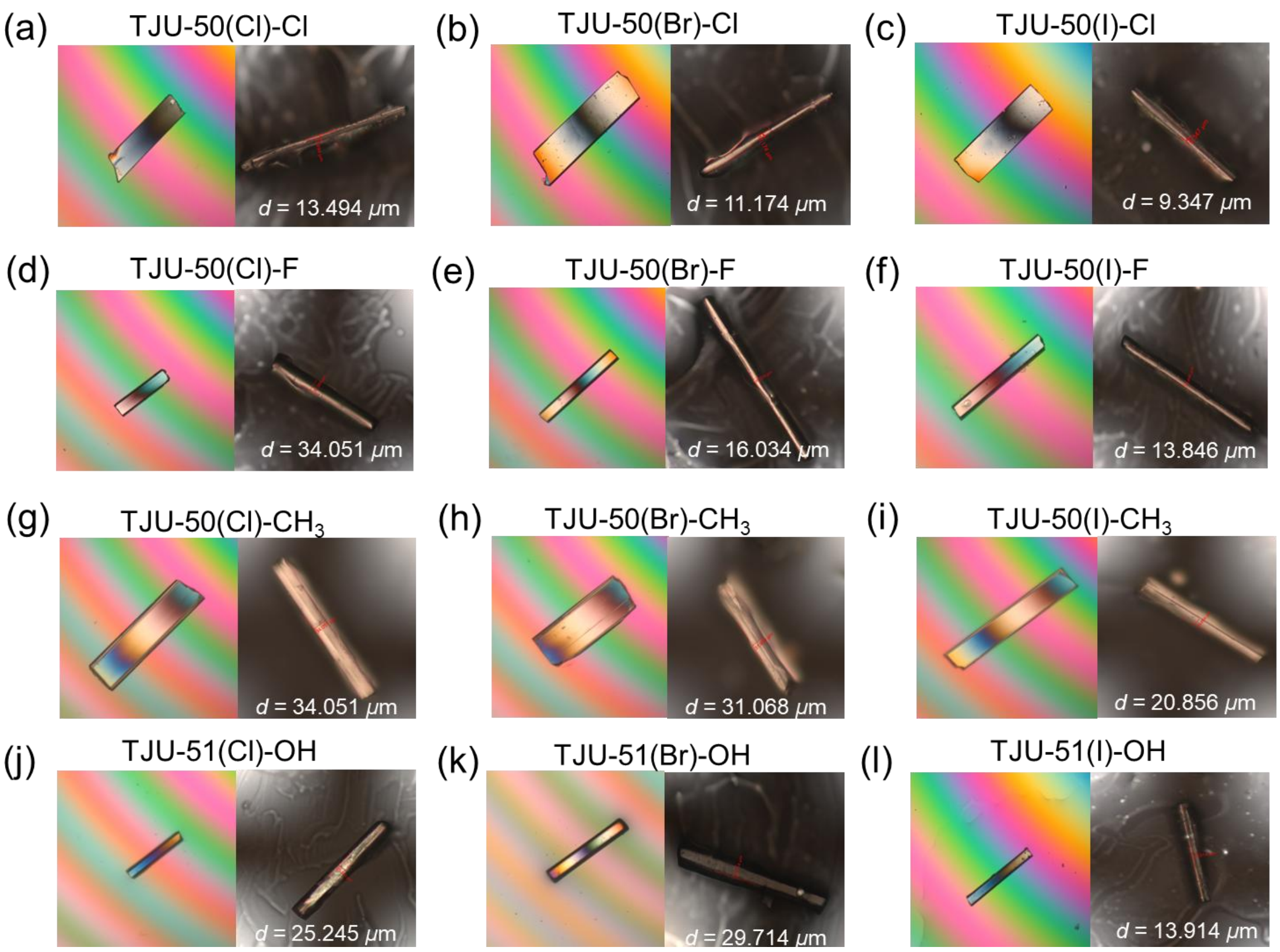

Fig. S25 Birefringence measurements of TJU-50(Cl)-Cl (a), TJU-50(Br)-Cl (b), TJU-50(I)-Cl (c); TJU-50(Cl)-F (d), TJU-50(Br)-F (e), TJU-50(I)-F (f); TJU-50(Cl)-CH_3 (g), TJU-50(Br)-CH_3 (g), TJU-50(I)-CH_3 (i) and TJU-51(Cl)-OH (j), TJU-50(Br)-OH (k), TJU-50(I)-OH (l).

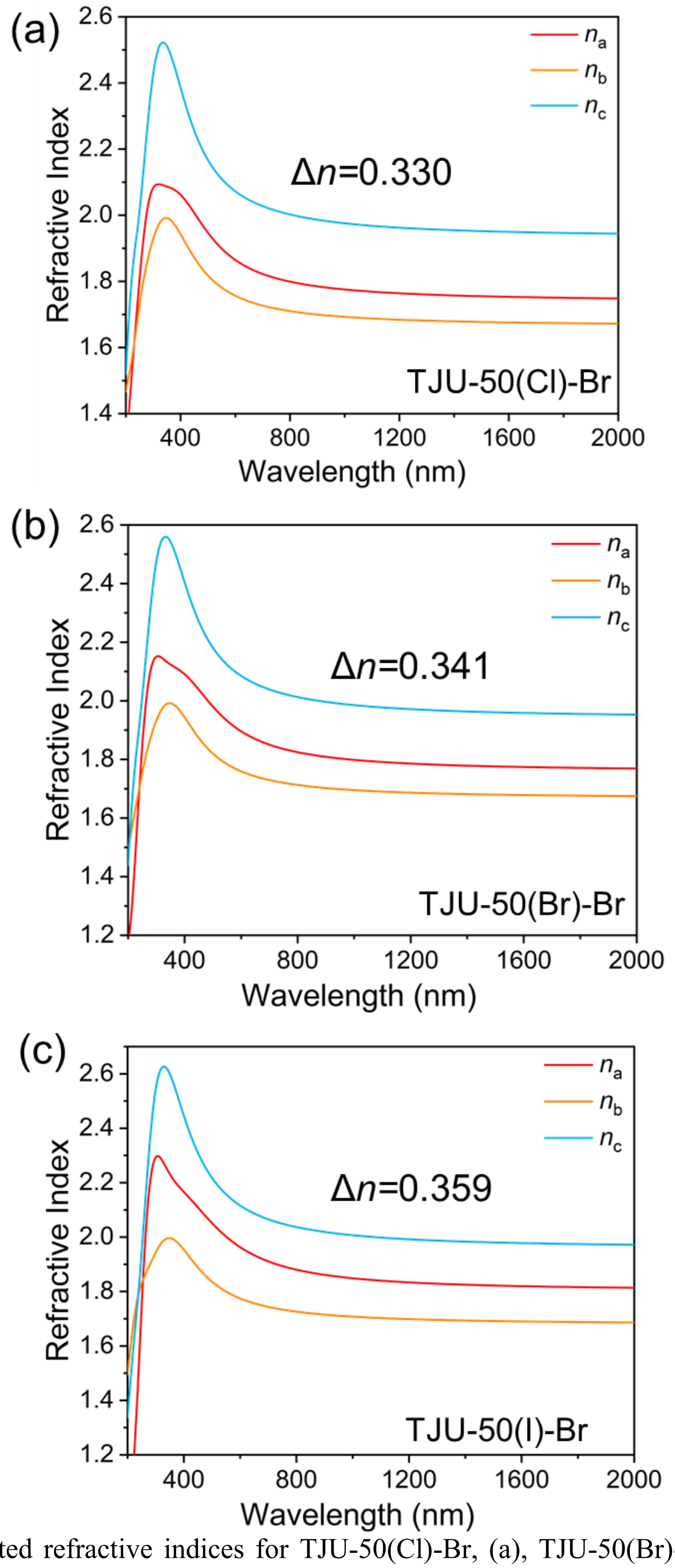

Fig. S26 Calculated refractive indices for TJU-50(Cl)-Br, (a), TJU-50(Br)-Br (b) and TJU-50(I)-Br (c).

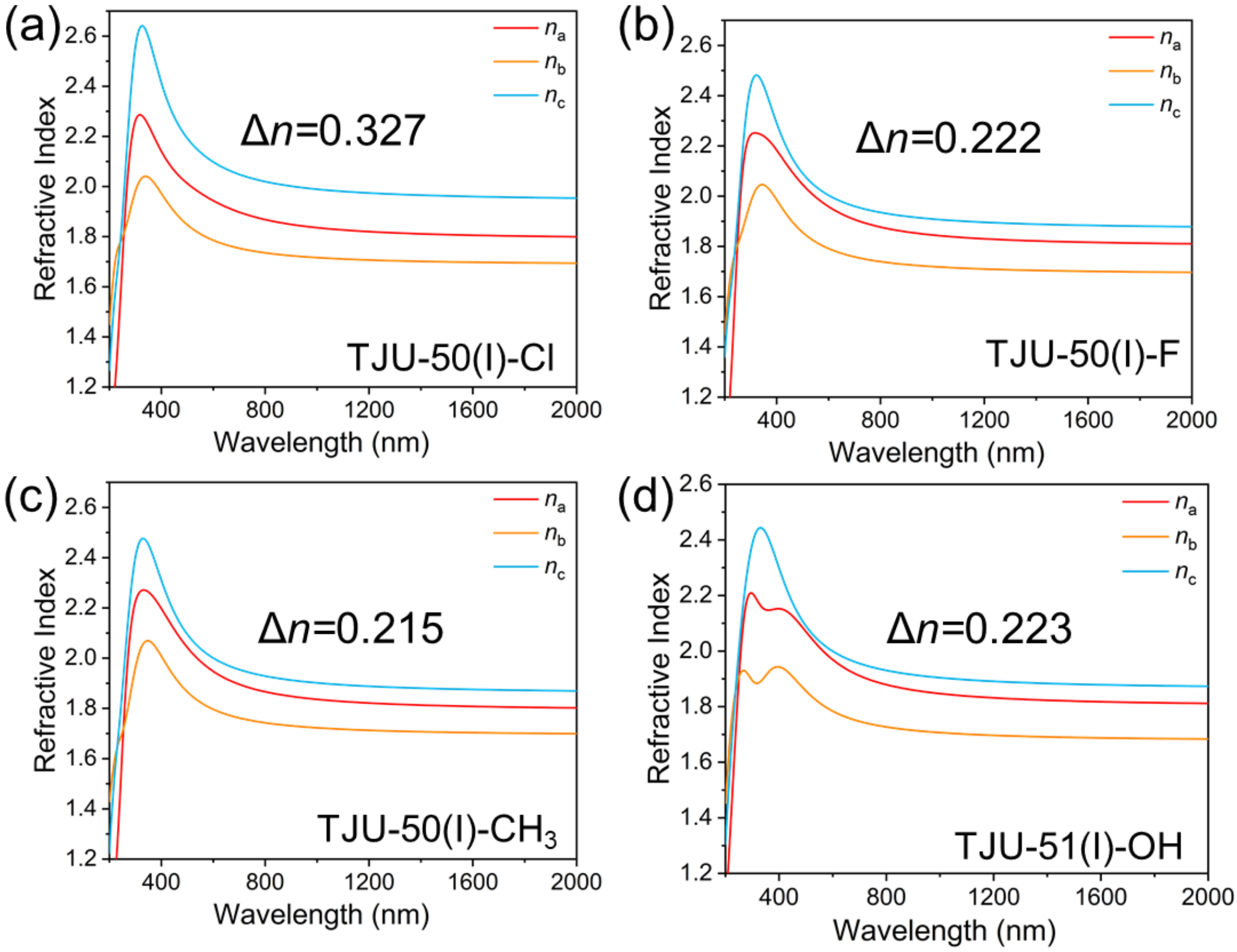

Fig. S27 Calculated refractive indices for TJU-50(I)-Cl (a), TJU-50(I)-F (b), TJU-50(I)-CH_3 (c) and TJU-51(I)-OH.

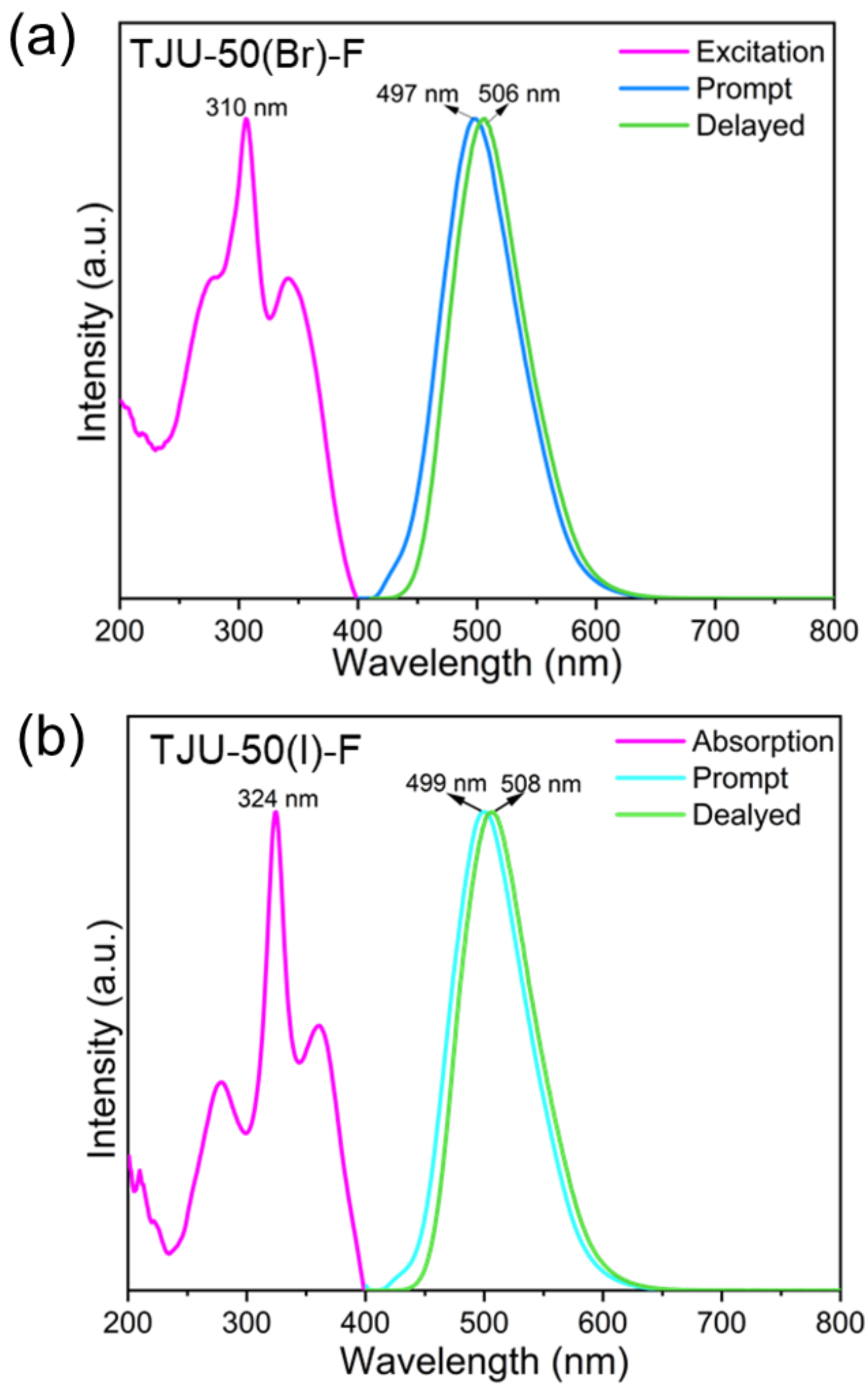

Fig. S28 Excited, prompt and delayed PL spectra of TJU-50(Br)-F (a) and TJU-50(I)-F (b).

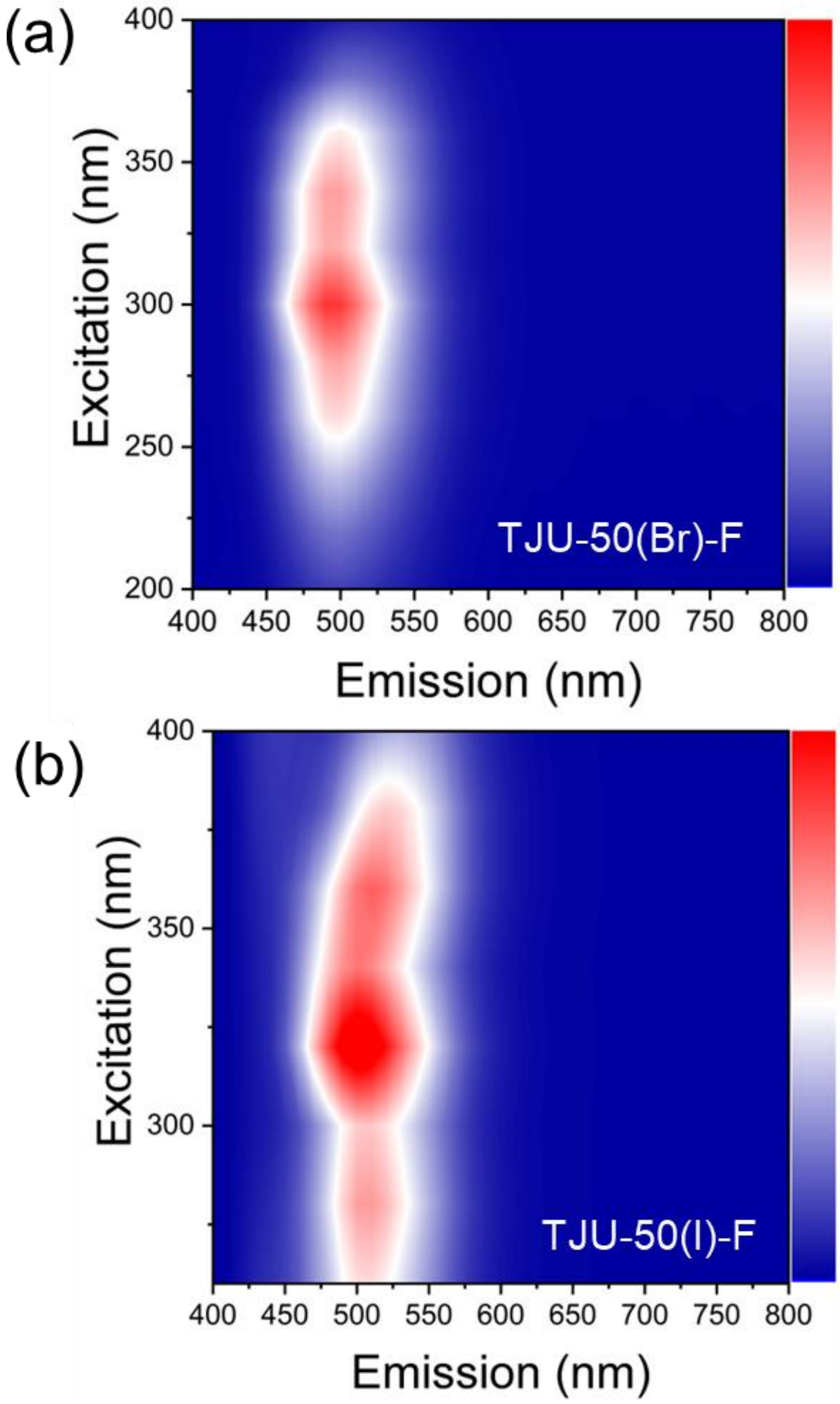

Fig. S29 The contour plot of 3D PL map in prompt-mode of TJU-50(Br)-F (a) and TJU-50(I)-F (b).

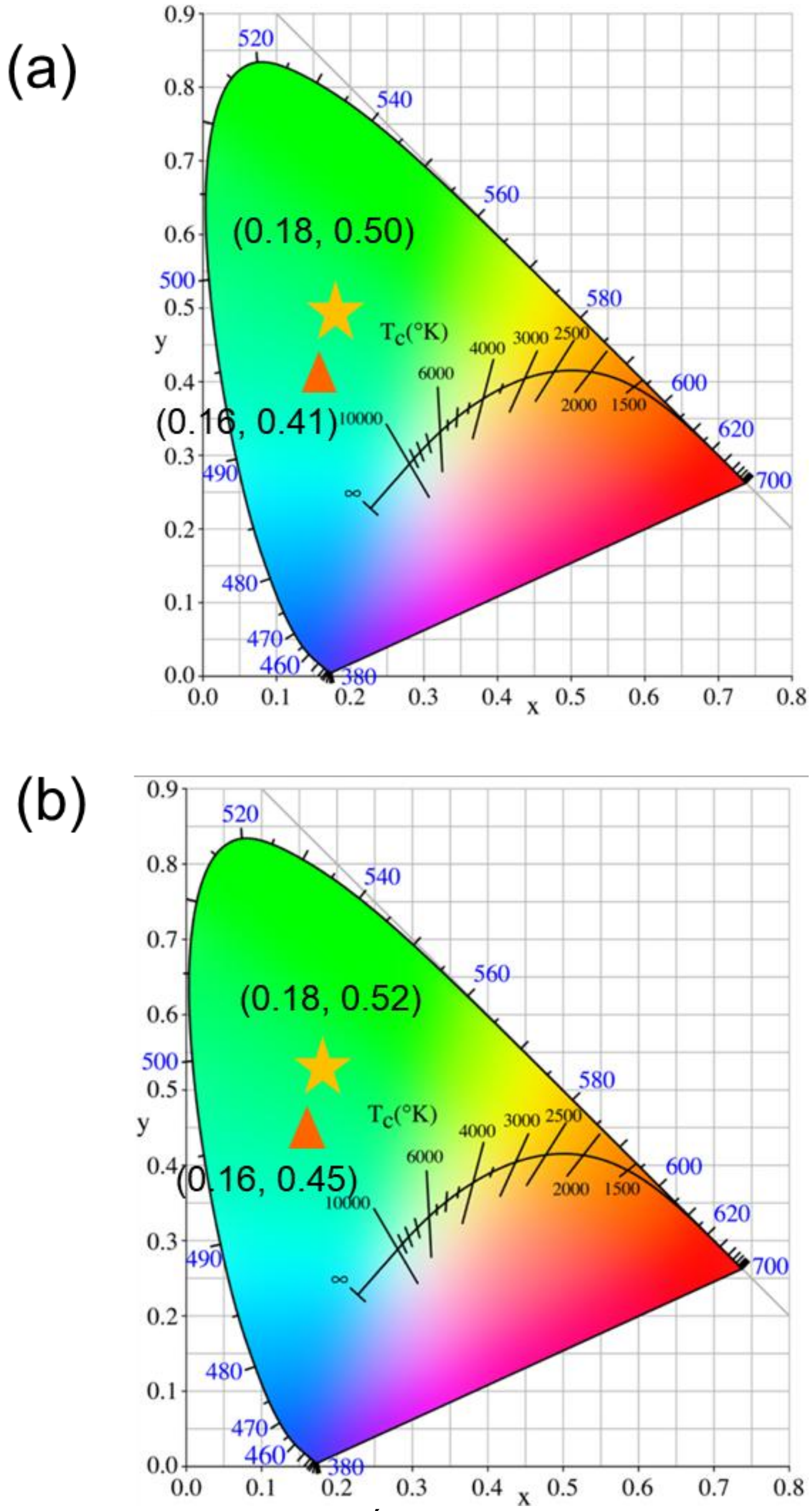

Fig. S30 CIE (Commission Internationale de l´Eclairage) coordinate diagram of the prompt PL emission (orange triangle) and green afterglow (yellow star) for TJU-50(Br)-F (a) and TJU-50(I)-F (b).

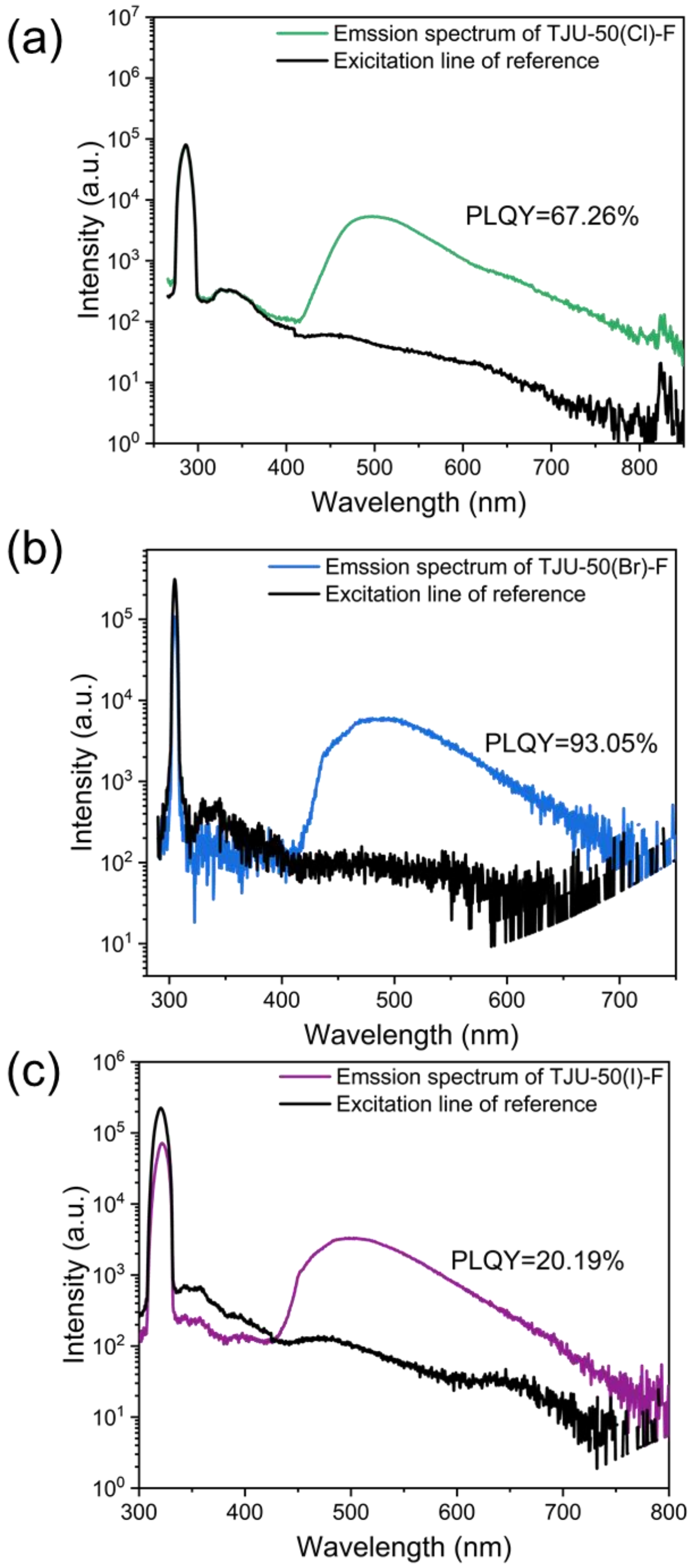

Fig. S31 PLQYs of TJU-50(Cl)-F(a), TJU-50(Br)-F(b) and TJU-50(I)-F(c) under prompt-mode.

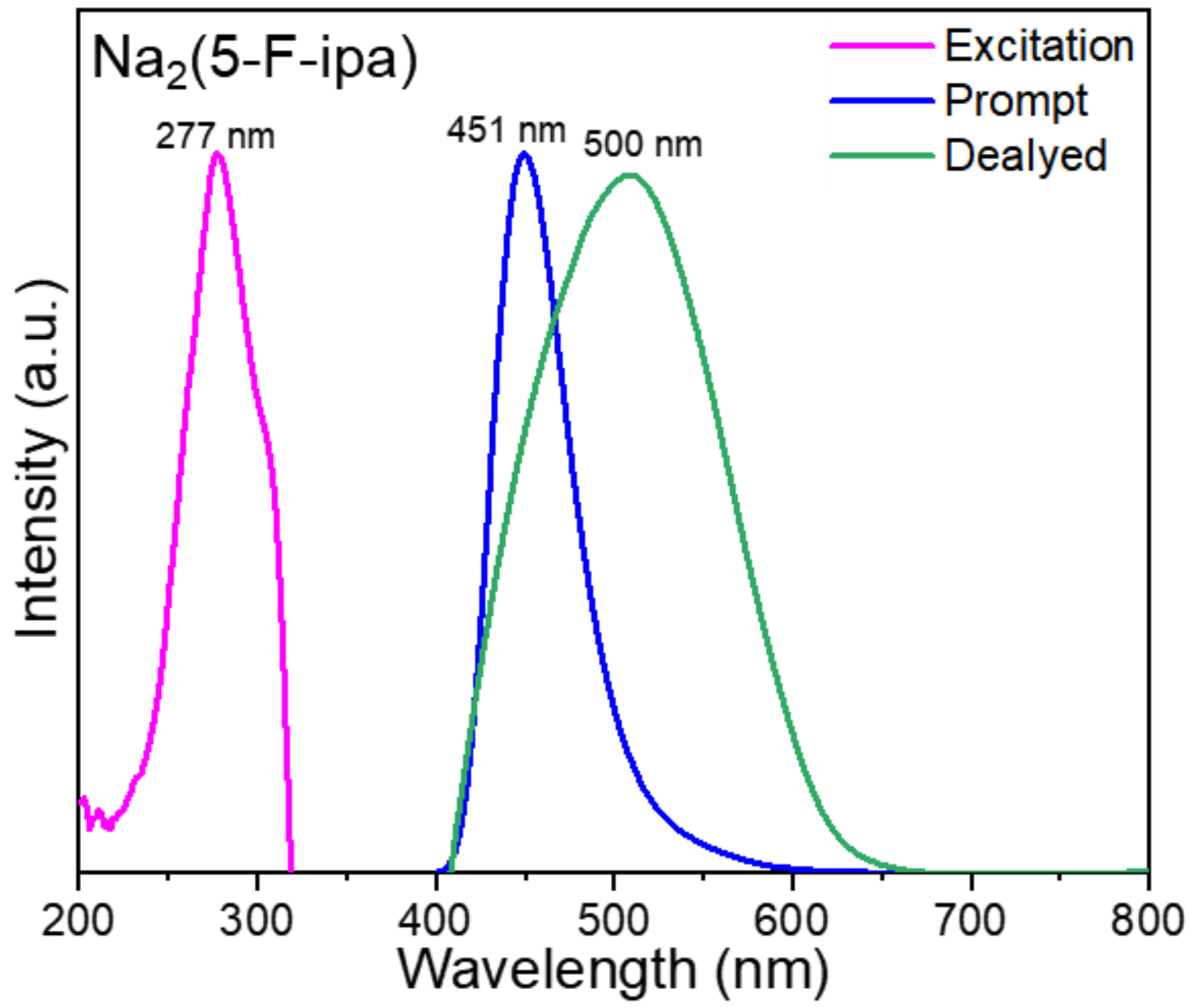

Fig. S32 Excited, prompt and delayed PL spectra of of Na_2(5-F-ipa).

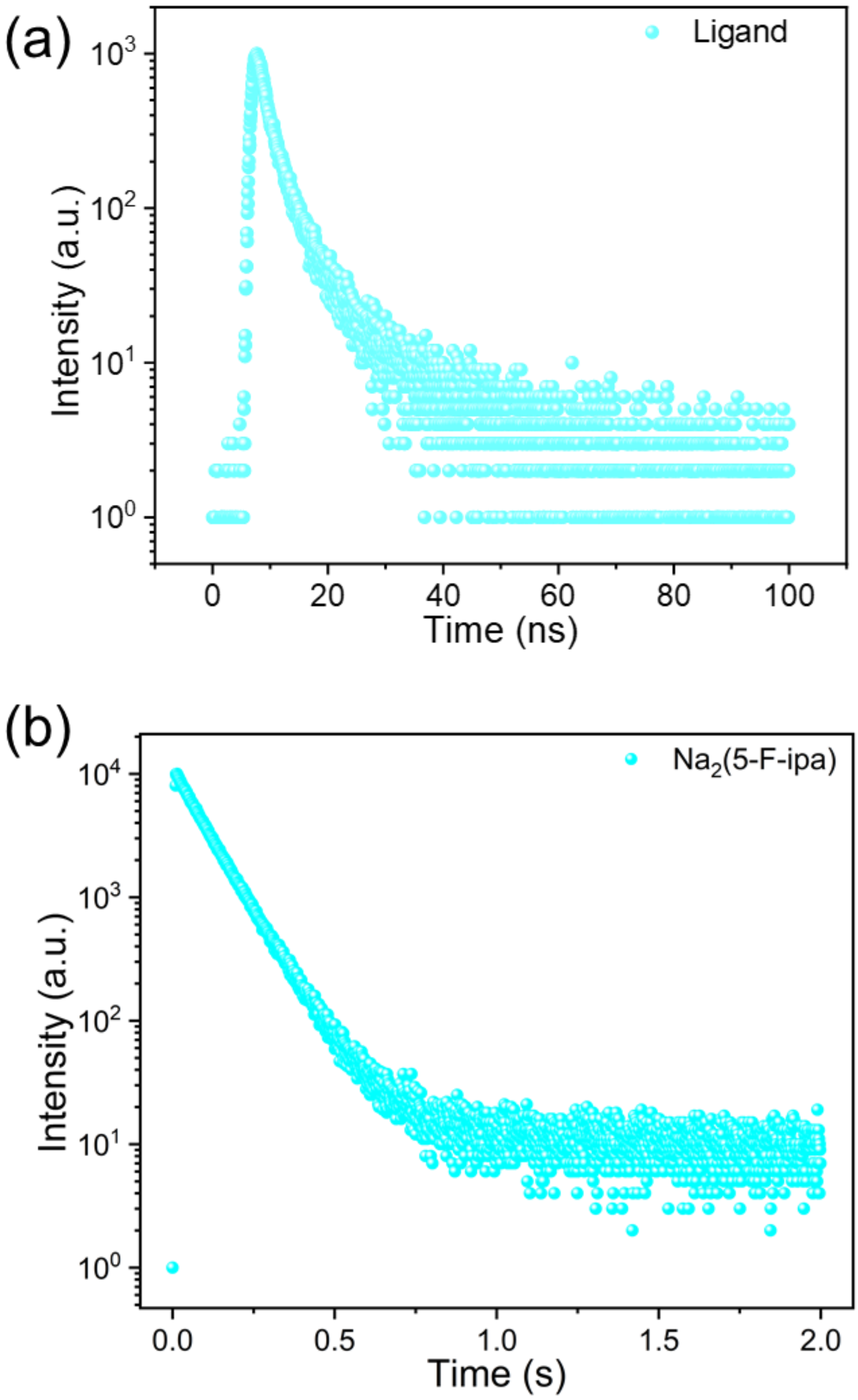

Fig. S33 The ns-scale prompt (a) and long-lived delayed (b) lifetime decay profiles of Na_2(5-F-ipa).

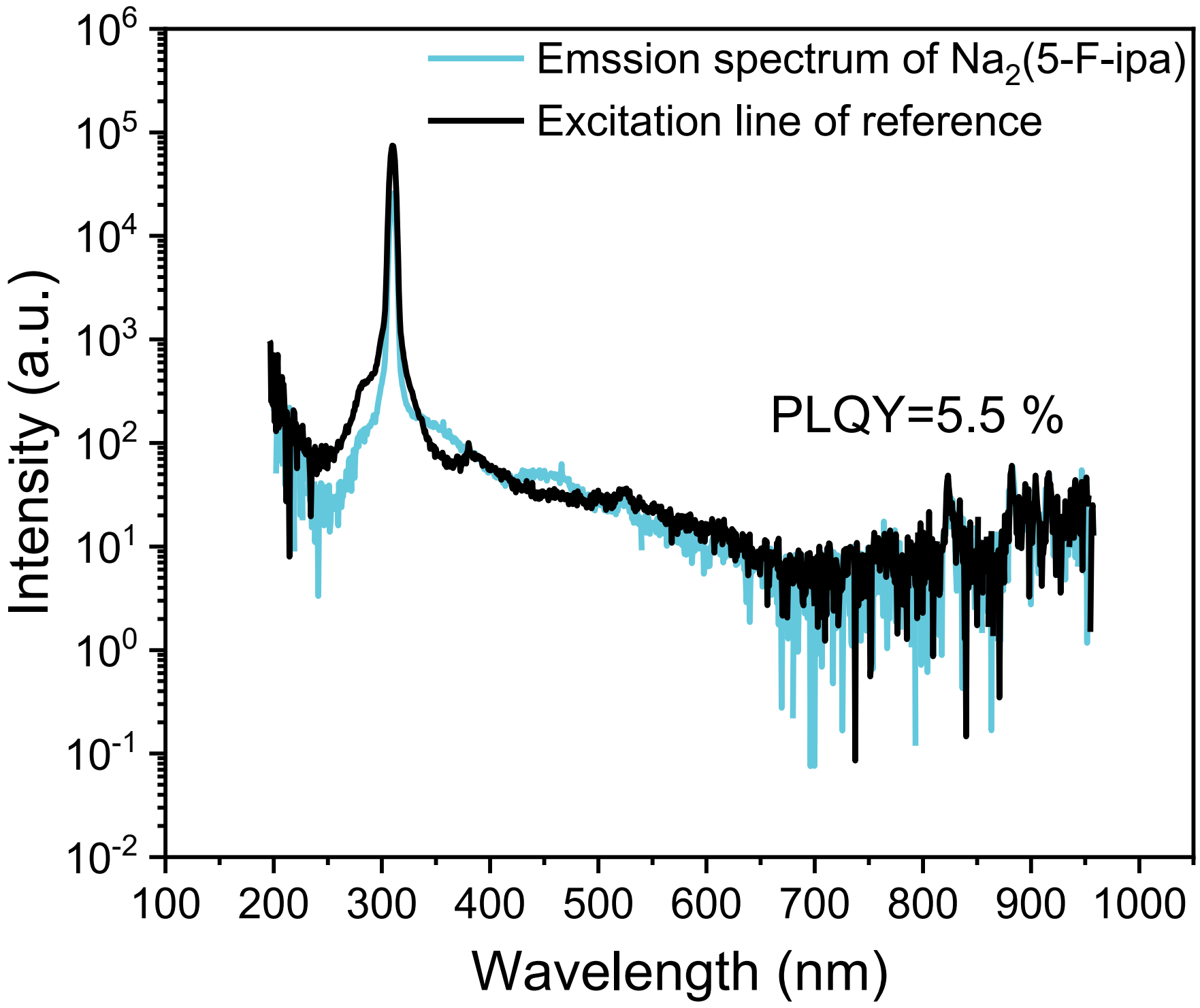

Fig. S34 PLQYs of TJU-50(Cl)-Cl.

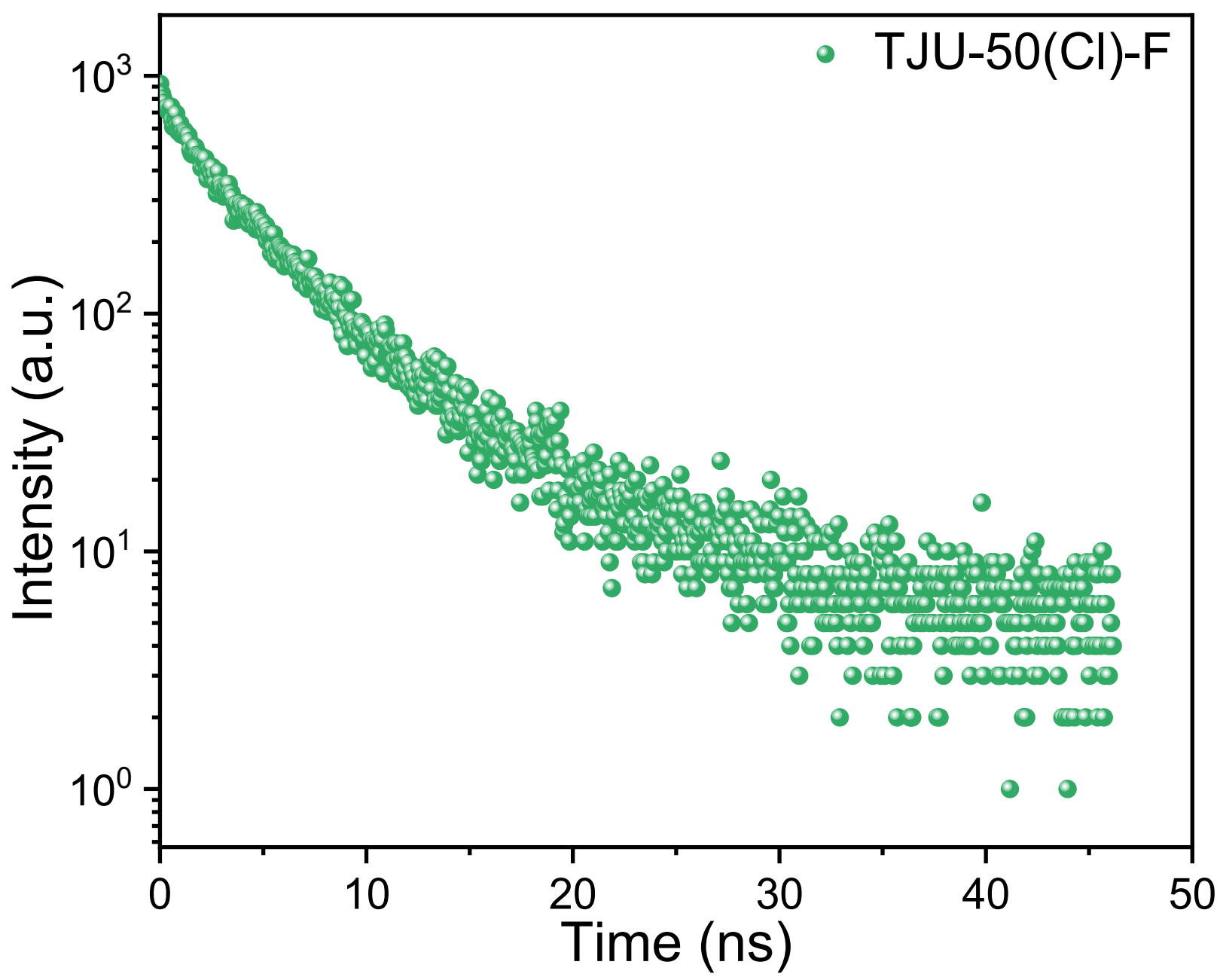

Fig. S35 The ns-scale prompt lifetime decay profile of TJU-50(Cl)-F.

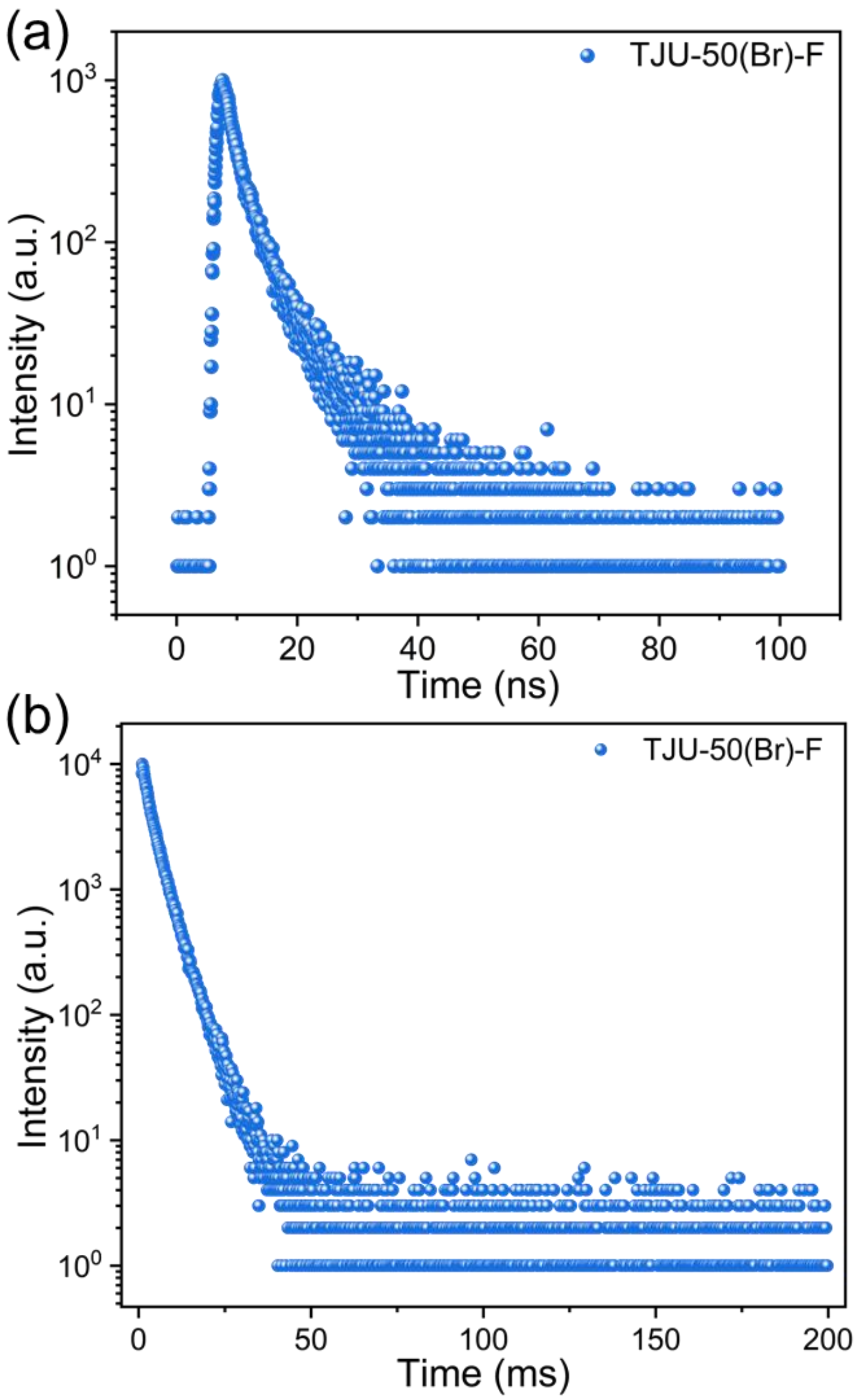

Fig. S36 The ns-scale prompt (a) and long-lived delayed (b) lifetime decay profiles of TJU-50(Br)-F.

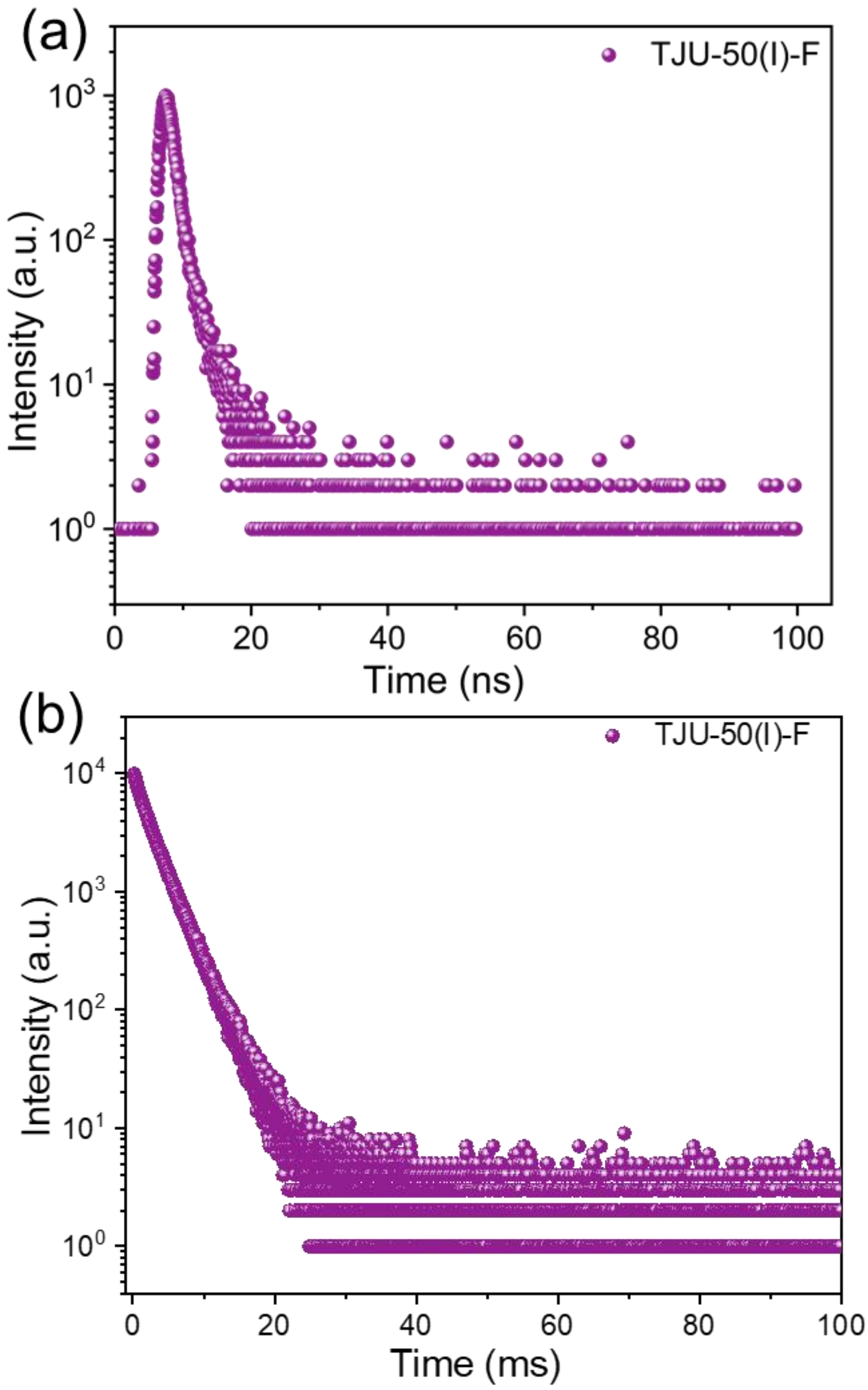

Fig. S37 The ns-scale prompt (a) and long-lived delayed (b) lifetime decay profiles of TJU-50(I)-F.

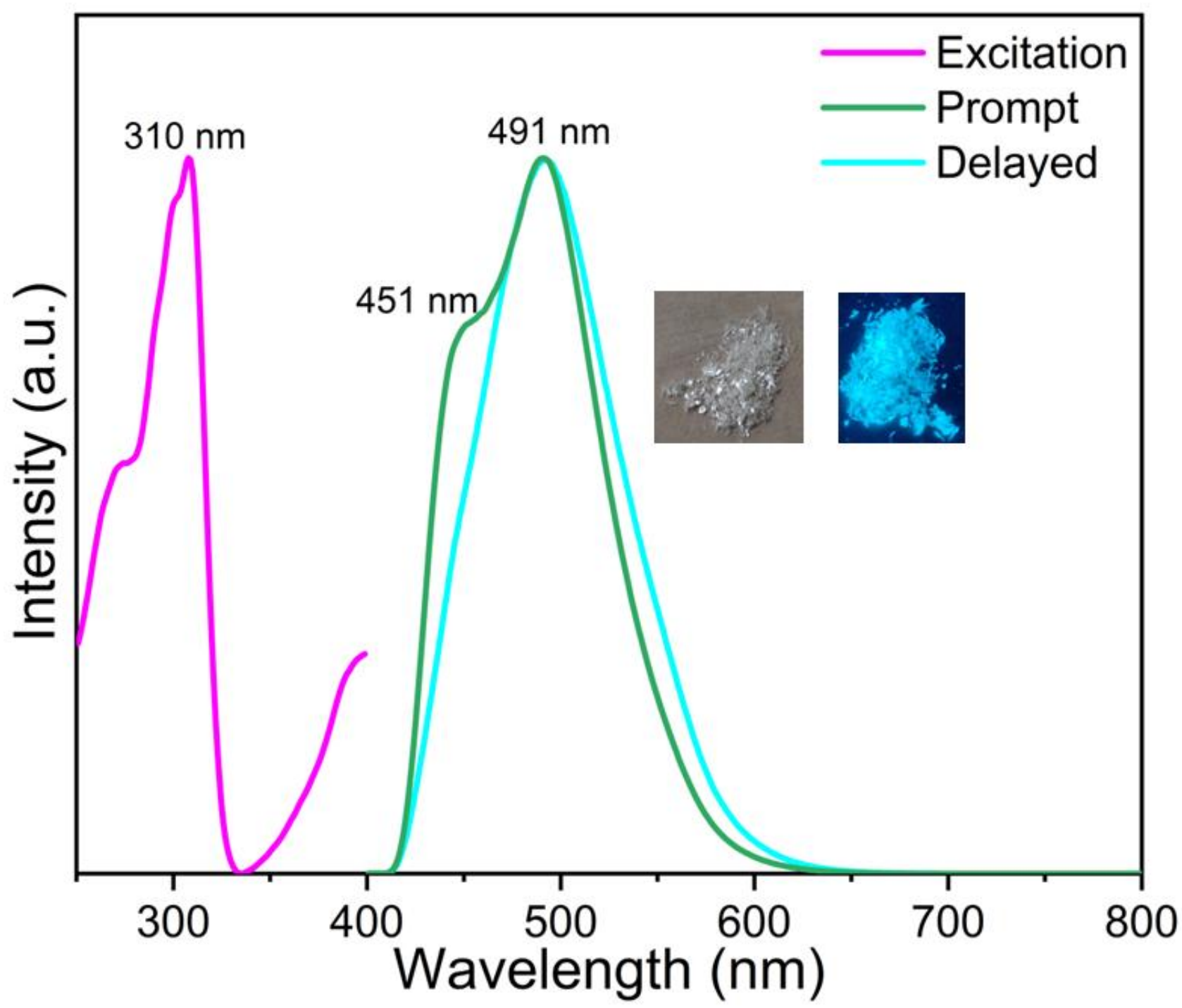

Fig. S38 Excited, prompt and delayed PL spectra of TJU-50(Cl)-Cl.

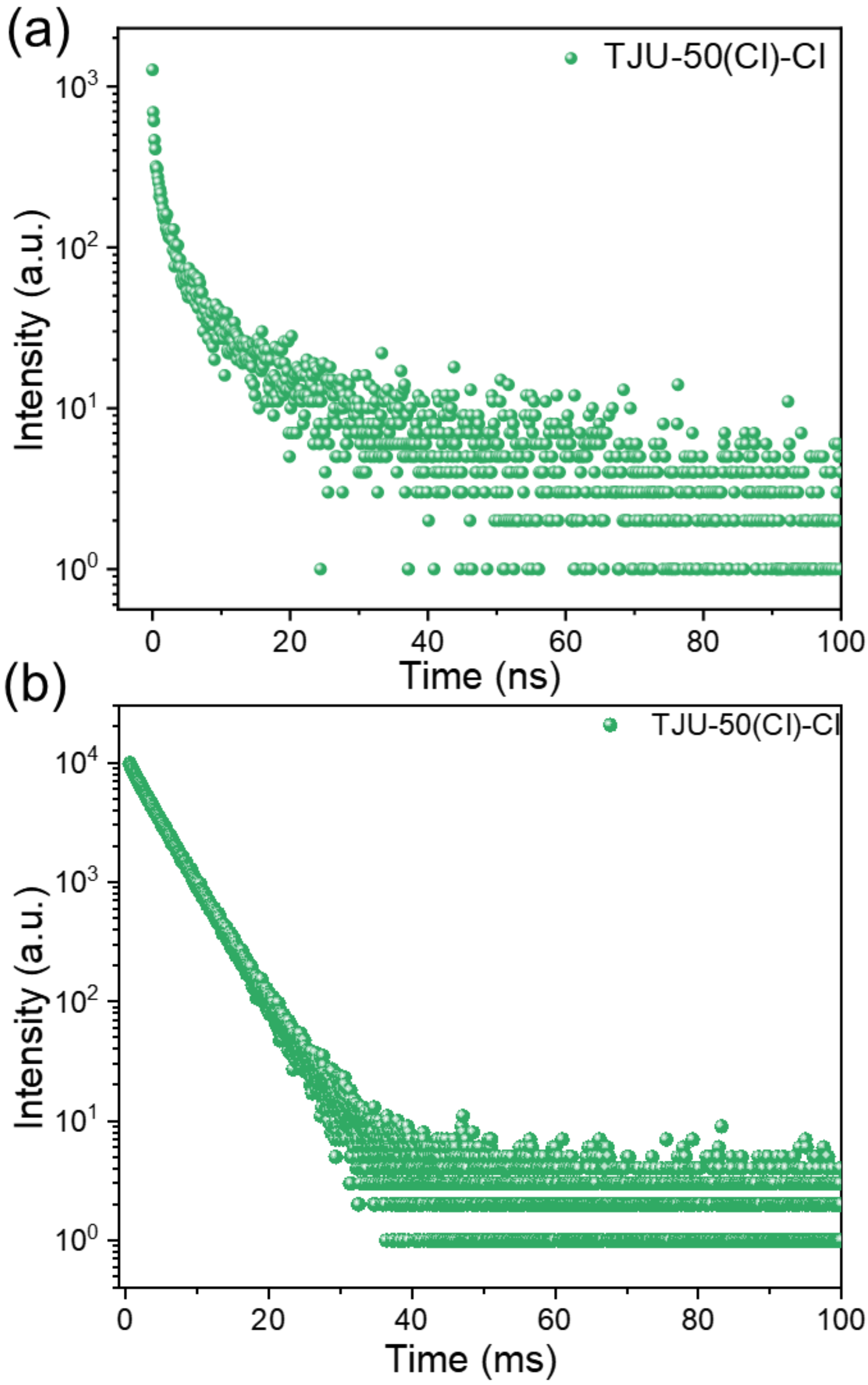

Fig. S39 The ns-scale prompt (a) and long-lived delayed (b) lifetime decay profiles of TJU-50(Cl)-Cl.

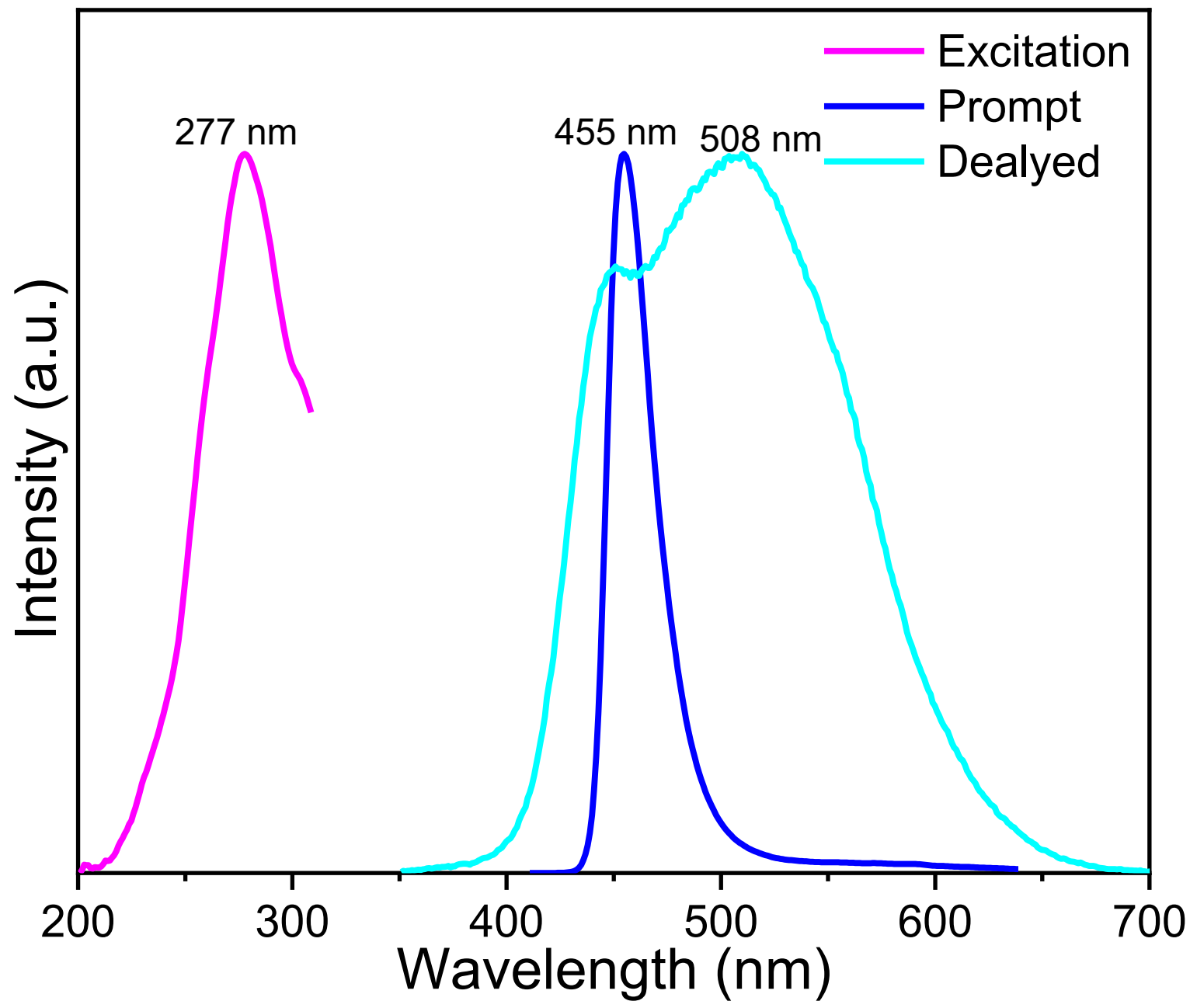

Fig. S40. Excited, prompt and delayed PL spectra of Na_2(5-Cl-ipa).

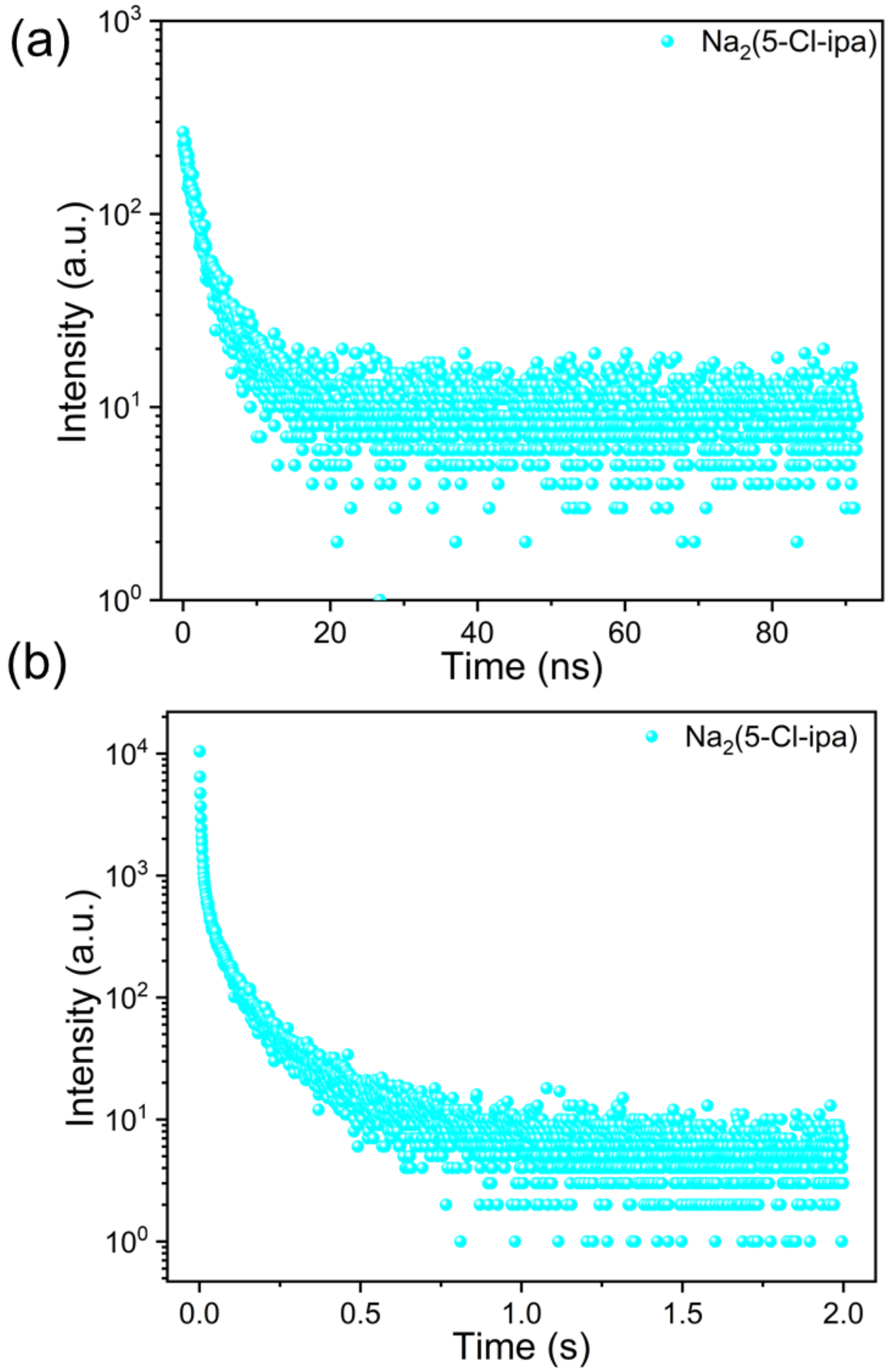

Fig. S41. The ns-scale prompt (a) and long-lived delayed (b) lifetime decay profiles of Na_2(5-Cl-ipa).

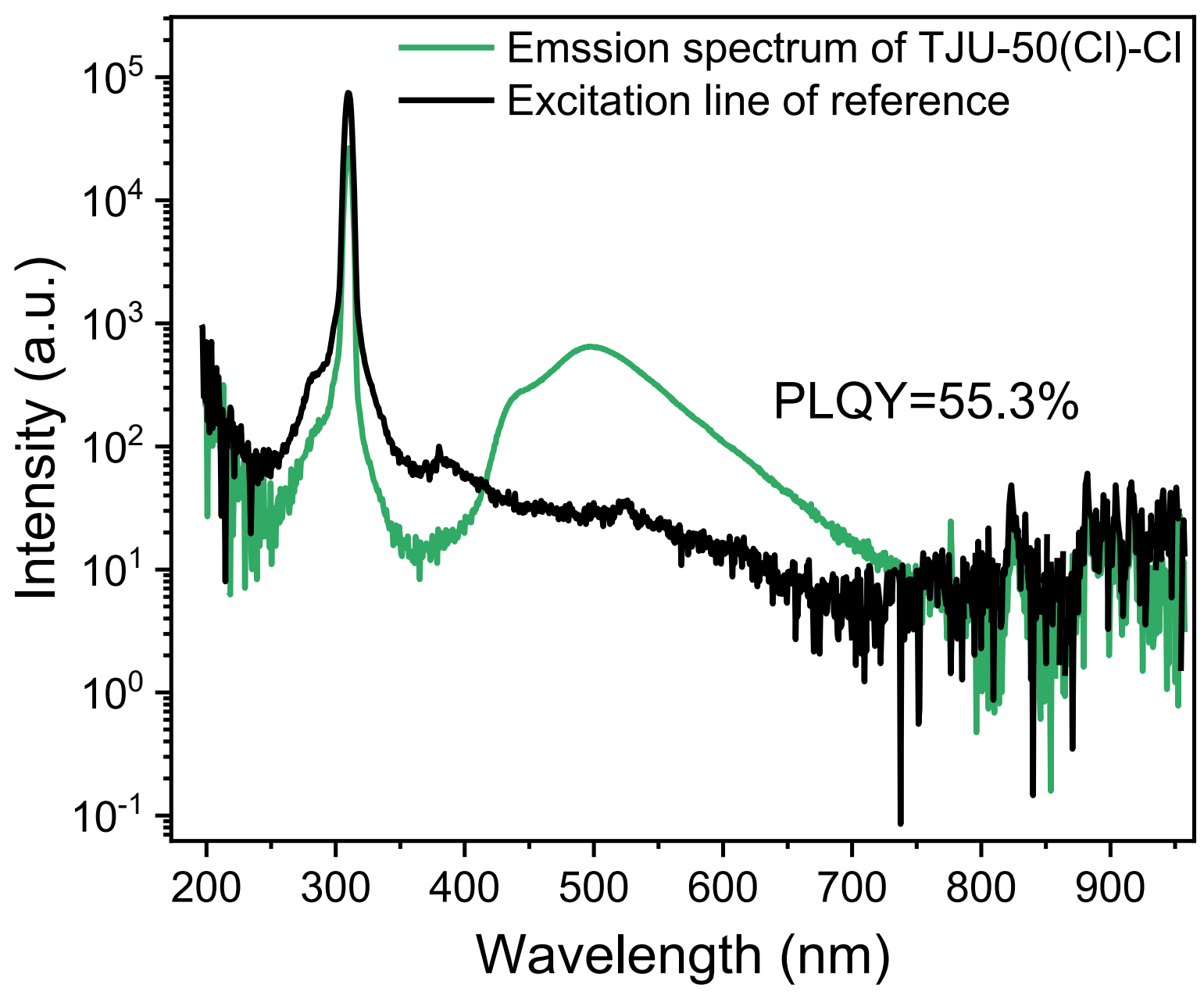

Fig. S42. PLQY of TJU-50(Cl)-Cl.

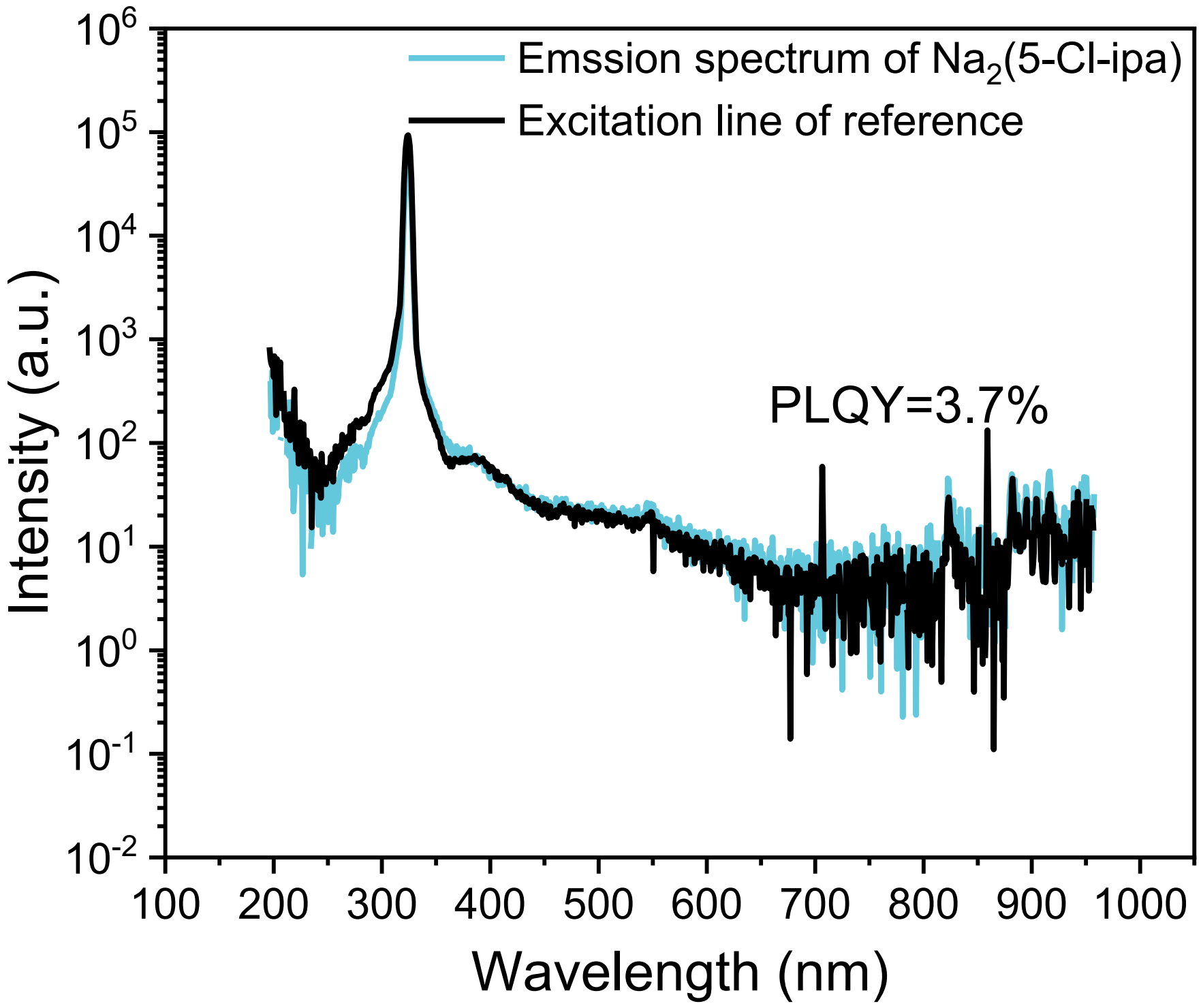

Fig. S43. PLQY of Na_2(5-Cl-ipa).

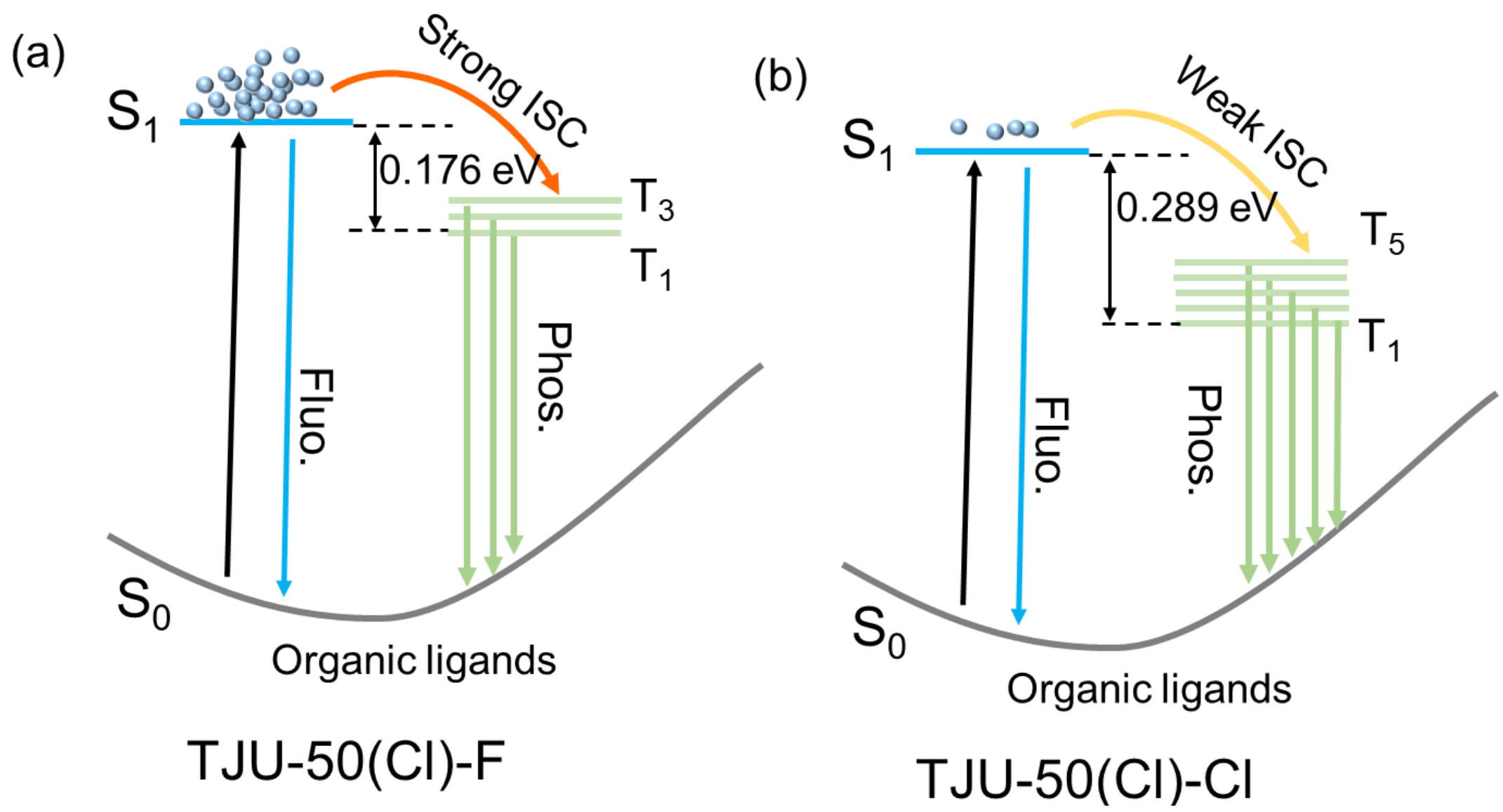

Fig. S44 Schematic illustration of the proposed photophysical processes for TJU-50(Cl)-F and TJU-50(Cl)-Cl.

Table S1. Crystal data and structure refinement for TJU-50(Cl)-Br, TJU-50(Br)-Br and TJU-50(I)-Br.

Identification code	TJU-50(Cl)-Br	TJU-50(Br)-Br	TJU-50(I)-Br
Empirical formula	$C_{18}H_{14}Br_2NO_8Pb_2Cl$	$C_{18}H_{14}Br_2NO_8Pb_2Br$	$C_{18}H_{14}Br_2NO_8Pb_2I$
Formula weight	981.95	1026.40	1073.40
Tem (K)	300	300	300
Crystal system	orthorhombic	orthorhombic	orthorhombic
Space group	*Ima*2	*Ima*2	*Ima*2
a/Å	14.025(3)	14.2327(9)	14.5666(7)
b/Å	20.372(4)	20.4021(16)	20.4734(7)
c/Å	8.2283(16)	8.2532(6)	8.2956(3)
α/°	90	90	90
β/°	90	90	90
γ/°	90	90	90
V (Å^3)	2351.0(8)	2396.5(3)	2473.98(17)
Z	4	4	4
ρ_{calc} (g/cm^3)	2.774	2.845	2.882
μ/mm^{-1}	17.860	19.077	18.113
F (000)	1776.0	1848.0	1920.0
Radiation	MoKα (λ = 0.71073)	MoKα (λ = 0.71073)	MoKα(λ=0.71073)
range (°)	5.34 to 52.738	5.324 to 63.034	5.298 to 50.048
Index ranges	$-17 \leq h \leq 17$<br>$-25 \leq k \leq 24$<br>$-10 \leq l \leq 10$	$-20 \leq h \leq 18$<br>$-23 \leq k \leq 30$<br>$-12 \leq l \leq 12$	$-17 \leq h \leq 17$<br>$-24 \leq k \leq 24$<br>$-9 \leq l \leq 9$
Reflections collected	14461	20883	15481
Independent reflections	2505 [R_{int} = 0.0630, R_{sigma} = 0.0457]	4134 [R_{int} = 0.0543, R_{sigma} = 0.0487]	2233 [R_{int} = 0.0479, R_{sigma} = 0.0351]
Data/restraints/parameters	2505/1/159	4134/1/158	2233/21/166
GOF on F^2	1.049	1.042	1.075
R_1, wR_2 ($I > 2\sigma(I)$)[a]	0.0285/0.0665	0.0340/0.0772	0.0208/0.0492
R_1, wR_2 (all data)	0.0344/0.0694	0.0417/0.0805	0.0238/0.0501
Largest diff. peak/hole (e Å^{-3})	1.74/-1.53	2.43/-2.54	1.18/-0.93

$R_1=\sum(||F_0|-|F_c||) / \sum|F_0|$; $wR_2=\{\sum[w(F_0^2-F_c^2)^2]/\sum[w(F_0^2)]^2\}^{1/2}$

Table S2. Atomic coordinates (× 10^4), equivalent isotropic displacement parameters (Å^2 × 10^3) for each atom in the asymmetric unit of TJU-50(Cl)-Br. U_{eq} is defined as one third of the trace of the orthogonalized U_{ij} tensor.

Atom	*x*	*y*	*z*	U_{eq}(Å^2)
Pb1	18.5(2)	20.4(2)	22.1(2)	2.4(4)
Br1	61.0(16)	110(2)	34.1(11)	-33.8(13)
Br2	56.0(14)	72(2)	14.5(8)	0
Cl1	20(2)	81(3)	37(2)	5(3)
O1	16(5)	33(5)	36(5)	-1(4)
O2	20(5)	57(6)	27(5)	-9(4)
O3	53(6)	21(4)	33(6)	4(3)
O4	60(6)	13(4)	30(4)	-5(4)
C1	29(9)	33(8)	18(9)	0
C2	35(7)	16(5)	21(6)	-7(4)
C3	32(7)	12(5)	18(6)	-6(4)
C4	19(10)	14(9)	33(10)	0
C5	27(7)	21(6)	27(6)	0(5)
C6	16(6)	18(6)	31(7)	-2(5)
C7	23(7)	24(6)	28(6)	0(5)
C8	22(7)	35(6)	31(7)	-8(5)
C9	24(9)	41(9)	15(9)	-11(6)
C10	17(9)	12(7)	46(11)	9(7)
N1	54(12)	34(9)	49(10)	-4(8)
C11	63(16)	51(12)	90(20)	-20(15)
C12	59(18)	100(20)	70(18)	1(16)

Table S3. Atomic coordinates (× 10^4), equivalent isotropic displacement parameters (Å^2 × 10^3) for each atom in the asymmetric unit of TJU-50(Br)-Br. U_{eq} is defined as one third of the trace of the orthogonalized U_{ij} tensor.

Atom	*x*	*y*	*z*	U_{eq}(Å^2)
Pb1	799.3(2)	-7344.2(2)	5012.5(5)	21.55(9)
Br1	2500	-7084.6(12)	2466(2)	49.3(4)
Br2	0	-5000	12627(2)	52.7(6)
Br3	-2500	-6358.8(14)	-2606(2)	63.1(6)
O1	17(5)	-6883(4)	2135(8)	28.8(15)
O4	496(7)	-6704(4)	7616(10)	41.5(17)
O3	910(6)	-6103(4)	5522(9)	38.3(18)
O2	-772(5)	-7021(6)	4430(9)	41(2)
C4	0	-5000	6985(16)	34(4)
C8	-2500	-6863(7)	2910(16)	27(3)
C5	593(7)	-6166(5)	6930(11)	25.4(17)
C3	300(7)	-5560(4)	7843(10)	25.7(17)
C9	-1645(7)	-6609(5)	424(11)	30(2)
C10	-2500	-6524(7)	-354(14)	30(3)
C6	-742(6)	-6902(5)	2925(11)	21.8(17)
C2	285(7)	-5556(5)	9538(9)	26.9(19)
C1	0	-5000	10344(13)	31(3)
C7	-1645(6)	-6771(4)	2082(10)	23.7(16)
N1	-2500	-4500(8)	3850(20)	58(4)
C11	-2500	-5071(11)	4990(30)	85(8)
C12	-2500	-4680(20)	2000(30)	94(9)

Table S4. Atomic coordinates (× 10^4), equivalent isotropic displacement parameters (Å^2 × 10^3) for each atom in the asymmetric unit of TJU-50(I)-Br. U_{eq} is defined as one third of the trace of the orthogonalized U_{ij} tensor.

Atom	*x*	*y*	*z*	U_{eq}(Å^2)
Pb1	704.7(2)	-7324.5(2)	16(8)	22.57(14)
I1	2500	-6944(7)	2644(18)	52.2(17)
Br1	-2500	-6427.1(12)	7664(8)	57.8(6)
Br2	0	-5000	-7664(9)	70.8(8)
O1	-46(5)	-6894(4)	2888(12)	30.1(19)
O4	388(7)	-6707(4)	-2664(14)	45(2)
O3	842(6)	-6116(4)	-602(12)	40(2)
O2	-814(5)	-6989(5)	595(12)	39(2)
C1	0	-5000	-5397(18)	33(4)
C4	0	-5000	-2060(20)	33(5)
C5	512(8)	-6176(5)	-2017(15)	34(3)
C2	239(8)	-5560(5)	-4591(14)	32(3)
C3	261(9)	-5558(5)	-2936(15)	31(3)
C9	-1666(8)	-6625(5)	4588(15)	28(3)
C7	-1664(6)	-6755(5)	2979(15)	23(2)
C10	-2500	-6565(8)	5370(20)	35(4)
C8	-2500	-6835(9)	2110(20)	33(4)
C6	-785(7)	-6882(5)	2107(16)	27(2)
N1	-2500	-4538(10)	804(15)	95(7)
C11	-2500	-4770(20)	2490(20)	178(17)
C12	-2500	-5117(11)	-270(30)	108(10)
I2	2500	-7140(50)	2560(30)	71(8)

Table S5. Selected bond lengths for TJU-50(Cl)-Br.

Atom	Atom	Length/Å	Atom	Atom	Length/Å
Pb1	Pb1[1]	4.1551(8)	O2	C6	1.284(15)
Pb1	Pb1[2]	4.1550(8)	O3	C5	1.256(15)
Pb1	Cl1	3.114(4)	O4	C5	1.253(15)
Pb1	Cl1[3]	3.283(4)	C1	C2[4]	1.372(13)
Pb1	O1[2]	2.609(8)	C1	C2	1.372(13)
Pb1	O1	2.779(8)	C2	C3	1.384(14)
Pb1	O2	2.384(8)	C3	C4	1.388(13)
Pb1	O3	2.578(9)	C3	C5	1.517(16)
Pb1	O4	2.562(10)	C6	C7	1.481(16)
Pb1	O4[1]	2.790(9)	C7	C8	1.391(16)
Pb1	C5	2.915(12)	C7	C10	1.421(15)
Pb1	C6	2.950(12)	C8	C9	1.376(14)
Br1	C9	1.888(14)	N1	C11	1.47(3)
Br2	C1	1.905(16)	N1	C12	1.50(3)
O1	C6	1.268(14)			

Symmetry codes for TJU-50(Cl)-Br: [1]+X, -3/2-Y, 1/2+Z; [2]+X, -3/2-Y, -1/2+Z; [3]1/2-X, -3/2-Y, -1/2+Z; [4]1-X, -1-Y, +Z.

Table S6. Selected bond lengths for TJU-50(Br)-Br.

Atom	Atom	Length/Å	Atom	Atom	Length/Å
Pb1	Pb1[1]	4.1753(3)	O2	C3	1.244(12)
Pb1	Pb1[2]	4.1753(3)	O3	C3	1.253(12)
Pb1	Br1	3.2489(13)	O4	C7	1.267(12)
Pb1	Br1[3]	3.3644(12)	C1	C4	1.410(10)
Pb1	O1[1]	2.607(7)	C1	C4[4]	1.410(10)
Pb1	O1	2.787(7)	C2	C10	1.409(11)
Pb1	O2[2]	2.804(7)	C2	C10[5]	1.409(11)
Pb1	O2	2.551(8)	C3	C4	1.506(13)
Pb1	O3	2.572(8)	C4	C8	1.400(11)
Pb1	O4	2.380(7)	C5	C6	1.387(11)
Pb1	C3	2.894(9)	C5	C10	1.408(13)
Pb1	C7	2.931(8)	C7	C10	1.486(12)
Br2	C9	1.884(11)	C8	C9	1.377(11)
Br3	C6	1.889(11)	N1	C11	1.50(2)
O1	C7	1.262(10)	N1	C12	1.57(3)

Symmetry codes for TJU-50(Br)-Br: [1]+X, -3/2-Y, 1/2+Z; [2]+X, -3/2-Y, -1/2+Z; [3]1/2-X, -3/2-Y, 1/2+Z; [4]-X, -1-Y, +Z; [5]-1/2-X, +Y, +Z.

Table S7. Selected bond lengths for TJU-50(I)-Br.

Atom	Atom	Length/Å	Atom	Atom	Length/Å
Pb1	Pb1^{1}	4.20969(18)	O2	C12	1.237(14)
Pb1	Pb1^{2}	4.20969(18)	O3	C12	1.292(16)
Pb1	I1^{3}	3.5586(13)	O4	C8	1.277(15)
Pb1	I1	3.4529(13)	C11	C2	1.369(13)
Pb1	O1	2.765(9)	C11	C2^{4}	1.368(13)
Pb1	O1^{2}	2.626(8)	C1	C3	1.395(14)
Pb1	O2	2.594(10)	C1	C3^{4}	1.395(14)
Pb1	O2^{1}	2.795(9)	C12	C3	1.512(16)
Pb1	O3	2.542(9)	C2	C3	1.379(15)
Pb1	O4	2.361(8)	C4	C5	1.365(16)
Pb1	C12	2.919(11)	C4	C6	1.385(14)
Pb1	C8	2.931(11)	C5	C7	1.408(14)
Br1	C6	1.906(16)	C5	C8	1.496(14)
Br2	C11	1.886(15)	N3	C9	1.68(6)
O1	C8	1.260(13)	N3	C10	1.38(4)

Symmetry codes for TMOF-12(I)-Br: ^{1}+*x*,-3/2-*y*,1/2+*z*; ^{2}+*x*,-3/2-*y*,-1/2+*z*; ^{3}1/2-*x*,-3/2-*y*,-1/2+*z*; ^{4}-*x*,-1-*y*,+*z*.

Table S8. Crystal data and structure refinement for TJU-50(Cl)-Cl, TJU-50(Br)-Cl and TJU-50(I)-Cl.

Identification code	TJU-50(Cl)-Cl	TJU-50(Br)-Cl	TJU-50(I)-Cl
Empirical formula	$C_{18}H_{14}Cl_2NO_8Pb_2Cl$	$C_{18}H_{14}Cl_2NO_8Pb_2Br$	$C_{18}H_{14}Cl_2NO_8Pb_2I$
Formula weight	893.03	937.49	984.50
Tem (K)	300	298	293
Crystal system	orthorhombic	orthorhombic	orthorhombic
Space group	*Ima*2	*Ima*2	*Ima*2
a/Å	13.9887(7)	14.1860(5)	14.5002(7)
b/Å	20.3676(10)	20.3850(8)	20.4953(12)
c/Å	8.0877(4)	8.1053(3)	8.1339(4)
α/°	90	90	90
β/°	90	90	90
γ/°	90	90	90
V (Å^3)	2304.3(2)	2343.90(15)	2417.3(2)
Z	4	4	4
ρ_{calc} (g/cm^3)	2.574	2.657	2.705
μ/mm^{-1}	14.986	16.323	15.452
F (000)	1632.0	1704.0	1776.0
Radiation	MoKα (λ = 0.71073)	MoKα (λ = 0.71073)	MoKα (λ = 0.71073)
2Θ range (°)	5.42 to 56.616	5.408 to 56.624	5.388 to 56.548
Index ranges	$-18 \leq h \leq 18$<br>$-27 \leq k \leq 27$<br>$-10 \leq l \leq 10$	$-18 \leq h \leq 18$<br>$-27 \leq k \leq 27$<br>$-10 \leq l \leq 10$	$-19 \leq h \leq 18$<br>$-27 \leq k \leq 27$<br>$-10 \leq l \leq 10$
Reflections collected	20533	29158	14647
Independent reflections	2944 [R_{int} = 0.0545, R_{sigma} = 0.0379]	3038 [R_{int} = 0.0502, R_{sigma} = 0.0283]	3045 [R_{int} = 0.0507, R_{sigma} = 0.0498]
Data/restraints/parameters	2944/1/160	3038/1/159	3045/1/149
GOF on F^2	1.047	1.092	1.023
R_1, wR_2 ($I > 2\sigma(I)$)[a]	0.0278/0.0755	0.0192/0.0443	0.0289/0.0602
R_1, wR_2 (all data)	0.0309/0.0772	0.0210/0.0452	0.0388/0.0643
Largest diff. peak/hole (e Å^{-3})	1.17/-1.64	1.21/-1.25	1.58/-1.07

$R_1=\sum(||F_0|-|F_c||) / \sum|F_0|$; $wR_2=\{\sum[w(F_0^2-F_c^2)^2]/\sum[w(F_0^2)]^2\}^{1/2}$

Table S9. Atomic coordinates (× 10^4), equivalent isotropic displacement parameters (Å^2 × 10^3) for each atom in the asymmetric unit of TJU-50(Cl)-Cl. U_{eq} is defined as one third of the trace of the orthogonalized U_{ij} tensor.

Atom	*x*	*y*	*z*	U_{eq}(Å^2)
Pb01	5881.7(2)	7341.8(2)	7267.5(10)	21.55(11)
Cl2	7500	7037(3)	9784(5)	47.0(9)
Cl3	2500	6346(3)	14751(5)	63.4(14)
Cl1	5000	5000	-320(6)	51.6(15)
O2	5951(4)	6093(4)	6766(9)	36.6(18)
O3	5063(5)	6887(3)	10120(9)	27.7(14)
O1	5567(7)	6697(4)	4599(10)	40.0(17)
O4	4265(5)	7028(5)	7788(9)	39.5(18)
C5	5651(8)	6153(4)	5308(11)	26.4(18)
C1	5000	5000	1846(13)	26(3)
C6	4293(7)	6899(5)	9301(13)	25.1(18)
C2	5309(6)	5556(4)	2663(10)	26.9(19)
C3	5325(6)	5550(4)	4370(10)	24.2(17)
C10	2500	6513(7)	12636(15)	32(3)
C9	3368(6)	6608(4)	11847(11)	30(2)
C8	2500	6862(8)	9356(18)	29(3)
C7	3363(6)	6773(4)	10187(11)	24.1(17)
C4	5000	5000	5200(18)	23(3)
N1	7500	5463(7)	8447(19)	46(3)
C11	7500	4873(8)	7360(40)	64(5)
C12	7500	5300(13)	10260(20)	67(6)

Table S10. Atomic coordinates (× 10^4), equivalent isotropic displacement parameters (Å^2 × 10^3) for each atom in the asymmetric unit of TJU-50(Br)-Cl. U_{eq} is defined as one third of the trace of the orthogonalized U_{ij} tensor.

Atom	*x*	*y*	*z*	U_{eq}(Å^2)
Pb1	4174.8(2)	2330.0(2)	4092.0(5)	21.67(7)
Cl2	5000	0	-3519(4)	53.2(10)
Cl1	7500	1395(2)	11608(3)	59.6(9)
O3	4469(4)	1697(2)	1402(6)	38.8(11)
O4	4060(3)	1089(2)	3546(6)	35.4(11)
O2	5765(3)	2003(3)	4612(6)	39.4(13)
O1	4974(3)	1889(2)	6947(5)	28.8(10)
C6	4379(5)	1150(3)	2087(8)	27.3(13)
C4	7500	1848(5)	6178(12)	26.1(18)
C5	5733(4)	1889(3)	6140(8)	24.4(13)
C8	4685(4)	555(3)	1159(7)	25.0(12)
C9	4702(4)	555(3)	-552(7)	28.7(14)
C3	6648(4)	1763(3)	7019(8)	24.4(12)
C1	7500	1542(5)	9498(10)	31(2)
Br1	2500	2008.5(7)	6673.6(12)	42.0(3)
C10	5000	0	-1370(9)	27.6(19)
C2	6651(4)	1618(3)	8693(7)	28.4(14)
C7	5000	0	2010(12)	25(2)
N1	2500	425(5)	5144(13)	50(2)
C12	2500	-125(6)	3990(20)	72(4)
C11	2500	218(8)	6927(16)	63(4)

Table S11. Atomic coordinates (× 10^4), equivalent isotropic displacement parameters (Å^2 × 10^3) for each atom in the asymmetric unit of TJU-50(I)-Cl. U_{eq} is defined as one third of the trace of the orthogonalized U_{ij} tensor.

Atom	*x*	*y*	*z*	U_{eq}(Å^2)
Pb1	-4263.1(2)	-7307.8(2)	2423.4(9)	24.14(10)
I1	-2500	-8068.1(8)	4761.4(14)	52.9(4)
Cl1	-7500	-6468(3)	-5131(6)	56.3(13)
Cl2	-5000	-5000	10097(7)	74(2)
O1	-5034(5)	-6899(4)	-434(10)	32.5(18)
O4	-4116(6)	-6093(4)	3113(9)	39.0(19)
O3	-4568(7)	-6702(4)	5195(11)	43(2)
O2	-5805(5)	-6966(5)	1898(9)	40(2)
C8	-5000	-5000	4580(20)	28(4)
C2	-6665(7)	-6642(5)	-2202(12)	29(2)
C4	-7500	-6824(8)	328(18)	28(3)
C1	-7500	-6568(8)	-3023(17)	30(3)
C10	-4452(8)	-6156(6)	4505(13)	31(2)
C3	-6671(7)	-6761(5)	-516(13)	27(2)
C9	-4737(8)	-5554(5)	5466(13)	32(2)
C6	-5000	-5000	7979(16)	34(4)
C5	-5763(7)	-6878(6)	369(13)	28(2)
C7	-4753(8)	-5562(5)	7153(14)	37(3)
N1	-2500	-4882(18)	1950(40)	167(14)
C12	-2500	-5422(15)	3060(30)	86(8)
C11	-2500	-5200(30)	380(60)	190(20)

Table S12. Selected bond lengths for TJU-50(Cl)-Cl.

Atom	Atom	Length/Å	Atom	Atom	Length/Å
Pb01	O2	2.576(7)	C1	C2[3]	1.381(10)
Pb01	O3	2.737(7)	C1	C2	1.381(10)
Pb01	O3[1]	2.607(7)	C6	C7	1.508(12)
Pb01	O1	2.564(8)	C2	C3	1.381(11)
Pb01	O1[2]	2.753(7)	C3	C4	1.383(10)
Pb01	O4	2.388(7)	C10	C9[4]	1.385(11)
Cl3	C10	1.744(13)	C10	C9	1.385(11)
Cl1	C1	1.752(12)	C9	C7	1.383(13)
O2	C5	1.258(11)	C8	C7[4]	1.393(11)
O3	C6	1.264(11)	C8	C7	1.393(11)
O1	C5	1.253(12)	N1	C11	1.49(2)
O4	C6	1.252(13)	N1	C12	1.50(2)
C5	C3	1.514(12)			

Symmetry codes for TJU-50(Cl)-Cl: [1]+X, 3/2-Y, -1/2+Z; [2]+X, 3/2-Y, 1/2+Z; [3]1-X, 1-Y, +Z; [4]1/2-X, +Y, +Z.

Table S13. Selected bond lengths for TJU-50(Br)-Cl.

Atom	Atom	Length/Å	Atom	Atom	Length/Å
Pb1	O3	2.568(5)	C6	C8	1.491(8)
Pb1	$O3^{1}$	2.759(5)	C4	C3	1.398(8)
Pb1	O4	2.573(5)	C4	$C3^{3}$	1.398(8)
Pb1	O2	2.389(4)	C5	C3	1.504(8)
Pb1	O1	2.729(4)	C8	C9	1.387(8)
Pb1	$O1^{2}$	2.616(5)	C8	C7	1.398(7)
Pb1	Br1	3.2331(8)	C9	C10	1.378(7)
Cl2	C10	1.742(8)	C3	C2	1.389(9)
Cl1	C1	1.736(8)	C1	$C2^{3}$	1.379(7)
O3	C6	1.253(8)	C1	C2	1.379(7)
O4	C6	1.272(8)	N1	C12	1.462(16)
O2	C5	1.261(9)	N1	C11	1.505(16)
O1	C5	1.259(7)			

Symmetry codes for TJU-50(Br)-Cl: ^{1}+X, 1/2-Y, 1/2+Z; ^{2}+X, 1/2-Y, -1/2+Z; ^{3}3/2-X, +Y, +Z.

Table S14. Selected bond lengths for TJU-50(I)-Cl.

Atom	Atom	Length/Å	Atom	Atom	Length/Å
Pb1	Pb1[1]	4.1425(2)	O3	C10	1.263(14)
Pb1	Pb1[2]	4.1425(2)	O2	C5	1.258(13)
Pb1	I1	3.5468(10)	C8	C9[4]	1.398(14)
Pb1	I1[3]	3.4377(10)	C8	C9	1.398(14)
Pb1	O1	2.711(8)	C2	C1	1.390(12)
Pb1	O1[1]	2.632(8)	C2	C3	1.394(15)
Pb1	O4	2.562(9)	C4	C3[5]	1.390(12)
Pb1	O3[2]	2.757(8)	C4	C3	1.390(12)
Pb1	O3	2.612(9)	C10	C9	1.518(14)
Pb1	O2	2.382(7)	C3	C5	1.520(14)
Pb1	C10	2.918(11)	C9	C7	1.372(15)
Pb1	C5	2.880(10)	C6	C7[4]	1.380(13)
Cl1	C1	1.727(15)	C6	C7	1.380(13)

Symmetry codes for TJU-50(I)-Cl: [1]+X, -3/2-Y, 1/2+Z; [2]+X, -3/2-Y, -1/2+Z; [3]-1/2-X, -3/2-Y, -1/2+Z; [4]1-X, -1-Y, +Z; [5]-3/2-X, +Y, +Z.

Table S15. Crystal data and structure refinement for TJU-50(Cl)-F, TJU-50(Br)-F and TJU-50(I)-F.

Identification code	TJU-50(Cl)-F	TJU-50(Br)-F	TJU-50(I)-F
Empirical formula	$C_{18}H_{14}F_2NO_8Pb_2Cl$	$C_{18}H_{14}F_2NO_8Pb_2Br$	$C_{18}H_{14}F_2NO_8Pb_2I$
Formula weight	860.13	904.60	951.60
Tem (K)	298	300	300
Crystal system	orthorhombic	orthorhombic	orthorhombic
Space group	*Ima*2	*Ima*2	*Ima*2
a/Å	13.9543(5)	14.1364(5)	14.4323(5)
b/Å	20.4298(8)	20.4680(7)	20.5758(6)
c/Å	7.7857(3)	7.7930(3)	7.8021(2)
α/°	90	90	90
β/°	90	90	90
γ/°	90	90	90
V (Å^3)	2219.57(15)	2254.86(14)	2316.88(12)
Z	4	4	4
ρ_{calc} (g/cm^3)	2.574	2.665	2.728
μ/mm^{-1}	15.332	16.746	15.906
F (000)	1568.0	1640.0	1712.0
Radiation	MoK$_\alpha$ (λ = 0.71073)	MoK$_\alpha$ (λ = 0.71073)	MoKα (λ = 0.71073)
2Θ range (°)	5.6 to 53.536	5.594 to 60.066	5.584 to 56.556
Index ranges	$-17 \leq h \leq 16$<br>$-25 \leq k \leq 25$<br>$-9 \leq l \leq 9$	$-19 \leq h \leq 19$<br>$-28 \leq k \leq 28$<br>$-10 \leq l \leq 10$	$-19 \leq h \leq 19$<br>$-27 \leq k \leq 27$<br>$-10 \leq l \leq 10$
Reflections collected	11190	14870	13389
Independent reflections	2445 [R_{int} = 0.0421, R_{sigma} = 0.0394]	3386 [R_{int} = 0.0485, R_{sigma} = 0.0451]	2805 [R_{int} = 0.0459, R_{sigma} = 0.0426]
Data/restraints/parameters	2445/1/149	3387/1/149	2805/1/158
GOF on F^2	1.084	1.038	1.028
R_1, wR_2 ($I > 2\sigma(I)$)^a	0.0238/0.0504	0.0250/0.0516	0.0215/0.0481
R_1, wR_2 (all data)	0.0286/0.0527	0.0294/0.0533	0.0230/0.0488
Largest diff. peak/hole (e Å^{-3})	0.76/-1.05	0.62/-2.44	0.92/-1.30

$R_1=\sum(||F_0|-|F_c||) / \sum|F_0|$; $wR_2=\{\sum[w(F_0^2-F_c^2)^2]/\sum[w(F_0^2)]^2\}^{1/2}$

Table S16. Atomic coordinates (× 10^4), equivalent isotropic displacement parameters ($Å^2$ × 10^3) for each atom in the asymmetric unit of TJU-50(Cl)-F. U_{eq} is defined as one third of the trace of the orthogonalized U_{ij} tensor.

Atom	*x*	*y*	*z*	$U_{eq}(Å^2)$
Pb1	5917.2(2)	2307.1(2)	-1239.9(13)	20.77(10)
Cl1	7500	1944(2)	1348(4)	41.2(9)
F1	2500	3607(6)	938(11)	57(3)
F2	5000	0	-8684(15)	66(4)
O3	5603(6)	1687(4)	-4075(9)	36.0(18)
O4	5990(5)	1069(4)	-1854(8)	36.1(18)
O1	4267(4)	3010(4)	-5833(8)	37(2)
O2	5063(4)	3093(4)	-3375(8)	27.6(15)
C9	5325(6)	550(4)	-6130(20)	30(2)
C4	2500	3153(7)	-4201(17)	23(3)
C7	5340(7)	546(4)	-4328(11)	24.1(19)
C6	5672(7)	1129(5)	-3378(13)	30(2)
C1	2500	3448(7)	-790(14)	30(3)
C3	3361(6)	3222(4)	-3317(11)	23.6(19)
C2	3367(6)	3376(5)	-1562(15)	30(3)
C5	4286(6)	3109(5)	-4252(12)	22.6(19)
C10	5000	0	-6949(16)	34(3)
N1	7500	310(7)	-563(15)	43(3)
C12	7500	129(12)	1280(20)	69(6)
C11	7500	-266(10)	-1730(20)	69(6)
C8	5000	0	-3450(20)	21(3)

Table S17. Atomic coordinates (× 10^4), equivalent isotropic displacement parameters (Å^2 × 10^3) for each atom in the asymmetric unit of TJU-50(Br)-F. U_{eq} is defined as one third of the trace of the orthogonalized U_{ij} tensor.

Atom	*x*	*y*	*z*	U_{eq}(Å^2)
Pb1	-4129.3(2)	-7297.2(2)	8914.5(5)	19.46(7)
Br1	-2500	-8108.7(6)	6594.2(14)	34.9(2)
F1	-7500	-6418(5)	16107(9)	54(2)
F2	-5000	-5000	1460(11)	65(3)
O1	-4429(5)	-6684(2)	6066(7)	34.9(13)
O2	-5751(4)	-6972(3)	9333(7)	38.3(14)
O3	-4966(3)	-6910(3)	11769(6)	25.6(10)
O4	-4019(4)	-6060(3)	8282(7)	35.4(13)
C5	-4332(5)	-6131(3)	6758(9)	25.6(14)
C4	-5000	-5000	6700(14)	22(2)
C8	-7500	-6841(5)	10966(12)	20.6(18)
C6	-5729(5)	-6893(4)	10920(9)	22.5(14)
C9	-6641(5)	-6632(3)	13598(9)	26.5(16)
C2	-4679(5)	-5550(3)	3995(12)	30.4(13)
C1	-5000	-5000	3182(12)	33(2)
C10	-7500	-6552(5)	14390(11)	27(2)
C7	-6645(5)	-6769(3)	11846(9)	20.7(12)
C3	-4667(5)	-5544(3)	5811(9)	25.1(14)
N1	-2500	-5286(5)	9518(12)	42(2)
C12	-2500	-4741(8)	8296(19)	67(4)
C11	-2500	-5080(8)	11300(20)	63(4)

Table S18. Atomic coordinates (× 10^4), equivalent isotropic displacement parameters (Å^2 × 10^3) for each atom in the asymmetric unit of TJU-50(I)-F. U_{eq} is defined as one third of the trace of the orthogonalized U_{ij} tensor.

Atom	*x*	*y*	*z*	U_{eq}(Å^2)
Pb1	-4207.1(2)	-7718.5(2)	2369.2(5)	20.95(7)
I1	-2500	-6829.0(4)	4564.0(10)	38.17(18)
F1	-7500	-6475(4)	117(9)	50.7(19)
F2	-5000	-10000	9877(11)	75(3)
O3	-4505(5)	-8316(2)	5239(7)	37.9(13)
O2	-5021(3)	-6924(2)	4492(7)	27.3(10)
O4	-4045(4)	-8937(2)	3086(7)	37.5(13)
O1	-5788(3)	-6942(3)	6933(7)	39.1(15)
C2	-6665(5)	-6652(3)	2641(10)	28.5(17)
C6	-4383(5)	-8868(3)	4560(10)	26.7(14)
C1	-7500	-6577(5)	1829(14)	30(2)
C4	-7500	-6832(5)	5289(14)	25(2)
C9	-4700(5)	-9449(3)	7314(13)	35.4(16)
C8	-5000	-10000	4624(17)	28(2)
C7	-4696(6)	-9454(3)	5533(10)	30.2(16)
C3	-6664(5)	-6774(3)	4398(10)	24.0(14)
C5	-5767(5)	-6887(3)	5328(10)	22.9(14)
C10	-5000	-10000	8140(13)	38(3)
N1	-2500	-9718(5)	2060(20)	71(5)
C12	-2500	-9979(10)	290(40)	113(10)
C11	-2500	-10216(10)	3350(40)	115(9)

Table S19. Selected bond lengths for TJU-50(Cl)-F.

Atom	Atom	Length/Å	Atom	Atom	Length/Å
Pb1	Cl[1]	3.080(3)	C9	C7	1.401(17)
Pb1	O3[1]	2.694(7)	C9	C10	1.371(12)
Pb1	O3	2.583(7)	C4	C3	1.392(11)
Pb1	O4	2.576(7)	C4	C3[2]	1.392(11)
Pb1	O1[1]	2.413(6)	C7	C6	1.475(14)
Pb1	O2[1]	2.658(6)	C7	C8	1.390(13)
Pb1	O2	2.600(7)	C1	C2	1.358(10)
F1	C1	1.384(14)	C1	C2[2]	1.358(10)
F2	C10	1.350(17)	C3	C2	1.402(14)
O3	C6	1.266(12)	C3	C5	1.500(12)
O4	C6	1.273(11)	N1	C12	1.48(2)
O1	C5	1.248(11)	N1	C11	1.49(2)
O2	C5	1.281(11)			

Symmetry codes for TJU-50(Cl)-F: [1]+X, 1/2-Y, 1/2+Z; [2]1/2-X, +Y, +Z.

Table S20. Selected bond lengths for TJU-50(Br)-F.

Atom	Atom	Length/Å	Atom	Atom	Length/Å
Pb1	Pb1[1]	3.98394(16)	O2	C6	1.247(9)
Pb1	Pb1[2]	3.98392(17)	O3	C6	1.265(8)
Pb1	Br1	3.3664(8)	O4	C5	1.276(9)
Pb1	Br1[3]	3.2181(8)	C5	C3	1.489(10)
Pb1	O1[2]	2.709(5)	C4	C3[4]	1.393(9)
Pb1	O1	2.585(5)	C4	C3	1.392(9)
Pb1	O2	2.410(5)	C8	C7	1.398(8)
Pb1	O3[1]	2.613(5)	C8	C7[5]	1.398(8)
Pb1	O3	2.641(5)	C6	C7	1.504(9)
Pb1	O4	2.585(6)	C9	C10	1.372(8)
Pb1	C5	2.933(7)	C9	C7	1.394(10)
Pb1	C6	2.871(6)	C2	C1	1.369(8)
F1	C10	1.366(11)	C2	C3	1.416(12)
F2	C1	1.342(13)	N1	C12	1.467(18)
O1	C5	1.260(8)	N1	C11	1.45(2)

Symmetry codes for TJU-50(Br)-F: [1]+X, -3/2-Y, -1/2+Z; [2]+X, -3/2-Y, 1/2+Z; [3]-1/2-X, -3/2-Y, 1/2+Z; [4]-1-X, -1-Y, +Z; [5]-3/2-X, +Y, +Z.

Table S21. Selected bond lengths for TJU-50(I)-F.

Atom	Atom	Length/Å	Atom	Atom	Length/Å
Pb1	$Pb1^{1}$	4.00333(13)	O2	C5	1.262(8)
Pb1	$Pb1^{2}$	4.00328(13)	O4	C6	1.257(9)
Pb1	$I1^{3}$	3.4245(7)	O1	C5	1.258(9)
Pb1	I1	3.5145(6)	C2	C1	1.371(9)
Pb1	$O3^{2}$	2.734(5)	C2	C3	1.393(12)
Pb1	O3	2.590(6)	C6	C7	1.495(10)
Pb1	O2	2.607(5)	C4	C3	1.397(9)
Pb1	$O2^{2}$	2.638(5)	C4	$C3^{4}$	1.397(9)
Pb1	O4	2.579(5)	C9	C7	1.389(12)
Pb1	$O1^{2}$	2.410(5)	C9	C10	1.374(9)
Pb1	C6	2.928(7)	C8	$C7^{5}$	1.400(10)
Pb1	$C5^{2}$	2.875(7)	C8	C7	1.400(10)
F1	C1	1.353(13)	C3	C5	1.502(9)
F2	C10	1.355(14)	N1	C12	1.49(3)
O3	C6	1.266(9)	N1	C11	1.44(2)

Symmetry codes for TJU-50(I)-F: ^{1}+X, -3/2-Y, 1/2+Z; ^{2}+X, -3/2-Y, -1/2+Z; ^{3}-1/2-X, -3/2-Y, -1/2+Z; ^{4}-3/2-X, +Y, +Z; ^{5}-1-X, -2-Y, +Z.

Table S22. Crystal data and structure refinement for TJU-50(Cl)-CH_3, TJU-50(Br)-CH_3 and TJU-50(I)-CH_3.

Identification code	TJU-50(Cl)-CH_3	TJU-50(Br)-CH_3	TJU-50(I)-CH_3
Empirical formula	$C_{20}H_{20}NO_8Pb_2Cl$	$C_{18}H_{14}Br_2NO_8Pb_2Br$	$C_{18}H_{14}Br_2NO_8Pb_2I$
Formula weight	852.270	896.721	943.67
Tem (K)	298	298	300
Crystal system	orthorhombic	orthorhombic	orthorhombic
Space group	*Ima*2	*Ima*2	*Ima*2
a/Å	14.1116(3)	14.3508(13)	14.6519(7)
b/Å	20.3845(6)	20.4424(16)	20.5232(9)
c/Å	8.0238(2)	8.0621(7)	8.0842(3)
α/°	90	90	90
β/°	90	90	90
γ/°	90	90	90
V (Å^3)	2308.11(10)	2365.1(4)	2430.95(18)
Z	4	4	4
ρ_{calc} (g/cm^3)	2.453	2.518	2.578
μ/mm^{-1}	14.758	15.984	15.147
F (000)	1548.8	1619.5	1712.0
Radiation	Mo Kα (λ = 0.71073)	Mo Kα (λ = 0.71073)	Mo Kα (λ = 0.71073)
2Θ range (°)	5.46 to 52.72	5.44 to 52.74	5.416 to 54.218
Index ranges	$-17 \leq h \leq 15$<br>$-25 \leq k \leq 25$<br>$-10 \leq l \leq 10$	$-18 \leq h \leq 18$<br>$-26 \leq k \leq 25$<br>$-10 \leq l \leq 10$	$-18 \leq h \leq 18$<br>$-24 \leq k \leq 26$<br>$-10 \leq l \leq 10$
Reflections collected	14413	22347	13781
Independent reflections	2417 [R_{int} = 0.0545, R_{sigma} = 0.0355]	2514 [R_{int} = 0.0287, R_{sigma} = 0.0249]	2771 [R_{int} = 0.0452, R_{sigma} = 0.0428]
Data/restraints/parameters	2417/1/159	2514/1/161	2771/1/149
GOF on F^2	1.046	1.059	1.063
R_1, wR_2 ($I > 2\sigma(I)$)^a	0.0273/0.0615	0.0145/0.0321	0.0295/0.0576
R_1, wR_2 (all data)	0.0342/0.0649	0.0157/0.0326	0.0376/0.0609
Largest diff. peak/hole (e Å^{-3})	1.01/-1.66	1.68/-1.10	1.58/-1.60

$R_1=\sum(||F_0|-|F_c||) / \sum|F_0|$; $wR_2=\{\sum[w(F_0^2-F_c^2)^2]/\sum[w(F_0^2)]^2\}^{1/2}$

Table S23. Atomic coordinates (× 10^4), equivalent isotropic displacement parameters ($Å^2$ × 10^3) for each atom in the asymmetric unit of TJU-50(Cl)-CH_3. U_{eq} is defined as one third of the trace of the orthogonalized U_{ij} tensor.

Atom	*x*	*y*	*z*	$U_{eq}(Å^2)$
Pb1	5856.44(14)	2640.57(12)	6056.72(7)	21.50(9)
Cl1	7500	2912(2)	3575.1(16)	46.0(9)
O1	5041(4)	3112(4)	3241(5)	30.5(16)
O2	4250(4)	2966(4)	5592(5)	39.4(19)
O3	5566(6)	3303(4)	8689(5)	37.1(16)
O4	5941(4)	3900(3)	6490(5)	34.4(17)
C1	2500	3686(11)	-1154(10)	64(6)
C2	2500	3508(7)	669(10)	37(4)
C3	3345(6)	3413(5)	1524(8)	34(2)
C4	3349(5)	3231(4)	3187(8)	24.8(18)
C5	2500	3145(6)	4048(10)	25(3)
C6	4267(5)	3103(5)	4055(8)	24.8(19)
C7	5635(6)	3852(5)	7947(9)	28.1(19)
C8	5322(5)	4447(4)	8870(8)	20.1(17)
C9	5000	5000	8025(10)	26(3)
C10	5297(5)	4450(5)	10607(8)	31(2)
C11	5000	5000	11457(9)	28(3)
C12	5000	5000	13346(10)	54(5)
C13	2500	4904(8)	5871(12)	69(5)
C14	2500	5370(11)	3037(11)	69(6)
N1	2500	5507(6)	4867(8)	45(3)

Table S24. Atomic coordinates (× 10^4), equivalent isotropic displacement parameters (Å^2 × 10^3) for each atom in the asymmetric unit of TJU-50(Br)-CH_3. U_{eq} is defined as one third of the trace of the orthogonalized U_{ij} tensor.

Atom	*x*	*y*	*z*	U_{eq}(Å^2)
Pb1	4212.53(8)	7348.94(6)	3942.49(4)	21.55(4)
O1	5002(2)	6888.3(16)	6760(3)	28.7(7)
C7	5760(3)	6894(2)	5962(4)	23.3(9)
Br1	2500	7061.0(5)	6489.15(19)	51.1(2)
N1	2500	5483(3)	4977(4)	58.6(19)
C3	4717(3)	5548(2)	-631(4)	28.9(11)
O4	4091(2)	6100.0(15)	3489(3)	34.8(8)
C5	4402(3)	6150(2)	2032(4)	29.8(10)
O3	4504(3)	6696.8(16)	1319(3)	41.5(9)
C10	6672(3)	6603(2)	8514(4)	31.2(11)
C11	7500	6521(3)	9363(5)	30.3(15)
C12	7500	6360(5)	11189(5)	63(3)
C1	5000	5000	-3375(5)	52(2)
C13	2500	4918(4)	3861(6)	78(3)
C14	2500	5308(7)	6806(6)	87(3)
C9	7500	6837(3)	5987(5)	26.9(13)
C8	6659(3)	6763(2)	6835(4)	25.0(9)
C6	5000	5000	1961(5)	28.0(15)
C4	4702(3)	5551(2)	1107(4)	25.9(9)
O2	5784(2)	7012.1(19)	4417(3)	38.7(9)
C2	5000	5000	-1509(5)	29.7(15)

Table S25. Atomic coordinates (× 10^4), equivalent isotropic displacement parameters (Å^2 × 10^3) for each atom in the asymmetric unit of TJU-50(I)-CH_3. U_{eq} is defined as one third of the trace of the orthogonalized U_{ij} tensor.

Atom	*x*	*y*	*z*	U_{eq}(Å^2)
Pb1	-4298.7(2)	-7322.7(2)	4124.9(11)	25.23(14)
I1	-2500	-6936.6(12)	1510.1(19)	74.3(6)
O1	-4624(8)	-6705(4)	6825(12)	42(2)
O2	-5057(5)	-6900(4)	1302(11)	30(2)
O3	-5820(6)	-6968(5)	3646(9)	42(2)
O4	-4151(7)	-6103(4)	4735(10)	43(2)
C11	-5000	-5000	9670(20)	40(5)
C7	-4497(9)	-6157(6)	6173(15)	33(3)
C6	-5812(8)	-6868(6)	2090(14)	26(3)
C4	-6682(8)	-6752(6)	1224(14)	26(3)
C3	-6683(8)	-6637(6)	-485(14)	29(3)
C2	-7500	-6559(9)	-1330(20)	34(4)
C5	-7500	-6809(9)	2040(20)	28(4)
C1	-7500	-6455(12)	-3170(30)	52(6)
C8	-4768(9)	-5556(6)	7090(16)	34(3)
C9	-5000	-5000	6270(20)	30(5)
C10	-4783(9)	-5560(6)	8807(13)	34(3)
C12	-5000	-5000	11490(30)	70(8)
N1	-2500	-5475(13)	4720(30)	88(8)
C14	-2500	-4931(16)	3690(40)	103(11)
C13	-2500	-5300(20)	6460(60)	147(15)

Table S26. Selected bond lengths for TJU-50(Cl)-CH_3.

Atom	Atom	Length/Å	Atom	Atom	Length/Å
Pb1	O1[1]	2.598(6)	C10	C11[3]	1.378(9)
Pb1	O1	2.712(5)	O4	C7	1.251(8)
Pb1	O4	2.594(7)	C7	O3	1.272(11)
Pb1	O3[2]	2.735(6)	C7	C8	1.488(11)
Pb1	O3	2.540(5)	C3	C2[4]	1.389(9)
Pb1	O2	2.391(5)	C3	C4	1.385(9)
O1	C6	1.273(9)	C2	C1	1.507(12)
C6	C4	1.493(9)	C12	C11	1.516(11)
C6	O2	1.265(8)	C5	C4	1.394(8)
N1	C13	1.470(17)	C5	C4[4]	1.394(8)
N1	C14	1.495(12)	C9	C8	1.391(9)
C10	C8	1.394(9)	C9	C8[3]	1.391(9)

Symmetry codes for TJU-50(Cl)-CH_3: [1]+X, 1/2-Y, 1/2+Z; [2]+X, 1/2-Y, -1/2+Z; [3]1-X, 1-Y, +Z; [4]1/2-X, +Y, +Z.

Table S27. Selected bond lengths for TJU-50(Br)-CH_3.

Atom	Atom	Length/Å	Atom	Atom	Length/Å
Pb1	O1	2.708(2)	O4	C5	1.261(4)
Pb1	O1[1]	2.610(3)	C5	O3	1.266(5)
Pb1	O4	2.585(3)	C5	C4	1.497(5)
Pb1	O3	2.535(3)	C10	C11	1.382(5)
Pb1	O2	2.389(3)	C10	C8	1.393(5)
O1	C7	1.264(5)	C11	C12	1.509(6)
C7	C8	1.493(5)	C1	C2	1.505(6)
C7	O2	1.269(4)	C9	C8	1.395(4)
N1	C13	1.464(8)	C9	C8[3]	1.395(4)
N1	C14	1.517(7)	C6	C4	1.387(4)
C3	C4	1.401(4)	C6	C4[2]	1.387(4)
C3	C2[2]	1.385(4)			

Symmetry codes for TJU-50(Br)-CH_3: [1]+X, 3/2-Y, -1/2+Z; [2]1-X, 1-Y, +Z.

Table S28. Selected bond lengths for TJU-50(I)-CH_3.

Atom	Atom	Length/Å	Atom	Atom	Length/Å
Pb1	Pb1^{1}	4.1071(2)	O4	C7	1.273(15)
Pb1	Pb1^{2}	4.10712(19)	C11	C10^{4}	1.383(14)
Pb1	I1	3.4700(13)	C11	C10	1.383(14)
Pb1	I1^{3}	3.6020(14)	C11	C12	1.46(3)
Pb1	O1	2.569(10)	C7	C8	1.492(16)
Pb1	O1^{2}	2.769(9)	C6	C4	1.474(16)
Pb1	O2	2.682(9)	C4	C3	1.402(17)
Pb1	O2^{1}	2.622(9)	C4	C5	1.372(14)
Pb1	O3	2.377(8)	C3	C2	1.389(14)
Pb1	O4	2.560(9)	C2	C1	1.50(3)
Pb1	C7	2.925(12)	C8	C9	1.363(15)
Pb1	C6	2.914(13)	C8	C10	1.388(16)
O1	C7	1.256(15)	N1	C14	1.40(3)
O2	C6	1.277(13)	N1	C13	1.45(5)
O3	C6	1.275(14)			

Symmetry codes for TJU-50(I)-CH_3: ^{1}+X, -3/2-Y, 1/2+Z; ^{2}+X, -3/2-Y, -1/2+Z; ^{3}-1/2-X, -3/2-Y, 1/2+Z; ^{4}-1-X, -1-Y, +Z.

Table S29. Crystal data and structure refinement for TJU-51(Cl)-OH, TJU-51(Br)-OH and TJU-51(I)-OH.

Identification code	TJU-51(Cl)-OH	TJU-51(Br)-OH	TJU-51(I)-OH
Empirical formula	$C_{18}H_{16}NO_{10}Pb_2Cl$	$C_{18}H_{16}NO_{10}Pb_2Br$	$C_{18}H_{16}NO_{10}Pb_2I$
Formula weight	855.14	900.61	947.60
Tem (K)	300	300	300
Crystal system	monoclinic	monoclinic	monoclinic
Space group	*Cc*	*Cc*	*Cc*
a/Å	15.6590(18)	15.8034(14)	16.241(2)
b/Å	20.498(2)	20.5617(16)	20.733(3)
c/Å	7.5048(10)	7.5098(6)	7.5406(10)
α/°	90	90	90
β/°	117.001(4)	116.573(3)	116.578(4)
γ/°	90	90	90
V (Å^3)	2146.3(5)	2182.5(3)	2270.8(5)
Z	4	4	4
ρ_{calc} (g/cm^3)	2.646	2.741	2.772
μ/mm^{-1}	15.849	17.294	16.222
F (000)	1564.0	1640.0	1712.0
Radiation	MoKα (λ = 0.71073)	MoKα (λ = 0.71073)	MoKα (λ = 0.71076)
2Θ range (°)	5.782 to 50.7	5.778 to 48.868	5.61 to 50.7
Index ranges	$-18 \le h \le 18$<br>$-24 \le k \le 24$<br>$-9 \le l \le 9$	$-18 \le h \le 18$<br>$-23 \le k \le 23$<br>$-8 \le l \le 8$	$-19 \le h \le 19$<br>$-24 \le k \le 24$<br>$-9 \le l \le 8$
Reflections collected	16258	8388	13187
Independent reflections	3812 [R_{int} = 0.0633, R_{sigma} = 0.0540]	3295 [R_{int} = 0.0472, R_{sigma} = 0.0583]	3925 [R_{int} = 0.0677, R_{sigma} = 0.0772]
Data/restraints/parameters	3812/314/293	3295/284/281	3925/282/273
GOF on F^2	1.066	1.037	1.058
R_1, wR_2 ($I > 2\sigma(I)$)[a]	0.0540/0.1150	0.0384/0.0970	0.0557/0.1203
R_1, wR_2 (all data)	0.0573/0.1174	0.0392/0.0977	0.0690/0.1263
Largest diff. peak/hole (e Å^{-3})	3.63/-3.23	2.60/-1.41	1.70/-2.55

$R_1=\sum(||F_0|-|F_c||) / \sum|F_0|$; $wR_2=\{\sum[w(F_0^2-F_c^2)^2]/\sum[w(F_0^2)]^2\}^{1/2}$

Table S30. Atomic coordinates (× 10^4), equivalent isotropic displacement parameters ($Å^2$ × 10^3) for each atom in the asymmetric unit of TJU-51(Cl)-OH. U_{eq} is defined as one third of the trace of the orthogonalized U_{ij} tensor.

Atom	*x*	*y*	*z*	$U_{eq}(Å^2)$
Cl1	8667(8)	4386(4)	6697(18)	29.0(15)
O1	1109(16)	2540(12)	9480(30)	38(5)
O2	439(16)	4138(10)	3660(30)	28(3)
O3	289(17)	3524(9)	1230(30)	32(4)
O4	1866(15)	4490(11)	2050(30)	27(4)
O5	1143(15)	4404(10)	-1190(30)	30(4)
O6	3840(20)	4020(13)	-2930(40)	47(6)
O7	6825(16)	4160(10)	10630(30)	32(4)
O8	7209(16)	3565(9)	8610(30)	30(4)
O9	6226(15)	4429(11)	4310(30)	30(3)
O10	5406(15)	4366(10)	5930(40)	29(4)
C1	1174(9)	2591(6)	7701(9)	32(5)
C2	743(8)	3076(5)	6275(13)	24(4)
C3	877(9)	3069(5)	4567(12)	26(4)
C4	440(20)	3609(14)	2970(50)	26(4)
C5	1930(20)	4384(13)	510(50)	25(4)
C6	2837(11)	4272(10)	500(30)	28(4)
C11	3653(13)	4322(10)	2330(30)	26(4)
C10	4556(11)	4262(10)	2410(20)	28(4)
C9	4643(11)	4153(10)	680(30)	31(5)
C8	3828(14)	4104(9)	-1150(30)	31(4)
C7	2925(12)	4163(10)	-1240(30)	32(4)
C12	5430(20)	4373(15)	4410(50)	27(4)
C13	6890(20)	3605(14)	9820(50)	29(4)
C14	6546(8)	3002(6)	10399(13)	31(4)
C15	6412(9)	2995(5)	12108(12)	29(5)
C16	6117(10)	2516(6)	8975(9)	36(7)
Pb1	7091(14)	4757(6)	6450(30)	29(4)
Pb4	270(13)	4822(5)	1870(40)	14.4(3)
Pb2	7125(6)	4770.5(4)	7842(14)	16.4(4)
Pb3	230(6)	4818.8(5)	762(14)	14.4(3)
N1	8682(19)	2783(13)	8510(40)	35(5)
C18	8700(30)	2216(13)	9810(50)	32(7)
C17	8620(30)	2561(18)	6690(50)	39(7)

Table S31. Atomic coordinates (× 10^4), equivalent isotropic displacement parameters (Å^2 × 10^3) for each atom in the asymmetric unit of TJU-51(Br)-OH. U_{eq} is defined as one third of the trace of the orthogonalized U_{ij} tensor.

Atom	*x*	*y*	*z*	U_{eq}(Å^2)
Br1	792.5(15)	5647.5(10)	-341(3)	38.5(5)
O1	7510(11)	5643(6)	9030(20)	41(3)
O2	8350(10)	5570(7)	7321(19)	40(3)
O3	6049(11)	5961(8)	170(20)	45(3)
O4	3330(9)	5580(6)	1810(20)	37(3)
O5	4028(10)	5525(8)	5110(20)	42(3)
O7	2413(9)	3523(6)	-701(18)	34(3)
O6	2612(11)	4138(6)	1790(20)	44(3)
O9	3905(10)	861(7)	3670(20)	42(3)
O8	3233(13)	2619(7)	7530(20)	48(3)
O10	4376(11)	1418(7)	1730(20)	46(3)
C16	4006(13)	1368(6)	2960(30)	39(3)
C1	7573(8)	5656(8)	7419(15)	30(3)
C2	6702(5)	5739(6)	5482(11)	32(3)
C7	5806(6)	5680(6)	5386(12)	32(3)
C6	5008(5)	5725(6)	3561(15)	32(3)
C5	5105(5)	5827(6)	1832(12)	34(3)
C4	6000(7)	5885(6)	1928(11)	34(3)
C3	6799(5)	5841(6)	3753(14)	34(3)
C8	4054(7)	5590(9)	3460(30)	33(3)
C9	2651(14)	3616(7)	1150(20)	40(3)
C14	3653(9)	1984(4)	3461(16)	35(3)
C13	3610(9)	2028(4)	5266(14)	38(3)
C12	3255(11)	2586(5)	5732(15)	40(3)
C11	2943(10)	3101(4)	4393(17)	38(3)
C10	2985(9)	3058(5)	2587(16)	38(3)
C15	3340(10)	2499(6)	2122(14)	39(3)
Pb1	9204(7)	5228(4)	9627(19)	27.8(3)
Pb3	2404(9)	5173(5)	2590(30)	46(3)
Pb2	9211.8(8)	5234.0(3)	10899.0(19)	27.8(3)
Pb4	2423.4(8)	5189.5(3)	3874(2)	28.3(3)
N1	5782(12)	2235(8)	1670(20)	42(3)
C18	5725(18)	2460(11)	-260(30)	48(5)
C17	5848(16)	2774(11)	3030(30)	44(4)

Table S32. Atomic coordinates (× 10^4), equivalent isotropic displacement parameters (Å^2 × 10^3) for each atom in the asymmetric unit of TJU-51(I)-OH. U_{eq} is defined as one third of the trace of the orthogonalized U_{ij} tensor.

Atom	*x*	*y*	*z*	U_{eq}(Å^2)
Pb1	4380(6)	4787.8(6)	4484(14)	20.1(4)
Pb2	4320(18)	4790(12)	5500(50)	20.1(4)
Pb3	967(6)	4752.1(6)	1305(14)	19.1(4)
Pb4	892(18)	4765(11)	190(60)	19.1(4)
I1	2657(6)	4311.8(12)	58(14)	34.7(6)
O1	4297(19)	3504(11)	4920(40)	38(5)
O2	4587(17)	4161(11)	7440(40)	32(4)
O3	5255(15)	5568(11)	7420(40)	29(5)
O4	5940(17)	4413(13)	5660(40)	31(4)
O5	688(19)	4144(11)	4010(40)	35(4)
O6	1135(19)	3577(10)	2240(40)	35(4)
O7	-684(17)	4343(14)	-550(40)	34(4)
O8	127(16)	4431(11)	-2220(40)	27(4)
O9	70(20)	2435(15)	8050(50)	60(7)
O10	-2180(20)	4056(13)	-9440(40)	46(6)
C9	4520(20)	3607(3)	6703(12)	32(5)
C8	5955(2)	4362(11)	4064(6)	24(5)
C4	455(5)	3026(2)	3955(8)	33(4)
C5	166(5)	2509(2)	2645(8)	34(5)
C6	-148(6)	1948(2)	3151(11)	37(5)
C1	-172(6)	1904(2)	4966(11)	39(5)
C2	117(6)	2422(3)	6276(10)	41(5)
C3	431(5)	2983(2)	5770(8)	36(5)
C7	784(6)	3629(3)	3342(12)	35(4)
C10	-612(5)	4420(10)	-2201(10)	32(4)
C11	-1471.0(13)	4281(7)	-4089(3)	26(4)
C12	-2335(2)	4321(8)	-4139(7)	22(4)
C13	-3123.2(11)	4266(9)	-5934(10)	25(5)
C14	-3047(3)	4172(9)	-7679(8)	30(5)
C15	-2182(5)	4133(9)	-7630(5)	26(4)
C16	-1394(3)	4187(8)	-5835(6)	28(5)
N1	2660(40)	2751(15)	2550(90)	77(13)
C17	2680(50)	2270(20)	3820(100)	98(16)
C18	2630(40)	2510(20)	750(110)	88(14)

Table S33. Selected bond lengths for TJU-51(Cl)-OH.

Atom	Atom	Length/Å	Atom	Atom	Length/Å
Cl1	Pb1	2.508(19)	O9	Pb2	2.47(2)
Cl1	Pb4[1]	2.942(18)	O9	Pb2[3]	2.70(2)
Cl1	Pb2	3.012(7)	O10	C12	1.16(4)
Cl1	Pb3[2]	3.050(7)	O10	Pb1	2.61(3)
O1	C1	1.39(2)	O10	Pb2	2.55(2)
O2	C4	1.20(3)	C1	C2	1.3898(9)
O2	Pb4	1.87(3)	C1	C15[5]	1.3898(9)
O2	Pb3	2.47(2)	C2	C3	1.3898(9)
O3	C4	1.23(4)	C3	C4	1.54(3)
O3	Pb4	2.71(2)	C3	C16[5]	1.3897(9)
O3	Pb3	2.673(19)	C4	Pb4	2.59(3)
O4	C5	1.23(4)	C5	C6	1.44(3)
O4	Pb4	2.53(3)	C6	C11	1.3900
O4	Pb3	2.39(2)	C6	C7	1.3900
O5	C5	1.31(3)	C11	C10	1.3900
O5	Pb4[3]	2.17(3)	C10	C9	1.3900
O5	Pb3[3]	2.62(2)	C10	C12	1.52(3)
O5	Pb3	2.61(2)	C9	C8	1.3900
O6	C8	1.36(3)	C8	C7	1.3900
O7	C13	1.32(4)	C12	Pb1	2.47(3)
O7	Pb1[4]	2.29(2)	C12	Pb2	2.85(3)
O7	Pb2	2.66(2)	C13	C14	1.49(3)
O7	Pb2[4]	2.66(2)	C14	C15	1.3898(9)
O8	C13	1.22(4)	C14	C16	1.3898(9)
O8	Pb2	2.528(19)	N1	C18	1.51(4)
O9	C12	1.29(3)	N1	C17	1.41(4)
O9	Pb1	1.70(3)			

Symmetry codes for TJU-51(Cl)-OH: [1]1+X, 1-Y, 1/2+Z; [2]1+X, +Y, 1+Z; [3]+X, 1-Y, -1/2+Z; [4]+X, 1-Y, 1/2+Z; [5]-1/2+X, 1/2-Y, -1/2+Z.

Table S34. Selected bond lengths for TJU-51(Br)-OH.

Atom	Atom	Length/Å	Atom	Atom	Length/Å
Br1	Pb1[1]	2.644(11)	O9	Pb2[3]	2.717(14)
Br1	Pb3	2.698(14)	O8	C12	1.370(14)
Br1	Pb2[1]	3.148(2)	O10	C16	1.29(2)
Br1	Pb4	3.202(2)	O10	Pb2[4]	2.499(14)
O1	C1	1.26(2)	C16	C14	1.5000(15)
O1	Pb1	2.652(19)	C1	C2	1.4999(15)
O1	Pb2	2.556(15)	C1	Pb1	2.518(14)
O2	C1	1.27(2)	C1	Pb2	2.869(7)
O2	Pb1	1.795(17)	C2	C7	1.3900
O2	Pb2	2.505(13)	C2	C3	1.3900
O2	Pb2[2]	2.651(14)	C7	C6	1.3900
O3	C4	1.361(15)	C6	C5	1.3900
O4	C8	1.25(2)	C6	C8	1.4997(15)
O4	Pb3	1.99(2)	C5	C4	1.3900
O4	Pb4[2]	2.568(13)	C4	C3	1.3900
O4	Pb4	2.661(13)	C8	Pb3	2.538(16)
O5	C8	1.27(2)	C8	Pb4	2.853(8)
O5	Pb3	2.518(19)	C9	C10	1.4999(15)
O5	Pb4	2.380(14)	C14	C13	1.3900
O7	C9	1.28(2)	C14	C15	1.3900
O7	Pb4[2]	2.668(12)	C13	C12	1.3900
O6	C9	1.19(2)	C12	C11	1.3900
O6	Pb3	2.274(18)	C11	C10	1.3900
O6	Pb4[2]	2.493(15)	C10	C15	1.3900
O9	C16	1.21(2)	N1	C18	1.49(3)
O9	Pb1[3]	2.335(16)	N1	C17	1.48(3)
O9	Pb2[4]	2.669(13)			

Symmetry codes for TJU-51(Br)-OH: [1]-1+X, +Y, -1+Z; [2]+X, 1-Y, -1/2+Z; [3]-1/2+X, 1/2-Y, -1/2+Z; [4]-1/2+X, -1/2+Y, -1+Z.

Table S35. Selected bond lengths for TJU-51(I)-OH.

Atom	Atom	Length/Å	Atom	Atom	Length/Å
I1	$Pb2^{1}$	3.17(2)	O8	$Pb3^{1}$	2.71(2)
I1	Pb4	3.06(3)	O8	Pb3	2.48(2)
I1	Pb1	3.404(3)	O9	C3	1.37(3)
I1	Pb3	3.399(3)	O10	C15	1.38(3)
O1	C1	1.25(3)	C1	$C5^{3}$	1.5100
O1	Pb2	2.70(3)	C1	Pb2	2.58(3)
O1	Pb1	2.69(2)	C2	$C13^{4}$	1.51(3)
O2	C1	1.26(2)	C2	Pb1	2.854(11)
O2	Pb2	1.86(4)	C7	C6	1.3900
O2	Pb1	2.47(2)	C7	C8	1.3900
O3	$C2^{2}$	1.26(2)	C7	C9	1.5100
O3	Pb2	2.24(3)	C6	C5	1.3900
O3	Pb1	2.59(2)	C5	C4	1.3900
O3	$Pb1^{2}$	2.64(2)	C4	C3	1.3900
O4	C2	1.22(2)	C3	C8	1.3900
O4	Pb2	2.70(3)	C10	C11	1.5100
O4	Pb1	2.41(2)	C10	Pb4	2.41(3)
O5	C9	1.22(2)	C10	Pb3	2.823(9)
O5	$Pb4^{2}$	2.40(3)	C11	C12	1.3900
O5	Pb3	2.60(2)	C11	C16	1.3900
O6	C9	1.21(3)	C12	C13	1.3900
O6	Pb3	2.52(2)	C13	C14	1.3900
O7	C10	1.31(3)	C14	C15	1.3900
O7	Pb4	2.52(3)	C15	C16	1.3900
O7	Pb3	2.55(2)	N1	C17	1.38(7)
O8	C10	1.21(2)	N1	C18	1.43(8)
O8	Pb4	1.82(4)			

Symmetry codes for TJU-51(I)-OH: ^{1}+X, 1-Y, -1/2+Z; ^{2}+X, 1-Y, 1/2+Z; ^{3}1/2+X, 1/2-Y, 1/2+Z; ^{4}1+X, +Y, 1+Z.

Table S36. Calculated local dipole moments for PbI_2O_6 as well as the net dipole moment for a unit cell of TJU-50(I)-Br.

groups	Dipole moment (*D*)			
	Total magnitude	*x*-component	*y*-component	z-component
PbI_2O_6	-0.94	0.58	0.60	-0.43
	-1.96	1.47	0.26	1.26
	-0.94	0.58	0.60	-0.43
	-0.94	-0.58	0.60	0.43
	-1.96	-1.47	0.26	1.26
	-0.94	-0.58	-0.61	0.43
	-1.96	1.47	-0.26	1.26
	-0.94	0.58	0.60	-0.43
	-1.52	0.58	-0.60	1.26
	-0.94	-0.58	-0.61	-0.43
	-1.96	-1.47	-0.26	1.26
	-0.94	-0.58	-0.60	-0.43
sum	5.04	0	0	5.04

Table S37. Calculated local dipole moments for PbI_2O_6 as well as the net dipole moment for a unit cell of TJU-51(I)-OH.

groups	Dipole moment (*D*)			
	Total magnitude	*x*-component	*y*-component	z-component
PbI_2O_6	4.14	-3.10	-2.74	0.03
	4.61	-2.64	3.69	0.81
	4.15	3.11	-2.74	0.03
	4.14	-3.10	2.74	0.03
	4.61	2.64	-3.69	0.81
	-1.24	-0.74	-0.76	0.66
	-1.25	0.74	0.76	0.66
	4.14	3.10	2.74	0.03
sum	3.05	0	0	3.05

Table S38. Powder SHG responses and bandgaps of nonlinear optical crystals for hybrid metal halide corresponding to Fig. 3g.

**No.**	**Compounds**	**Space group**	**SHG response @1064 nm (×KDP)**	**Bandgap (eV)**	**Ref**
1	TJU-50(I)-Br	*Ima*2	11.0	3.701	**This work**
2	TJU-50(I)-Cl	*Ima*2	10.3	3.792	**This work**
3	TJU-50(I)-F	*Ima*2	10.1	3.781	**This work**
4	TJU-50(I)-CH_3	*Ima*2	10.0	3.839	**This work**
5	TJU-50(Br)-Br	*Ima*2	9.5	4.039	**This work**
6	TJU-50(Br)-Cl	*Ima*2	9.0	4.052	**This work**
7	TJU-50(Br)-Cl	*Ima*2	8.9	4.066	**This work**
8	TJU-50(Br)-F	*Ima*2	8.8	4.039	**This work**
9	TJU-51(I)-OH	*Cc*	8.5	3.758	**This work**
10	TJU-50(Cl)-Cl	*Ima*2	8.4	4.079	**This work**
11	TJU-50(Cl)-F	*Ima*2	8.2	4.106	**This work**
12	TJU-50(Br)-CH_3	*Ima*2	8.0	4.090	**This work**
13	TJU-50(Cl)-CH_3	*Ima*2	7.2	4.160	**This work**
14	TJU-51(Br)-OH	*Cc*	7.0	3.974	**This work**
15	TJU-51(Cl)-OH	*Cc*	6.0	4.012	**This work**
16	$Cs_3Pb_2(CH_3COO)_2I_5$	*Amm*2	8.0	2.57	ref[13]
17	$K_2I[PbI(OOCCH_2COO)]$	*Amm*2	6.3	3.34	ref[14]
18	$Cs_3Pb_2(CH_3COO)_2Br_5$	*Amm*2	4.0	3.26	ref[13]
19	$KPb_2(C_7H_3NO_4)_2I$	*I*-43*d*	3.05	3.4	ref[15]
20	$CsPb_2(C_7H_3NO_4)_2I$	*I*-43*d*	3.06	2.4	ref[15]
21	$RbPb_2(C_7H_3NO_4)_2I$	*I*-43*d*	3.06	1.6	ref[15]
22	(L-ipp)(L-pro)PbI_3	$P2_1$	2.75	8.0	ref[16]
23	$(C_{10}H_{11}N_3)PbBr_4$	*Cc*	2.95	4.3	ref[17]
24	$(C_{10}H_{11}N_3)PbCl_4$	*Cc*	3.35	3.8	ref[17]

25	(R/S-3-aminopiperidine)PbI_4	$P2_12_12_1$	2.94	2.1	ref[18]
26	[R-MP]PbI_3	$P2_12_12_1$	2.95	1.6	ref[19]
27	(R/S-2-MPD)$PbBr_3$	$P2_12_12_1$	3.51	1.4	ref[20]
28	(L-Hpro)(L-pro)PbI_3	$P2_1$	2.57	1.3	ref[16]
29	($C_4H_{10}NO$)$PbBr_3$	$P2_12_12_1$	3.6	0.8	ref[21]
30	($C_4H_{10}NO$)$PbCl_3$	$P2_12_12_1$	3.55	0.7	ref[21]
31	[$(C_5H_6N_2)_2H$](Sb_4F_{13})	$P1$	3.05	8.1	ref[22]
32	$MAGeBr_3$	$R3m$	2.91	5.3	ref[23]
33	L-H_2his·$SbBr_5$·H_2O	$P2_12_12_1$	2.73	2.6	ref[24]
34	$NH(CH_3)_3SnBr_3$	$Pna2_1$	2.76	2.5	ref[25]
35	$NH(CH_3)_3SnCl_3$	$Cmc2_1$	3.59	1.0	ref[25]
36	$FAGeBr_3$	$R3m$	3.13	0.9	ref[23]
37	$(TMA)_3Sb_2Br_9$	$R3c$	2.95	0.8	ref[26]
38	$(TMA)_3Sb_2Cl_9$	Pc	3.28	0.7	ref[26]

L-ipp = L-N-isopropylidene-proline; L-pro = L-proline; R-MP = R-2-methylpiperidinium; R/S-2-MPD = R/S-2-methylpiperidine; L-H_2his = L-histidine; TMA = $NH(CH_3)_3^+$

Table S39. Calculated linear and nonlinear optical properties for TJU-50(Cl)-Br, TJU-50(Br)-Br and TJU-51(I)-Br deduced from real-space atom-cutting analyses.[a]

		Total	PbI_2O_6	ipa	$(CH_3)_2NH_2^+$
TJU-50(I)-Br	n_x	2.006	1.695	1.756	1.587
	n_y	1.802	1.664	1.629	1.508
	n_z	2.161	1.789	1.985	1.564
	Δn@546 nm	0.359	0.125	0.356	0.056
	d_{15}	1.847	0.879	1.568	0.258
	d_{24}	-0.790	-0.456	-0.897	-0.238
	d_{33}	0.629	0.456	0.336	0.365
		Total	PbI_2O_6	ipa	$(CH_3)_2NH_2^+$
TJU-50(Br)-Br	n_x	1.935	1.7658	1.858	1.532
	n_y	1.785	1.643	1.687	1.521
	n_z	2.126	1.799	2.065	1.534
	Δn@546 nm	0.341	0.156	0.378	0.013
	d_{15}	-1.371	-0.895	-1.236	-0.145
	d_{24}	0.704	0.589	0.898	0.036
	d_{33}	-0.256	-0.365	-0.236	-0.268
		Total	$PbBr_2O_6$	ipa	$(CH_3)_2NH_2^+$
TJU-50(Cl)-Br	n_x	1.903	1.802	1.985	1.608
	n_y	1.784	1.633	1.748	1.596
	n_z	2.114	1.812	2.113	1.625
	Δn@546 nm	0.330	0.179	0.365	0.029
	d_{15}	1.277	0.569	1.458	0.293
	d_{24}	-0.796	-0.655	-0.779	-0.013
	d_{33}	0.123	0.125	0.283	0.186

[a] *d* values in pm/V.

Table S40. Calculated linear and nonlinear optical properties for TJU-50(I)-Cl, TJU-50(I)-F, TJU-50(I)-CH_3 and TJU-51(I)-OH deduced from real-space atom-cutting analyses.[a]

		Total	PbI_2O_6	ipa	$(CH_3)_2NH_2^+$
TJU-50(I)-Cl	n_x	1.977	1.895	1.981	1.628
	n_y	1.813	1.729	1.598	1.659
	n_z	2.140	1.985	2.006	1.698
	Δn@546 nm	0.327	0.256	0.408	0.039
	d_{15}	-1.935	-0.895	-1.589	-0.568
	d_{24}	0.831	0.665	0.872	0.067
	d_{33}	-0.721	-0.456	-0.402	-0.266
		Total	PbI_2O_6	ipa	$(CH_3)_2NH_2^+$
TJU-50(I)-F	n_x	2.001	1.992	1.856	1.666
	n_y	1.821	1.710	1.724	1.642
	n_z	2.043	1.898	1.992	1.678
	Δn@546 nm	0.222	0.188	0.268	0.036
	d_{15}	-2.066	-1.236	-2.658	-0.562
	d_{24}	1.075	0.885	0.924	0.126
	d_{33}	-0.536	-0.498	-0.556	-0.185
		Total	$PbBr_2O_6$	ipa	$(CH_3)_2NH_2^+$
TJU-50(I)-CH_3	n_x	1.992	1.896	1.907	1.669
	n_y	1.828	1.78	1.779	1.636
	n_z	2.043	1.911	1.978	1.701
	Δn@546 nm	0.215	0.131	0.199	0.065
	d_{15}	-2.026	-1.111	-1.956	-0.456
	d_{24}	1.225	0.568	0.865	0.236
	d_{33}	-0.086	-0.266	-0.125	-0.132
		Total	$PbBr_2O_6$	ipa	$(CH_3)_2NH_2^+$
TJU-51(I)-OH	n_x	2.018	1.922	1.936	1.988
	n_y	1.817	1.855	1.810	1.665
	n_z	2.040	1.978	1.997	1.697
	Δn@546 nm	0.223	0.123	0.187	0.032
	d_{11}	0.434	0.236	0.366	0.032

d_{15}	-2.513	-1.235	-2.567	-0.567
d_{12}	-0.065	-0.036	-0.004	0.036
d_{13}	0.166	0.059	0.011	0.235
d_{24}	1.136	0.654	0.865	0.325
d_{33}	-1.023	-0.567	-0.654	-0.256

[a] *d* values in pm/V.

Reference

1. G. Sheldrick, SHELXT-Integrated space-group and crystal-structure determination, *Acta Crystallogr., Sect. A*, 2015, **71**, 3-8.
2. A. Spek, Single-crystal structure validation with the program PLATON, *J. Appl. Crystallogr.*, 2003, **36**, 7-13.
3. S. K. Kurtz and T. T. Perry, A Powder Technique for the Evaluation of Nonlinear Optical Materials, *J. Appl. Phys.*, 1968, **39**, 3798-3813.
4. M. W. Finnis, Preface, *J. Phys.: Condens. Matter*, 2002, **14**, 001.
5. V. Milman, B. Winkler, J. A. White, C. J. Pickard, M. C. Payne, E. V. Akhmatskaya and R. H. Nobes, Electronic structure, properties, and phase stability of inorganic crystals: A pseudopotential plane-wave study, *Int. J. Quantum Chem*, 2000, **77**, 895-910.
6. B. Delley, Hardness conserving semilocal pseudopotentials, *Physical Review B*, 2002, **66**, 155125.
7. J. P. Perdew, K. Burke and M. Ernzerhof, Generalized Gradient Approximation Made Simple, *Phys. Rev. Lett.*, 1996, **77**, 3865-3868.
8. H. J. Monkhorst and J. D. Pack, Special points for Brillouin-zone integrations, *Phys. Rev. B*, 1976, **13**, 5188-5192.
9. Z. Lin, X. Jiang, L. Kang, P. Gong, S. Luo and M.-H. Lee, First-principles materials applications and design of nonlinear optical crystals, *J. Phys. D: Appl. Phys.*, 2014, **47**, 253001.
10. J. Lin, M.-H. Lee, Z.-P. Liu, C. Chen and C. J. Pickard, Mechanism for linear and nonlinear optical effects in β−BaB_2O_4 crystals, *Phys. Rev. B*, 1999, **60**, 13380-13389.
11. M.-H. Lee, C.-H. Yang and J.-H. Jan, Band-resolved analysis of nonlinear optical properties of crystalline and molecular materials, *Phys. Rev. B,* 2004, **70**, 235110.
12. J. Lu, X. Liu, M. Zhao, X.-B. Deng, K.-X. Shi, Q.-R. Wu, L. Chen and L.-M. Wu, Discovery of NLO Semiorganic $(C_5H_6ON)^+(H_2PO_4)^-$: Dipole Moment Modulation and Superior Synergy in Solar-Blind UV Region, *J. Am. Chem. Soc.*, 2021, **143**, 3647-3654.
13. Q.-R. Shui, H.-X. Tang, R.-B. Fu, Y.-B. Fang, Z.-J. Ma and X.-T. Wu, $Cs_3Pb_2(CH_3COO)_2X_5$ (X=I, Br): Halides with Strong Second-Harmonic Generation Response Induced by Acetate Groups, *Angew. Chem. Int. Ed.*, 2021, **60**, 2116-2119.
14. Z.-Q. Zhou, R.-B. Fu, H.-X. Tang, Z.-J. Ma and X.-T. Wu, An excellent lead oxyiodide with a strong second-harmonic generation response and a large birefringence induced by the oriented arrangement of highly distorted $[PbO_4I_2]$ polyhedra, *Inorg. Chem. Front.*, 2022, **9**, 4464-4469.
15. Z.-L. Geng, Z.-Q. Zhou, H.-X. Tang, W.-X. Bao, R.-B. Fu and X.-T. Wu, $APb_2(C_7H_3NO_4)_2I$ (A = K, Rb, Cs): rare stable nonlinear optical crystals with second-harmonic generation response and highly distorted lead core coordination polyhedra, *Inorg. Chem. Front.*, 2022, **9**, 5783-5787.
16. J. Cheng, G. Yi, Z. Zhang, Y. Long, H. Zeng, L. Huang, G. Zou and Z. Lin, In Situ Chiral Template Approach to Synthesize Homochiral Lead Iodides for Second-Harmonic Generation, *Angew. Chem. Int. Ed.*, 2024, **63**, e202318385.
17. L.-L. Zhang, Q. Ding, P. Wang, Y. Zhang, Q.-Y. Liu, Y.-L. Wang and J. Luo, Achieving strong second harmonic generation effects induced via dimensional increase of PbX_6 octahedra and halogen substitutes in $(C_{10}H_{11}N_3)PbX_4$ (X = Cl or Br), *Inorg. Chem. Front.*, 2024, **11**, 3618-3625.
18. D. Fu, J. Xin, Y. He, S. Wu, X. Zhang, X.-M. Zhang and J. Luo, Chirality-Dependent Second-Order Nonlinear Optical Effect in 1D Organic–Inorganic Hybrid Perovskite Bulk Single Crystal, *Angew. Chem. Int. Ed.*, 2021, **60**, 20021-20026.
19. H. Peng, Y.-H. Liu, X.-Q. Huang, Q. Liu, Z.-H. Yu, Z.-X. Wang and W.-Q. Liao, Homochiral one-dimensional ABX_3 lead halide perovskites with high-Tc quadratic nonlinear optical and dielectric switchings, *Mater. Chem. Front.*, 2021, **5**, 4756-4763.
20. Y. Zheng, J. Xu and X.-H. Bu, 1D Chiral Lead Halide Perovskites with Superior Second-Order Optical Nonlinearity, *Adv. Opt. Mater.*, 2022, **10**, 2101545.
21. C. Shen, D. Sun, Y. Dang, K. Wu, T. Xu, R. Hou, H. Chen, J. Wang and D. Wang, $(C_4H_{10}NO)PbX_3$ (X = Cl, Br): Design of Two Lead Halide Perovskite Crystals with Moderate Nonlinear Optical Properties, *Inorg. Chem.*, 2022, **61**, 16936-16943.
22. J.-H. Wu, C.-L. Hu, Y. Li, J.-G. Mao and F. Kong, $[(C_5H_6N_2)_2H](Sb_4F_{13})$: A polyfluoroantimonite with strong second harmonic generation effect, *Chem. Sci.*, 2024, **15**, 8071-8079
23. Y. Liu, Y.-P. Gong, S. Geng, M.-L. Feng, D. Manidaki, Z. Deng, C. C. Stoumpos, P. Canepa, Z. Xiao, W.-X. Zhang and L. Mao, Hybrid Germanium Bromide Perovskites with Tunable Second Harmonic Generation, *Angew. Chem. Int. Ed.*, 2022, **61**, e202208875.
24. J. Cheng, P. Qian, M. Yang, L. Huang, H. Zeng, G. Zou and Z. Lin, Second-Harmonic Generation in Homochiral Antimony Halides Directed by l-Histidine, *Inorg. Chem.*, 2023, **62**, 16673-16676.

25. Y. Dang, C. Zhong, G. Zhang, D. Ju, L. Wang, S. Xia, H. Xia and X. Tao, Crystallographic Investigations into Properties of Acentric Hybrid Perovskite Single Crystals $NH(CH_3)_3SnX_3$ (X = Cl, Br), *Chem. Mater.*, 2016, **28**, 6968-6974.
26. D. Chen, Z. Song, C. Yang, Y. Wei, G. Liu, L. Meng, Q. Wu and Y. Dang, Nonlinear Optical Effects of Hybrid Antimony(III) Halides Induced by Stereoactive $5s^2$ Lone Pairs and Trimethylammonium Cations, *Inorg. Chem.*, 2024, **63**, 10304-10311.
